# Insights into the activation mechanism of class I HDAC complexes by inositol phosphates

**DOI:** 10.1038/ncomms11262

**Published:** 2016-04-25

**Authors:** Peter J. Watson, Christopher J. Millard, Andrew M. Riley, Naomi S. Robertson, Lyndsey C. Wright, Himali Y. Godage, Shaun M. Cowley, Andrew G. Jamieson, Barry V. L. Potter, John W. R. Schwabe

**Affiliations:** 1 Henry Wellcome Laboratories of Structural Biology, Department of Molecular and Cell Biology, University of Leicester, Leicester LE1 9HN, UK; 2 Department of Pharmacy and Pharmacology, University of Bath BA2 7AY, UK; 3 Department of Chemistry, University of Leicester, Leicester LE1 7RH, UK; 4 Department of Pharmacology, University of Oxford, Oxford OX1 3QT, UK

## Abstract

Histone deacetylases (HDACs) 1, 2 and 3 form the catalytic subunit of several large transcriptional repression complexes. Unexpectedly, the enzymatic activity of HDACs in these complexes has been shown to be regulated by inositol phosphates, which bind in a pocket sandwiched between the HDAC and co-repressor proteins. However, the actual mechanism of activation remains poorly understood. Here we have elucidated the stereochemical requirements for binding and activation by inositol phosphates, demonstrating that activation requires three adjacent phosphate groups and that other positions on the inositol ring can tolerate bulky substituents. We also demonstrate that there is allosteric communication between the inositol-binding site and the active site. The crystal structure of the HDAC1:MTA1 complex bound to a novel peptide-based inhibitor and to inositol hexaphosphate suggests a molecular basis of substrate recognition, and an entropically driven allosteric mechanism of activation.

## Abstract (web summary)

Class I histone deacetylase complexes can be activated by inositol phosphates. Here, the authors investigate the stereochemical requirements for activation; use the crystal structure to understand substrate recognition, and suggest an entropically driven mechanism of allostery.

## Graphical abstract

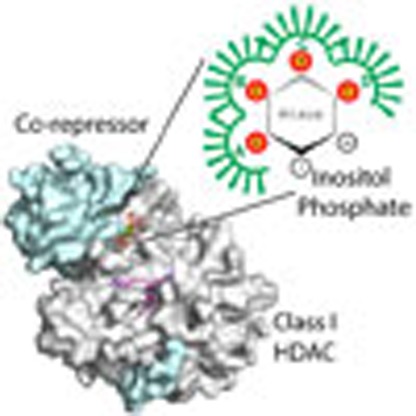

Class I histone deacetylases (HDACs) are enzymes involved in ‘epigenetic' gene regulation through controlling the acetylation state of lysine sidechains in histone tails^1^. They act as the catalytic subunit of several large protein complexes that repress gene expression when targeted to the genome. Recent structural and functional studies of class I HDACs in complex with their cognate co-repressors have suggested that the activity of these complexes is regulated in the cell by inositol phosphates that are likely derived from membrane phospholipids^2^,^3^,^4^. Understanding the regulation of these complexes is important since they are promising targets for epigenetic therapies for a range of diseases^5^. These include numerous cancers as well as spinal muscular atrophy^6^, Friedrich's ataxia^7^, Alzheimer's disease^8^ and HIV infection^9^. Five HDAC inhibitors are now variously licensed for use in the clinic for the treatment of cutaneous T-cell lymphoma, peripheral T-cell lymphoma^10^,^11^ and multiple myeloma^12^.

The class I HDAC family comprises of HDACs 1–3 and 8 (reviewed in ref. 13). HDACs 1–3 are assembled into at least five large multi-protein co-repressor complexes that are recruited to chromatin through interaction with repressive transcription factors or other silencing co-factors^14^. The enzymatic activity of HDACs 1–3 show significant enhancement when incorporated into their cognate co-repressor complexes^15^,^16^,^17^,^18^,^19^,^20^. HDAC8, however, sits alone as the only class I HDAC that is not recruited into a larger complex and is fully active in isolation^21^,^22^. HDACs 1 and 2 are found within several distinct co-repressor complexes including NuRD^23^, Sin3A^24^, CoREST^25^ and MiDAC^4^,^26^. HDAC3, however, is exclusively recruited to the SMRT/NCoR co-repressor complex^20^,^27^. The regulation of these complexes by inositol phosphates was first suggested by the surprising discovery that inositol 1,4,5,6-tetrakisphosphate (Ins(1,4,5,6)P_4_) was present in the HDAC3:SMRT crystal structure^2^. The Ins(1,4,5,6)P_4_ is located at a binding pocket formed at the interface between HDAC3 and the co-repressor. The finding that the Ins(1,4,5,6)P_4_ co-purified with the HDAC3 complex from mammalian cells suggests that it is likely to be a physiologically relevant activator of the complex. However, it is not possible to exclude the possibility that other inositol phosphates might also be able to activate the complex. Indeed, Ins(1,4,5,6)P_4_ is only one of several higher order inositol phosphates which are produced in cells from Ins(1,4,5)P_3_, the well-known second messenger that regulates Ca^2+^ release through binding to the inositol trisphosphate receptor (InsP_3_R) (ref. 28).

Importantly, the key residues which coordinate the binding of Ins(1,4,5,6)P_4_ to the HDAC3:SMRT complex were found to be conserved in several class I HDAC complexes, suggesting that these complexes may also be activated by inositol phosphates. However, it is notable that the key residues are not conserved in the Sin3A co-repressor. Indeed, the structure of the HDAC1:MTA1 complex confirmed that the inositol phosphate-binding pocket was present in other class I HDAC co-repressor complexes^3^.

We initially proposed that Ins(1,4,5,6)P_4_ serves as an ‘inter-molecular glue', mediating interaction between HDAC3 and SMRT^2^. It later emerged that longer constructs of SMRT form a constitutive complex with HDAC3 and that the role of the Ins(1,4,5,6)P_4_ is to activate the HDAC3 enzyme itself^3^. Intriguingly, we observed using mass-spectrometry, that the HDAC3:SMRT complex always co-purifies with Ins(1,4,5,6)P_4_ and that the Ins(1,4,5,6)P_4_ can only be removed using a high-salt wash (resulting in an inactive complex). In contrast, mass-spectrometry showed that the HDAC1:MTA1 complex does not co-purify with Ins(1,4,5,6)P_4_ or any other inositol phosphates. However, the HDAC1:MTA1 complex is nevertheless robustly activated by exogenous Ins(1,4,5,6)P_4_. The novel MiDAC complex has also been shown to be activated by exogenous Ins(1,4,5,6)P_4_ (ref. 4).

The physiological importance of inositol phosphate activation of HDAC complexes is supported by the finding that mutants in the inositol phosphate-binding pocket of HDAC1 are unable to fully restore HDAC activity in HDAC1/2 knock-out ES cells and rescue their viability^29^. Furthermore, mice containing a mutation of one of the key inositol phosphate-binding residues in SMRT (Y470) exhibit increased local histone acetylation *in vivo*^30^.

Whilst the structures of HDACs 1 and 3 in complex with their cognate co-repressors, along with functional studies, have established that these complexes are activated by inositol phosphates^3^, the exact mechanism through which inositol phosphates activate HDACs remains unclear. To address this important issue we have taken a chemical biology approach to understand what are the important stereochemical features of inositol phosphates that are required to activate class I HDAC complexes. We have used inositol phosphate derivatives to directly investigate the binding of inositol phosphates to HDAC complexes *in vitro* and to demonstrate how further derivatives might be developed as tools to modulate HDAC activity. These approaches do not purport to identify which inositol phosphates are relevant *in vivo*. In addition, we have developed a novel peptide-based HDAC inhibitor. This is essentially a substrate mimic based on the histone H4 tail, but incorporating a hydroxamic acid functionality. Using a structural biology approach we have explored the details of substrate recognition, as well as the relationship between the binding of substrate and activation by inositol phosphates. These studies, together with kinetic and mutational analyses of these enzyme complexes, give insight into the mechanism underlying the inositol phosphate-mediated allostery.

## Results

### HDAC3 activation by inositol phosphates

To understand the stereochemical basis underlying the activation of HDAC3 by inositol phosphates we investigated the ability of a range of inositol phosphates and derivatives to enhance the activity of HDAC3 in deacetylase assays. To achieve this we used an assay based on the complex of HDAC3 with an extended SMRT construct which is stable in the absence of inositol phosphate^3^. In this assay, any endogenous inositol phosphate that has been co-purified with the complex is removed by treatment with high-ionic strength buffer, which results in a complex that shows little catalytic activity but can be readily activated by the addition of Ins(1,4,5,6)P_4_ (Supplementary Fig. 1).

**Figure 1:**
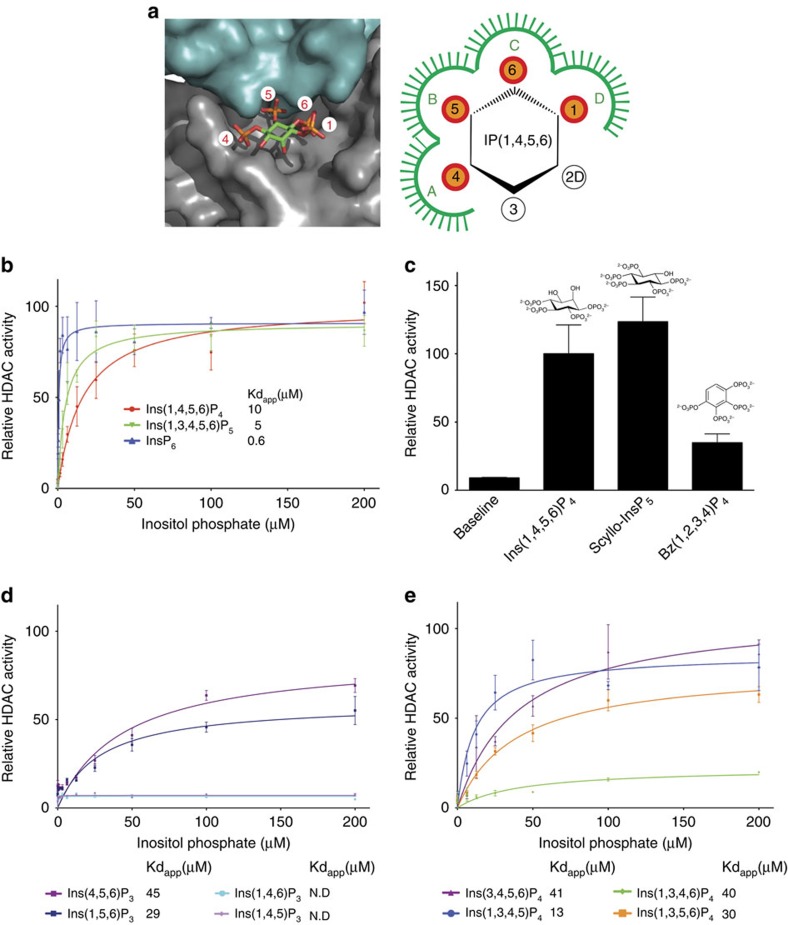
The stereospecific requirements for activation of HDAC activity by various inositol phosphates. (**a**) Ins(1,4,5,6)P_4_ bound to the HDAC3:SMRT complex, with the HDAC3 shown as a grey surface and the SMRT as a cyan surface. Schematic representation of the inositol phosphate-binding pocket, with the binding sites for the four phosphates of Ins(1,4,5,6)P_4_ designated A, B, C and D as indicated. 2D indicates that the axial hydroxyl group on the second position of the inositol ring is pointing down in this view. (**b**,**d**,**e**) Activation of HDAC3 by inositol phosphates using a BOC-Lys-AMC assay. HDAC activity is expressed relative to the maximal Ins(1,4,5,6)P_4_-stimulated activity and is plotted against the inositol phosphate concentration (μM). (**c**) The ability of *scyllo*-InsP_5_ and the planar benzene 1,2,3,4-tetrakisphosphate to activate HDAC3:SMRT is compared with Ins(1,4,5,6)P_4_. HDAC activity was measured in the presence of 200 μM inositol phosphate/benzene tetrakisphosphate. HDAC activity is expressed relative to the maximal Ins(1,4,5,6)P_4_ activity. Error bars indicate±s.e.m. (*n*=3).

The crystal structure of Ins(1,4,5,6)P_4_ bound to the HDAC3:SMRT complex suggests that each of the four phosphates on the inositol ring are recognized by distinct sites within the binding pocket. These sites, referred to as A, B, C and D, accommodate phosphates at positions 4, 5, 6 and 1, respectively, on the inositol ring (Fig. 1a). Positions 2 and 3 of the inositol ring are relatively unobstructed and the free hydroxyls do not appear to be recognized by the protein suggesting that higher order inositol phosphates might also be able to bind and activate the HDAC3:SMRT complex. Indeed, both Ins(1,3,4,5,6)P_5_ and InsP_6_ are able to activate the complex to the same degree as Ins(1,4,5,6)P_4_ (Fig. 1b). Interestingly, they bind with higher apparent affinity than Ins(1,4,5,6)P_4_; Ins(1,3,4,5,6)P_5_ shows a twofold increase and InsP_6_ a 16-fold increase (*K*_d_ values of 5.0 and 0.6 μM, respectively, compared with 10 μM). This higher apparent affinity is mostly likely due to the increased negative charge contributing to a higher on-rate for binding to the positively charged pocket of the HDAC3:SMRT complex. In addition to being able to accommodate a phosphate group on the 2 position of the inositol ring, it also appears that the axial orientation is not important, since *scyllo*-InsP_5_ (ref. 31), which has an equatorial 2-hydroxyl, exhibits similar activity to Ins(1,4,5,6)P_4_ (Fig. 1c).

The stereochemistry of the Ins(1,4,5,6)P_4_-binding pocket suggests that the chair conformation of the inositol is important to position the phosphates correctly. This interpretation is supported by the finding that benzene 1,2,3,4-tetrakisphosphate^32^ (four adjacent phosphates on a planar ring) fails to substantially activate the complex (Fig. 1c).

To fully rationalize the stereochemical requirements for activation of the HDAC3:SMRT complex, we explored the ability of eight tris- and tetrakis-inositol phosphates to enhance the deacetylase activity of the complex. These molecules varied in their ability to activate the HDAC3:SMRT complex and bound with a range of apparent dissociation constants (Fig. 1d,e). Interpreting these data is complicated by there being potentially multiple modes of binding for the various inositol phosphates. Furthermore, some of these modes might support binding to, but not activation of, the complex. Careful analysis allows us to conclude that a minimum of three adjacent phosphates is required for the substantial activation of HDAC3 in the complex. These phosphates must occupy sites A, B and C in the binding pocket (Fig. 1a). Full analyses of these data and conclusions are presented in the Supplementary Materials.

The requirement for three phosphates in positions A, B and C, fits well with the HDAC3:SMRT structure. The phosphates at sites B and C are essentially completely buried at the interface of HDAC3 and the SMRT co-repressor. The requirement for site A to be occupied can also be rationalized, since the phosphate at this site forms a salt bridge with R265 in HDAC3 which has been shown to be essential for activation of the enzyme^2^. It is important to note that the well-established physiologically relevant inositol phosphate signalling molecule, Ins(1,4,5)P_3_, which activates the InsP_3_R to open calcium channels, is completely unable to activate HDAC3. This can be explained as it cannot simultaneously fulfil sites A, B and C.

To complement our analysis based on the ability of different inositol phosphates to activate the HDAC3:SMRT complex, we also performed computational docking studies. We used Autodock to first examine the contribution to the overall binding energy of the phosphates at the 1 or 4 position of Ins(1,4,5,6)P_4_ (Supplementary Fig. 4a–c). Removal of phosphate P1 (occupying site D) reduced the binding energy by just 1.2 kcal mol^−1^; whereas removal of phosphate P4 (occupying site A) reduced the binding energy by 5.4 kcal mol^−1^. This fits well with the activation data, which suggest that site A is more important than site D. We also freely docked Ins(1,3,4,5)P_4_ into the complex, since this molecule activates to greater than 75% yet can only fulfil three adjacent phosphate sites. The three lowest energy-docked molecules adopt an orientation that positions phosphates P3, P4 and P5 in sites A, B and C with an average binding energy of −18.72 kcal mol^−1^ (Supplementary Fig. 4d). This energy is very similar to the calculated binding energy of Ins(1,4,5,6)P_4_ (−18.86 kcal mol^−1^). The majority of the other docking solutions also fulfil positions A, B and C, but with lower binding energies. None of the docking solutions positioned the phosphates in sites B, C and D, leaving the A site unoccupied. Taken together, these computational docking studies fit well with our conclusion that sites A, B and C are essential for both inositol phosphate binding and activation of the complex.

### Activation of the HDAC3:SMRT complex by synthetic analogues

**Figure 2:**
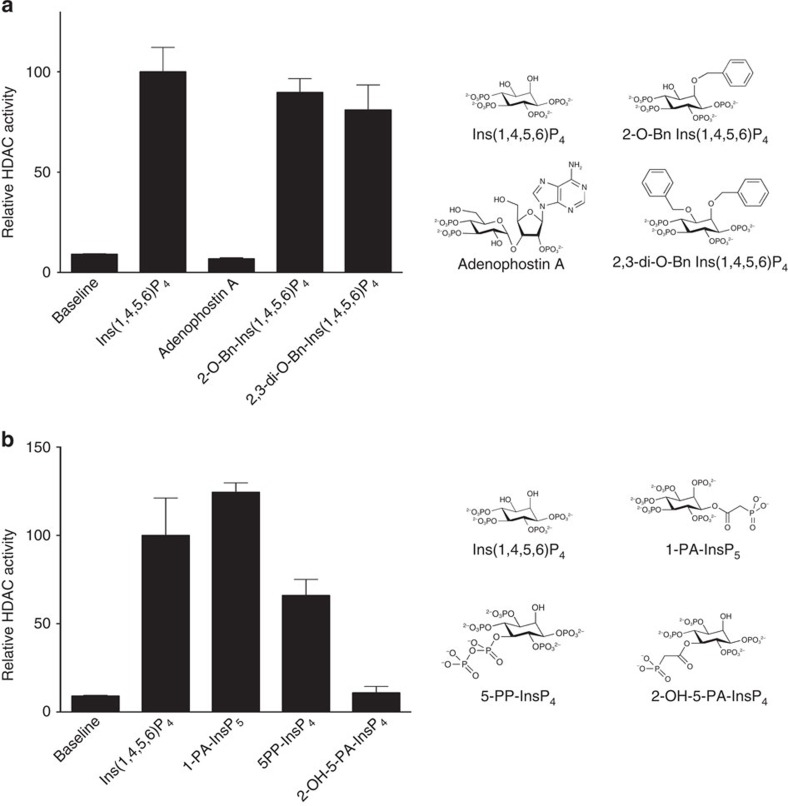
Activation of the HDAC3:SMRT complex by inositol phosphate analogues and derivatives. (**a**) The ability of adenophostin A and inositol phosphates with bulky substituents at the 2 and 3 positions to activate HDAC3:SMRT compared with Ins(1,4,5,6)P_4_. (**b**) Comparison of inositol pyrophosphate and pyrophosphate mimics with Ins(1,4,5,6)P_4_. In all cases, compounds were tested at 200 μM for their ability to stimulate HDAC activity. HDAC activity is expressed as the percentage of maximal Ins(1,4,5,6)P_4_ activity. Error bars indicate±s.e.m. (*n*=3).

To explore further the distinction between molecules that activate the InsP_3_ receptor and those that activate HDACs, we tested the ability of synthetic adenophostin A, a potent InsP_3_ receptor agonist^33^,^34^, to activate the HDAC3:SMRT complex. We observed no activity for adenophostin A even at the high concentration of 200 μM (Fig. 2a). This lack of activity likely results from the fact that the phosphate groups on the pyranose ring of adenophostin A mimic the 4 and 5 position phosphates of Ins(1,4,5)P_3_ and hence cannot satisfy the sites required for activation.

The observation that InsP_6_ is able to activate the HDAC complex, raised the question as to whether larger groups might be added on the 2 and 3 positions of the inositol ring. Accordingly, we synthesized and evaluated inositol phosphates with bulky substituents (benzyl groups) at the 2 and 3 positions (Fig. 2a). Strikingly, bulky groups at these positions can be tolerated with a minimal loss of HDAC activation.

It has recently been reported that inositol pyrophosphates may be important in *S. cerevisiae* for the regulation of the class 1 HDAC homologue Rpd3L (ref. 35). Since the inositol-binding residues identified in HDAC3:SMRT are also present in Rpd3 and the Snt1 co-repressor, we speculated that this regulation might be mediated through the same inositol phosphate-binding pocket. We therefore tested whether pyrophosphate analogues and pyrophosphate, 5-PP-InsP_4_, might be able to activate the HDAC3 complex. The pyrophosphate mimic 1-PA-InsP_5_ (ref. 36) (pyrophosphate mimic on position 1 of the inositol ring) has similar activity to that of Ins(1,4,5,6)P_4_, whereas 5-PP-InsP_4_ (pyrophosphate on position 5) has reduced activity (∼60%) compared with Ins(1,4,5,6)P_4_. In contrast, the pyrophosphate mimic at position 5 in 2-OH-5-PA-InsP_4_ (ref. 37) is completely inert (Fig. 2b). This difference in activity may be due to differences in the possible binding modes of the pyrophosphates and their mimics. 1-PA-InsP_5_ can bind in a way that sites A, B, C and D are all satisfied, whereas 5-PP-InsP_4_ can only satisfy the sites A, B and C. 2-OH-5-PA-InsP_4_, which contains a carbonyl in place of the pyrophosphate of 5-PP-InsP_4_, cannot form the crucial salt bridge with R265 in HDAC3.

### Inositol hexakisphosphate activation of HDAC1

**Figure 3:**
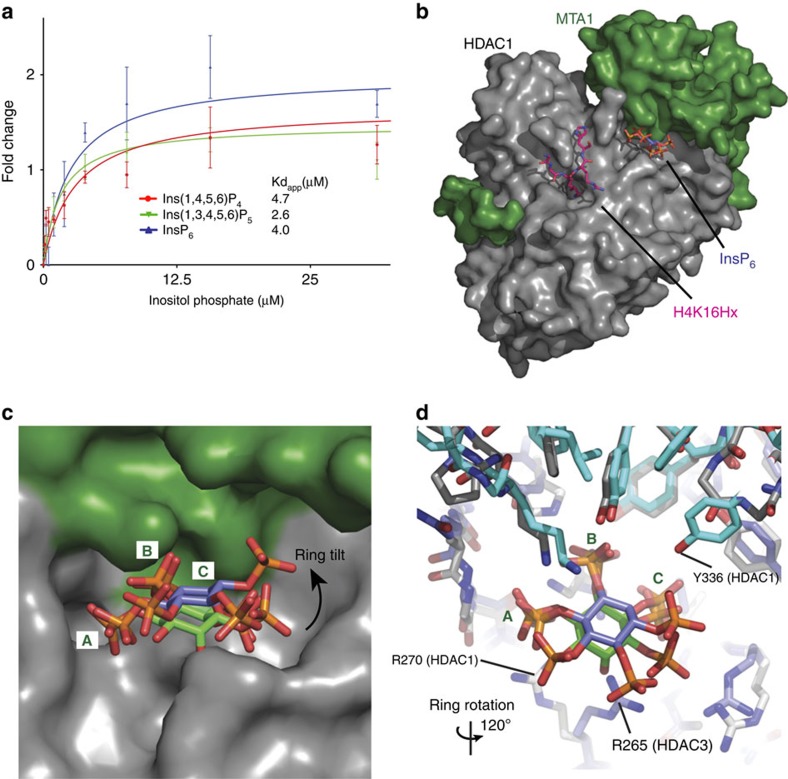
Structural basis for the activation of HDAC1:MTA1 by inositol phosphates. (**a**) HDAC activity of HDAC1:MTA1 is enhanced by Ins(1,4,5,6)P_4_, Ins(1,3,4,5,6)P_5_ and InsP_6_. A BOC-Lys-AMC assay was used - error bars indicate ±s.e.m. (*n*=3). (**b**) Crystal structure of HDAC1:MTA1 with InsP_6_ bound at the interface between the two proteins. The modified histone H4 peptide H4K16Hx (pink) is bound to the active site. (**c**,**d**) Three adjacent phosphates of InsP_6_ (purple) occupy sites A, B and C in an almost identical manner to the Ins(1,4,5,6)P_4_ (green) molecule bound to the HDAC3:SMRT crystal structure. However, InsP_6_ lies in a different orientation to Ins(1,4,5,6)P_4_; the ring is rotated by 120° and is tilted towards MTA1.

We have previously shown that the HDAC1:MTA1 complex can also be activated by Ins(1,4,5,6)P_4_ (ref. 3). Here we show that, as for the HDAC3:SMRT complex, the HDAC1:MTA1 complex can be activated by both Ins(1,3,4,5,6)P_5_ and InsP_6_, suggesting that the mode of inositol phosphate binding is likely to be similar (Fig. 3a). To investigate this, we soaked crystals of the HDAC1:MTA1 complex (which had been co-crystallized with a peptide-based inhibitor) with 225 mM InsP_6_ before freezing for data collection. The structure was solved to 3.3 Å by molecular replacement, by using the structure of unliganded HDAC1:MTA1 (pdb code 4BKX) (Table 1). The structure shows that the InsP_6_ is bound as predicted in the pocket formed at the interface of HDAC1 and MTA1, adjacent to the active site of HDAC1 (Fig. 3b, Supplementary Fig. 5). The occupancy of InsP_6_ in the crystal was refined to 70%.

As might have been anticipated, there are some differences in the mode of InsP_6_ binding when compared with Ins(1,4,5,6)P_4_ binding to the HDAC3:SMRT complex. In particular the inositol ring is tipped away from the HDAC (towards MTA1), which seems to correlate with the sidechain of R270 (HDAC1) adopting a different position from that of the corresponding R265 in HDAC3 (Fig. 3c,d). The molecule is supported in this new position through a hydrogen bond between the hydroxyl of Y336 and the C3 phosphate group of the InsP_6_.

Careful analysis of the orientation of the InsP_6_ molecule at the inter-molecular interface showed that the electron density clearly fits best when the inositol phosphate orientation is rotated by 120° relative to that seen in the HDAC3:SMRT:Ins(1,4,5,6)P_4_ complex (Fig. 3c,d). This rotation allows the phosphate on the C2 position of the inositol to be accommodated in the pocket, and compensates for small structural differences between the two complexes. Despite this difference in orientation, the sites A, B and C (see above) are occupied by phosphates in exactly the same position as in the HDAC3 complex with Ins(1,4,5,6)P_4_; thus, supporting the importance of satisfying these positions so as to activate the enzyme. Interestingly, the phosphates in sites A and B overlap perfectly with the position of the ordered sulphates seen in the HDAC1:MTA1 complex in the absence of inositol phosphate^3^.

### A direct binding assay for inositol phosphates

**Figure 4:**
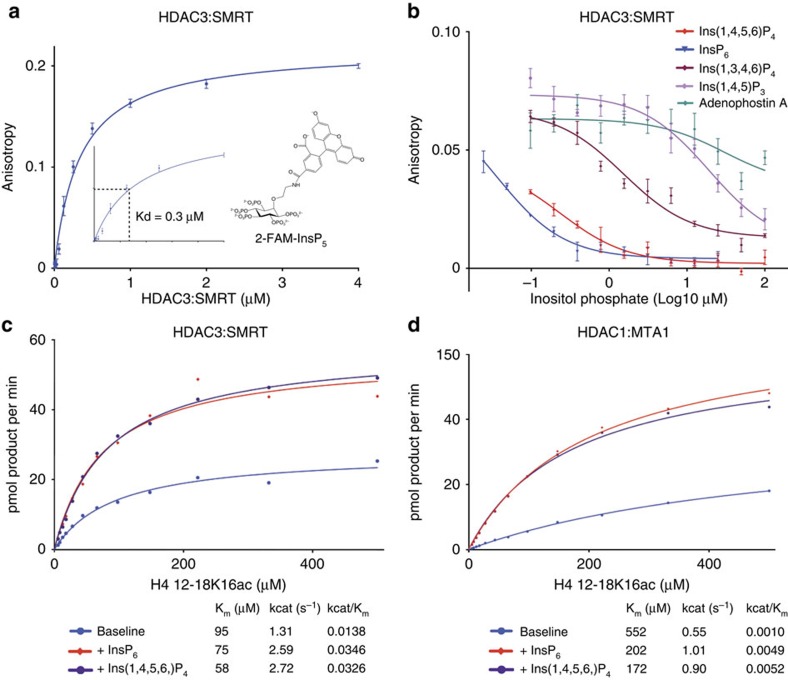
Direct binding of inositol phosphates and effect of inositol phosphate on kinetic parameters. (**a**) Fluorescence anisotropy assay of the binding of 2-FAM-Ins(1,3,4,5,6)P_5_ to HDAC3:SMRT. (**b**) Displacement of 2-FAM-Ins(1,3,4,5,6)P_5_ by various inositol phosphates. IC_50_ values were calculated using Graphpad Prism. (**c**,**d**) Determination of enzyme kinetic parameters in the presence and absence of InsP_6_ and Ins(1,4,5,6)P_4_ for the HDAC3:SMRT and HDAC1:MTA1 complexes with H4 12–18K16ac peptide. Error bars indicate±s.e.m. (*n*=3).

The finding that inositol phosphate analogues derivatized on carbons 2 and 3 of the inositol ring are able to activate the HDAC3:SMRT complex, suggested that it might be possible to use a fluorescence anisotropy assay to directly measure the inositol phosphate binding to the complex. Accordingly, we used a fluorescent derivative of Ins(1,3,4,5,6)P_5_ in which fluorescein was coupled via a linker to the oxygen on carbon 2 of the inositol ring (2-FAM-Ins(1,3,4,5,6)P_5_) (Fig. 4a) (ref. 38). Importantly, the 2-FAM-Ins(1,3,4,5,6)P_5_ activates the HDAC3:SMRT complex to a similar extent as Ins(1,4,5,6)P_4_ (Supplementary Fig. 6).

Interestingly, the dissociation constant measured using this direct binding assay is 0.3 μM (±0.01 μM). This is ∼10-fold tighter than the apparent *K*_d_ value observed in the activation assays, suggesting that binding does not necessarily equate to activation. This is likely due to there being multiple modes of binding to the complex, such that some forms of the bound complex are not fully active. However, we cannot rule out that the fluorescein moiety might contribute in part to the binding.

The concept that binding does not necessarily equate to activation is supported by the results of competition assays. These indicate that certain inositol phosphates, such as Ins(1,3,4,6)P_4_ and Ins(1,4,5)P_3_, which do not activate the complex and cannot simultaneously occupy sites A, B and C, can nevertheless displace the 2-FAM-Ins(1,3,4,5,6)P_5_ with IC_50_ values of 1.5 and 19 μM, respectively (Fig. 4b). We note that the InsP_3_R agonist, adenophostin A^33^,^34^, neither activates HDAC3, nor competes for inositol phosphate binding.

### Inositol phosphates as allosteric regulators of Km and kcat

Lysine deacetylase assays using the minimal substrate Boc-Lys(Ac)-AMC show that both the HDAC3:SMRT and HDAC1:MTA1 complexes are reproducibly activated by inositol phosphates. However, in this assay, the HDAC3 complex appears to be much more sensitive to inositol phosphates giving a 10–15-fold increase in activity, compared with a 2–3-fold activation of the HDAC1 complex. To explore further the differences between the two complexes we established a more physiological, real-time kinetic assay using substrates based on the tails of histones H4 and H3. The peptides tested were; H4 12–18(K16ac), H3 23–29(K27ac) and H3 6–12(K9ac). Interestingly, there is a marked variation in Km, kcat and enzymatic efficiency (kcat/Km) for the various substrates. HDAC3:SMRT is most catalytically active against H3 23–29(K27ac) (Supplementary Fig. 7), whereas HDAC1:MTA1 is most catalytically active against H4 12–18(K16ac) (Fig. 4d). In both cases an approximate 3-fold greater kcat is observed for the preferred versus non-preferred substrate, in the absence of inositol phosphate. In general, inositol phosphates had a modest effect on Km (sometimes lowering, but sometimes increasing), and a more marked effect on kcat (always increasing). The net effect of both inositol phosphates tested was between a two and fivefold increase in catalytic efficiency (kcat/Km) (Fig. 4c,d, Supplementary Fig. 7).

### A novel peptide-based inhibitor mimics substrate binding

**Figure 5:**
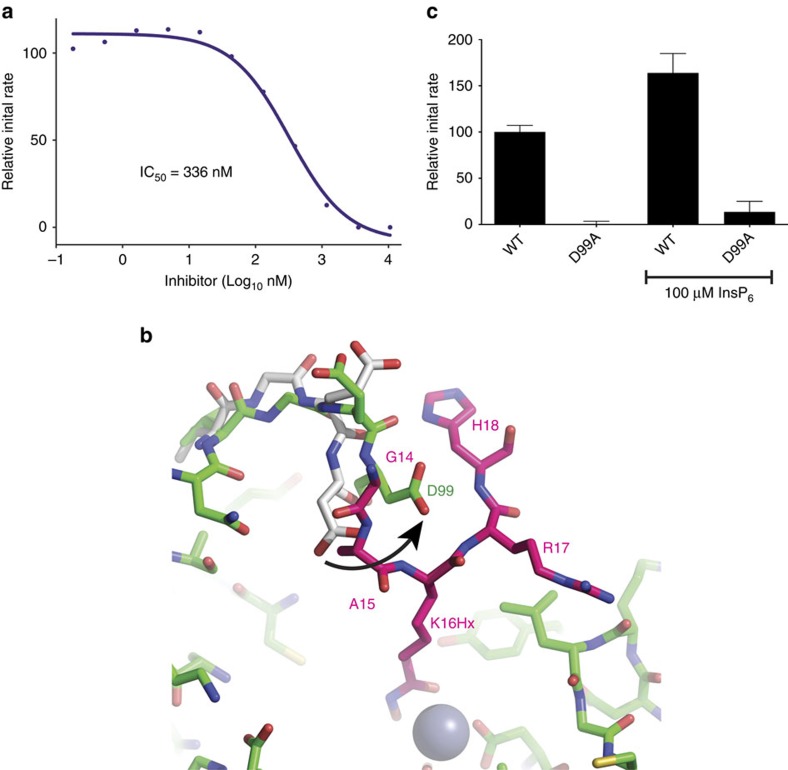
Inhibition of HDAC1:MTA1 by a novel peptide-based inhibitor. (**a**) Inhibition curve of HDAC1:MTA1 by the histone H4 peptide H4K16Hx. (**b**)The position of H4K16Hx (pink) bound to the active site of HDAC1 (purple). The sidechain of HDAC1 residue D99 undergoes a significant rearrangement (indicated by arrow) to coordinate binding of the peptide (apo-HDAC1 shown in grey). (**c**) The D99A HDAC1 mutant does not show deacetylase activity and activity is not rescued on addition of InsP_6_. HDAC assays were performed using an electrophoretic mobility shift assay on the caliper platform. Error bars indicate±s.e.m. (*n*=3).

To understand how a substrate, such as the histone H4 tail, interacts with HDAC1, we synthesized a novel inhibitor based on this peptide. This inhibitor (termed H4K16Hx) comprises residues 12–18 of histone H4 with K16 being replaced by a hydroxamic acid functionality (Supplementary Fig. 8). H4K16Hx is markedly different from the macrocyclic peptide inhibitors^39^ (cyclic peptides derived from natural products), one of which (romidepsin) is licensed for the treatment of cutaneous and peripheral T-cell lymphoma^10^. Inhibition assays show that this peptide inhibits the enzyme with an IC_50_ of 336 nM which is comparable with IC_50_ values reported for other hydroxamic acid-based inhibitors of Zn-dependent HDACs^40^ (Fig. 5a).

We co-crystallized the HDAC1:MTA1 complex with this peptide before soaking it with InsP_6_ (see above). The peptide was observed to be bound at the active site of the enzyme with nearly 100% occupancy, although residues Lys12 and Gly13 were not observed in the electron-density map suggesting that these are not constrained on substrate binding. The hydroxamic acid functionality is buried within the narrow active-site channel with the carbonyl oxygen coordinating the Zn^2+^ in a similar manner to that observed with other class I HDAC inhibitors (for example trichostatin A (TSA) and SAHA bound to HDAC8: pdb codes 1T69 and 1T64, respectively). Several backbone amides in the H4K16 peptide make complementary polar contacts with the sidechain of D99 at the rim of the HDAC active site (Fig. 5b, Supplementary Fig. 5). Interestingly, D99 is conserved in all class I and class II HDACs. Comparison with the structure in the absence of the histone peptide suggests that this residue, and its immediate neighbours, undergo a conformation change on peptide binding. The critical importance of D99 is supported by the finding that mutation to alanine results in total loss of catalytic activity (Fig. 5c).

### Mechanism of class I HDAC activation by inositol phosphates

**Figure 6:**
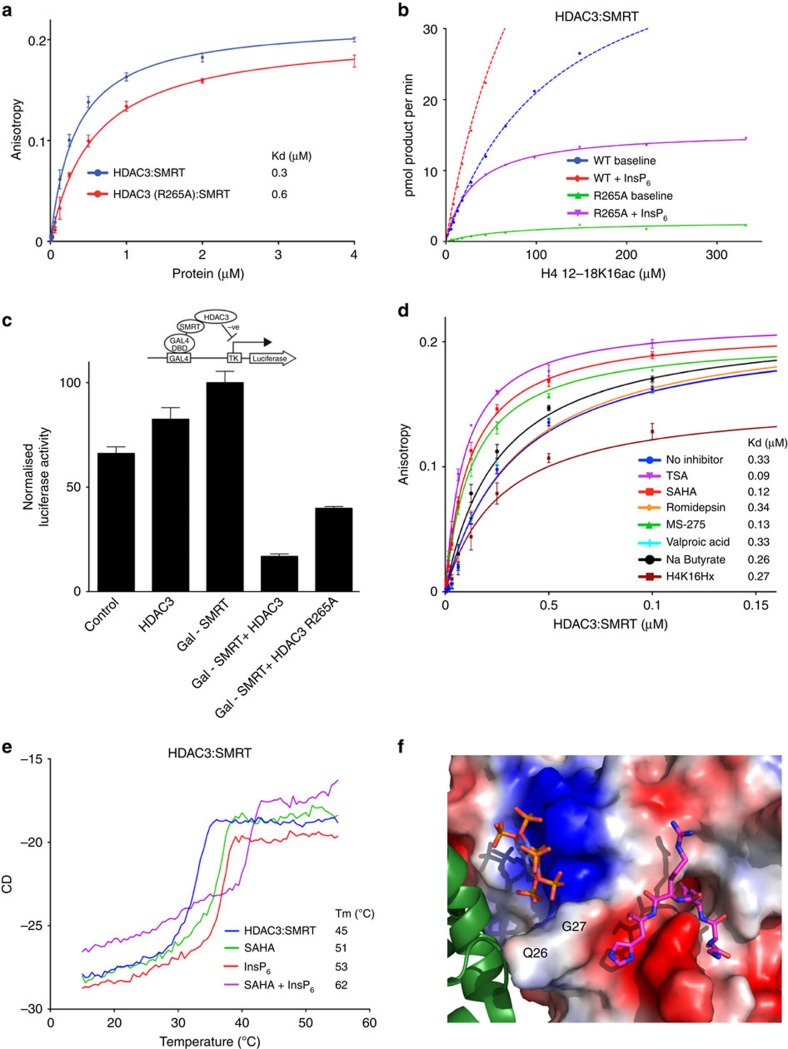
Exploring the mechanism of activation by inositol phosphates. (**a**) Comparison of the binding of 2-FAM-Ins(1,3,4,5,6)P_5_ to HDAC3:SMRT and HDAC3 (R265A):SMRT. (**b**) Measurement of Km and kcat in the presence and absence of InsP_6_, for HDAC3 (R265A):SMRT. Data for the wild-type HDAC3:SMRT are also shown for comparison (N.B. the data are presented on a different scale from Fig. 4c). (**c**) Repression assay showing that repression mediated through HDAC3 recruitment by GAL4 – SMRT, is reduced by mutation of R265. (**d**) Comparison of the effect of various different HDAC inhibitors on the binding of 2-FAM-Ins(1,3,4,5,6)P_5_ to HDAC3:SMRT. (**e**) CD denaturation curves of HDAC3:SMRT showing that InsP_6_ and SAHA both stabilize the complex and have a greater effect when combined. Tm (°C)=melting temperature. Molar ellipticiy was monitored at 222 nm, and melting curves fitted using GraphPad Prism. (**f**) Close-up view of the H4K16Hx (pink sticks) bound in the active site of HDAC1 (electrostatic surface), InsP_6_ (purple) is bound in close proximity to the active site at the interface with MTA1 (green cartoon). Residues involved in binding both InsP_6_ and H4K16Hx are labelled. Error bars indicate±s.e.m. (*n*=3).

The finding that the sites A, B and C are essential for activation of the HDAC3:SMRT complex prompted us to investigate in more detail the mechanism through which inositol phosphates activate class I HDACs. We previously noted that the phosphate at site A in the HDAC3:SMRT structure makes an apparently important contact to the sidechain of R265, and that mutation of this arginine resulted in a significant loss in activity of the enzyme^2^,^3^. One possible explanation for this was that R265A mutant abolished binding of Ins(1,4,5,6)P_4_ to the complex. To test this we measured the binding affinity of 2-FAM-Ins(1,3,4,5,6)P_5_ with the complex. Surprisingly, the dissociation constant was only modestly increased from 0.3 μM to 0.6 μM (Fig. 6a), suggesting that binding of phosphates in sites B and C is the main thermodynamic driver of the interaction and that the interaction of the phosphate in site A with R265 plays a more important role in activation. Fitting with this hypothesis, the mutant complex is only weakly activated by the addition of inositol phosphate (24% of the activation seen for the wild-type complex; Fig. 6b). Furthermore the mutation significantly impairs the ability of the complex to repress transcription of a luciferase reporter gene (Fig. 6c).

Given the importance of R265 in the HDAC3:SMRT:Ins(1,4,5,6)P_4_ complex, it is surprising that the equivalent residue, R270, in the HDAC1:MTA1:InsP6 complex has a different orientation, such that it interacts with the phosphate group in site C. Accordingly, the R270A mutation in the HDAC1:MTA1 complex does not have as pronounced an effect as that seen when R265 is mutated in the HDAC3:SMRT complex, but it still results in a twofold reduction in kcat (Supplementary Fig. 9). We previously observed that the R270Q mutation only modestly impairs the ability of HDAC1 to rescue cells in which both HDAC1 and 2 have been deleted. This mutation does, however, strongly synergise with other mutations in the inositol phosphate-binding pocket^29^. Interestingly, a survey of the available HDAC2 structures (pdb codes: 3MAX, 4LXZ, 4LY1) in the absence of an inositol phosphate ligand, suggests that this arginine is rather mobile and can adopt either the position seen for R265 in the HDAC3:SMRT:Ins(1,4,5,6)P_4_ structure or the position seen in the HDAC1:MTA1:InsP_6_ complex (Supplementary Fig. 10). In either position the backbone trajectory remains unchanged. It seems likely, therefore, that the important feature is that this sidechain becomes immobilized on inositol phosphate binding and that this stabilization is important for activation.

Notably we have also observed a measurable cross-talk between the active site of HDAC3 and the inositol phosphate-binding site. This is illustrated by the finding that binding of hydroxamic acid inhibitors, such as TSA, SAHA and MS-275 (a structurally distinct benzamide inhibitor) at the active site results in a threefold enhancement of the binding of 2-FAM-Ins(1,3,4,5,6)P_5_ to the allosteric site (Fig. 6d). Interestingly, the inhibitor H4K16Hx has a more modest effect on the Kd for 2-FAM-Ins(1,3,4,5,6)P_5_. Those inhibitors that particularly influence 2-FAM-Ins(1,3,4,5,6)P_5_ binding position an aromatic group at the mouth of the active site.

To further investigate the effects of both inhibitors and inositol phosphates, we used a circular dichroism-based thermal-stability assay. The unliganded HDAC3:SMRT complex showed co-operative unfolding at 45 °C, which increased to 51 and 53 °C by SAHA and InsP_6_, respectively. When the thermal denaturation was measured in the presence of both SAHA and InsP_6_ the melting temperature further increased to 62 °C. This indicates that binding to the active site or inositol phosphate results in significant stabilization of the complex (Fig. 6e). Similar effects have also been observed on the stabilization of the HDAC1:MTA1 complex (Supplementary Fig. 11).

## Discussion

Class I HDAC complexes have been implicated in the regulation of a wide range of biological processes from early development and X-chromosome inactivation to metabolism and circadian rhythms^41^,^42^,^43^,^44^,^45^. The finding that these complexes are regulated by inositol phosphates was unexpected and raises the question what is the physiological role of inositol phosphates; under what circumstances do their levels change and which biological processes are consequently regulated? We have not sought to answer these questions in this manuscript. Rather we seek to understand the molecular mechanisms through which inositol phosphates regulate the activity of HDAC complexes. Specifically, we wished to understand what are the important features of inositol phosphates that allow them to activate HDAC co-repressor complexes; what is the relationship between substrate and inositol phosphate binding and what is the mechanism underlying the allosteric activation? We have systematically tested the ability of a range of naturally occurring and synthetic inositol phosphates and their analogues to activate class I HDAC complexes and this has allowed us to elucidate the important stereochemical features of inositol phosphates that are required to allosterically activate class I HDAC complexes. The use of a fluorescently labelled inositol phosphate derivate has allowed the investigation of the direct binding of inositol phosphates to HDACs for the first time. This and other derivatives have effectively demonstrated that inositol phosphate derivatives may have potential use for modulating HDAC co-repressor complexes. The structure of HDAC1 in complex with the novel histone H4 12–18 tail inhibitor peptide has given insight into understanding both substrate recognition and its relationship with inositol phosphate binding. Taken together with kinetic and mutational studies these results allow us to begin to understand the mechanism underlying the allosteric activation by inositol phosphates.

Our investigations into HDAC3:SMRT activation have led us to propose a biological rationale for higher order inositol phosphate signalling to HDAC complexes. Ins(1,4,5)P_3_ is a major precursor for higher inositol phosphates, yet is completely unable to activate HDAC3 since it cannot simultaneously occupy sites A, B and C (Fig. 1a). Addition of a phosphate to Ins(1,4,5)P_3_ at either the 3 or 6 position creates a molecule that can robustly activate HDAC3. Such phosphorylation of Ins(1,4,5)P_3_ is carried out by an inositol phosphate multikinase enzyme (called IPMK or IPK2) (ref. 46, 47). We expect, therefore, that the activity of IPMK is critical for class I HDAC activity *in vivo*, since it is essential for the production of all of the inositol phosphates that are able to activate HDAC complexes. The predominant nuclear localization of IPMK fits well with this biological rationale^48^. Furthermore, the fact that Ins(1,4,5)P_3_, as well as the potent InsP_3_R agonist adenophostin A, are both completely inert in activating HDAC complexes indicates that the signalling role played by higher order inositol phosphates in modulating the activity of class I HDACs is physiologically isolated from the role played by Ins(1,4,5)P_3_ in Ca^2+^ signalling.

Support for the concept that IPMK plays a key role comes from previous findings that both IPMK and its yeast homologue, Arg82p, have been reported to play a role in gene activation^46^,^49^,^50^,^51^,^52^,^53^,^54^. However the role of the kinase activity of Arg82p/IPMK in gene activation is controversial. The kinase activity has been shown to be required for Pho5 transcription and chromatin remodelling^49^, whereas other studies suggest that the kinase activity is not required for the regulation of arginine metabolism in yeast^53^,^54^. IPMK plays a role as a transcriptional co-activator in immediate early gene induction in mice through its interaction with the histone acetyltransferase CBP, though this interaction is not dependent on the kinase activity of IPMK^52^. Interestingly, Xu *et al*.^52^ speculate that the products of the kinase activity of IPMK might be important for repression of gene transcription, whereas the non-catalytic activity stimulates histone acetylation and therefore gene activation.

Despite the uncertainty in the role of the kinase activity of IPMK, it is well-established that all the products of IPMK (higher order inositol phosphates) vary throughout the cell-cycle^55^. This is particularly interesting, since it is established that histone acetylation levels also vary during the cell-cycle^56^, yet the levels of HDAC3 remain constant^57^. Furthermore, deletion of HDAC3 results in loss of the cell-cycle-dependent variation in histone acetylation^57^. If HDAC3 is responsible for cell-cycle regulation of histone acetylation yet its levels do not change, then some other factor (perhaps inositol phosphate levels?) must change.

Despite these suggestive observations it still remains unclear what is the physiologically relevant species that activates HDAC complexes *in vivo*. While Ins(1,4,5,6)P_4_ co-purified with the complex from HEK293 cells, it has become clear that a range of higher order phosphates are able to activate *in vitro*. Importantly inositol phosphate kinases that lie downstream of IPMK and give rise to inositol pyrophosphates have been shown to be important for the activation of the yeast class I HDAC RPD3L and for implementing a stress response in yeast^35^. Indeed, Worley *et al*.^35^ have shown that mutation of residues in the inositol-binding site on the yeast HDAC homologue Rpd3 results in similar effects on gene expression in the environmental stress response as deleting enzymes in the pyrophosphate synthesis pathway (including Arg82). These findings fit well with our observation that certain inositol pyrophosphates and pyrophosphate analogues are able to activate the HDAC3:SMRT complex *in vitro*.

It is striking that there were only very minor structural changes when HDAC1 binds InsP_6_ and the synthetic substrate mimic. The most significant is the rearrangement of the sidechain of D99 that mediates essential interactions with the peptide backbone of the substrate. The importance of this residue in HDAC8 has been noted before^58^. This rearrangement appears to be the result of substrate binding, rather than an allosteric consequence of inositol phosphate binding. Importantly, although the kinetic analysis indicates that substrate binding is strongly influenced by inositol phosphate binding and that catalytic turnover is increased, there is little evidence for a substantive structural change mediating these allosteric effects. This contrasts with the classic dogma of allostery that requires concerted structural changes to mediate thermodynamic or kinetic alterations to an enzyme mechanism^59^. The allosteric mechanism in the HDAC complexes would seem to fit much better with concepts of allostery being the result of changes in entropy (dynamics) following binding at the allosteric site influencing the activity at the active site^60^. This interpretation is supported by recent computational studies which suggest that Ins(1,4,5,6)P_4_ binding induces a change in the dynamic behaviour of the complex in the vicinity of the inositol phosphate and active sites^61^. Furthermore, the thermal denaturation studies reported here show that the binding of inositol phosphate results in a significant stabilization of both the HDAC3:SMRT and HDAC1:MTA1 complexes.

Whilst it is recognized that dynamically driven allostery can occur over a large distance, the fact that the allosteric and active sites are in relatively close proximity provides a likely explanation of how inositol phosphate binding can influence the dynamics of the active site^59^. It is particularly notable that the inositol phosphate and the substrate–peptide interact on either side of a loop involving residues Q26-P29 in HDAC1. Indeed, both the substrate and inositol phosphate form hydrogen bonds with the same peptide bond—Q26-G27 (Fig. 6f).

HDACs have recently shown promise as therapeutic targets to treat a number of different diseases. However, one of the major challenges is that inhibitors of class I HDACs exhibit relatively modest subclass specificity and, furthermore, several different complexes, with diverse biological functions, contain a common catalytic HDAC subunit. The finding that class I HDACs behave quite differently when they are in complex with their cognate co-repressors, provides a new opportunity to develop inhibitors that are specific for individual complexes. Understanding the molecular details of substrate binding, and the allosteric mechanism of activation by inositol phosphates, is likely to be essential for rational drug design.

## Methods

### HDAC3:SMRT protein expression

Full-length HDAC3, and residues 350–480 of SMRT were cloned into pcDNA3 vectors, with a FLAG tag and TEV protease cleavage site in the SMRT construct. To express the complex both constructs were co-transfected into HEK293F cells (Invitrogen). To transfect 300 ml of cells, 150 μg of each construct was diluted in 30 ml PBS (Sigma), vortexed briefly and 1.5 ml of 0.5 mg ml^−1^ 25 kDa branched polyethylenimine (Sigma) added. This mixture was vortexed briefly and incubated for 20 min at room temperature and then added to cells at a final density of 1 × 10^6^ cells per ml. Cells were harvested after 48 h, resuspended in buffer A (100 mM potassium acetate, 50 mM Tris pH 7.5, 5% glycerol, 0.3% Triton X-100, Roche complete protease inhibitor tablet), lysed by sonication and then centrifuged to remove the insoluble material. The cleared supernatant was then incubated with FLAG resin (Sigma) for 1 h at 4 °C. The resin was then washed three times with buffer A, three times with buffer B (300 mM potassium acetate, 50 mM Tris pH 7.5, 5% glycerol), and three times with buffer C (50 mM potassium acetate, 50 mM Tris pH 7.5, 5% glycerol, 0.5 mM TCEP). The resin was incubated with TEV protease overnight at 4 °C in buffer C to elute the protein complex. Final purification of the eluted protein was perfomed by gel filtration in buffer containing 50 mM potassium acetate, 25 mM Tris pH 7.5, 0.5 mM TCEP, on a Superdex S200 column (GE healthcare). The intrinsically bound endogenous Ins(1,4,5,6)P_4_ was removed by treatment with high-ionic strength buffer. 1 μM protein was incubated for 4 h at room temperature in buffer containing 50 mM Tris pH 7.5, 1 M NaCl, 5% glycerol, then dialysed overnight against buffer containing 50 mM Tris pH 7.5, 50 mM NaCl, 5% glycerol. Proteins were then concentrated using an Amicon concentrator and the protein concentration determined by A_280_ in 6 M guanidine hydrochloride.

### HDAC1:MTA1 protein expression and structure determination

Full-length HDAC1 and residues 162–354 of MTA1 were cloned into pcDNA3 vectors, with a FLAG tag and TEV protease cleavage site in the MTA1 construct. Transfections and protein purification were performed as for the HDAC3:SMRT complex with the modification of buffer B to 200 mM potassium acetate, 50 mM Tris pH 7.5, 5% glycerol. Diffracting crystals were obtained by sitting-drop vapour diffusion at 20 °C against wells containing 0.1 M HEPES pH 7.5, 2 M ammonium sulfate and 5% PEG400 by mixing HDAC1:MTA1 (5 mg ml^−1^) and peptide H4K16Hx at a 1:2 molar ratio. Crystals were frozen in mother liquor with the addition of InsP_6_ (225 mM) and 15% glycerol (cryoprotectant). Data were collected at the Diamond synchrotron microfocus beamline I24 with use of the grid-scan tool to centre the crystals. Diffraction data from 4 crystals were processed using Mosflm and combined using Aimless. The structure was solved by molecular replacement with HDAC1:MTA1 (PDB code 4BKX) as a search model using Phaser. The HDAC1:MTA1 structure was built with Coot and refined using Refmac5 (ref. 62). The atomic coordinates for the structure of the HDAC1:MTA1: InsP_6_:H4K16Hx complex have been deposited in the Protein Data Bank under accession code 5ICN.

### Peptide synthesis

Details of the syntheses of peptide H4K16Hx (histone H4 residues 12–18 with hydroxamic acid functionality at K16) and fluorescein-labelled peptide H4K16Ac (FITC-H4 12–18 acetylated at K16) are given in the Supplementary Methods.

### Inositol phosphates and derivatives

Adenophostin A^34^, Ins(1,3,4,5)P_4_ (ref. 63) *scyllo*-InsP_5_ (ref. 31), Bz(1,2,3,4)P_4_ (ref. 32), 1-PA-InsP_5_ (ref. 36), 2-OH-5-PA-InsP_4_ (ref. 37), 2-FAM-Ins(1,3,4,5,6)P_5_ (ref. 38) were synthesized as previously reported. The identities and purities of these compounds were confirmed by ^1^H and ^31^P NMR spectroscopy. Ins(1,3,5,6)P_4_, Ins(3,4,5,6)P_4_, Ins(1,3,4,5,6)P_5_, Ins(1,4,5)P_3_, Ins(1,4,6)P_3_, Ins(1,5,6)P_3_, were purchased from Cayman Chemical Company. InsP_6_ was purchased from Sigma. Details of the new syntheses of Ins(1,4,5,6)P_4_, Ins(4,5,6)P_3_, Ins(1,3,4,6)P_4_, 2-*O*-Bn-Ins(1,4,5,6)P_4_ and 2,3-di-*O*-Bn-Ins(1,4,5,6)P_4_ are given in the Supplementary Methods.

### HDAC assay

HDAC activity of the protein complex was measured using an HDAC assay with a BOC-Lys-AMC substrate. 50 nM HDAC3:SMRT or 50 nM HDAC1:MTA1 was incubated with 100 μM BOC-Lys-AMC substrate in a final volume of 50 μl buffer A (50 mM Tris pH 7.5, 50 mM NaCl, 5% Glycerol), in a black 96-well plate for 30 min at 37 °C. The assay was developed by the addition of 50 μl of developer solution (2 mM TSA, 10 mg ml^−1^ Trypsin, 50 mM Tris pH 7.5, 100 mM NaCl). Fluorescence was measured at 335/460 nm using a Victor X5 plate reader (Perkin Elmer).

To test the ability of inositol phosphates and derivatives to activate the complex, 50 nM HDAC3:SMRT (which had been stripped of its endogenous co-purified inositol phosphate) or 50 nM HDAC1:MTA1 was incubated with inositol phosphate or analogue for 30 min at 37 °C before the HDAC activity was measured. All measurements were performed in triplicate and data analysed using GraphPad Prism (version 6.0, GraphPad Software, Inc). In the case of titrations *K*_d_ values were calculated by nonlinear curve fitting with a one-site binding (hyperbola) model (*Y*=B_max_*X/(*K*_d_+X)).

### Enzyme kinetics

Michaelis–Menten kinetics were determined using a HDAC assay on the Caliper EZ Reader II system (Caliper Life Sciences, http://www.caliperls.com). To assess the effect of inositol phosphate, 20 nM HDAC3:SMRT or 80 nM HDAC1:MTA1 were pre-mixed with InsP_6_ before dilution and addition of fluorescein-labelled H4K16Ac substrate. Reactions were carried out in duplicate in 30 μl reaction volumes, performed at room temperature in 50 mM Tris pH 7.5, 50 mM NaCl, 5% glycerol. Data was analysed using GraphPad Prism (version 6.0).

### Fluorescence anisotropy assays

Fluorescence anisotropy experiments were performed in a black 96-well plate (Corning), each well contained 10 nM 2-FAM-Ins(1,3,4,5,6)P_5_ and an increasing concentration of HDAC3:SMRT protein, in assay buffer (50 mM NaCl, 50 mM Tris pH 7.5, 0.02% Tween-20), final volume of 25 μl. When required, assays were performed in the presence of HDAC inhibitor (20 mM final concentration). Plates were mixed by shaking for 1 s, and measurements taken using a Victor X5 plate reader (Perkin Elmer) at room temperature with an excitation wavelength of 480 nm and an emission wavelength of 535 nm. Experiments were performed in triplicate and data were analysed using GraphPad Prism (version 6.0), *K*_d_ values were calculated by nonlinear curve fitting using a one-site binding (hyperbola) model (Y=Bmax*X/(*K*_d_+X).

Displacement assays were performed essentially as above but with a fixed concentration of protein/2-FAM-Ins(1,3,4,5,6)P_5_ and an increasing concentration of inositol phosphate. The concentration of protein was determined as that which gave 100% bound in the protein titration experiments. The data were analysed and IC_50_ values calculated using GraphPad Prism (version 6.0).

### Reporter gene assays

HEK293T cells were transfected with MH-100-TK-luc reporter plasmid (containing Gal-binding sites), pCMX β-galactosidase, along with GAL-DBD-fused SMRT_350–480_ (wild type and mutants) and untagged HDAC3, using polyethylenimine. Cells were lysed and assayed for reporter expression 48 h after transfection. Luciferase activity was determined using the Luciferase Assay Kit (Biovision) and normalized to the β-galactosidase activity. Measurements were carried out on a Victor X5 plate reader (Perkin Elmer).

### Circular dichroism

A total of 14 μM of HDAC3:SMRT and 3.5 μM of HDAC1:MTA1 were incubated with 200 μM of InsP_6_ or 40 μM SAHA, either individually or in combination. CD was monitored at 222 nm using a Applied Photophysics Chiroscan plus spectropolarimeter, and the sample temperature was increased from 10 °C to 90 °C (1 °C per minute). The data were analysed and melting temperatures were calculated using GraphPad Prism (version 6.0).

### Computational docking studies

Docking studies were carried out with AutoDock4 (ref. 64), utilizing the AutoDockTools 1.5.6 GUI. Non-polar hydrogens and Gasteiger atomic charges were added to the HDAC3:SMRT DAD atomic coordinates (PDB ID: 4A69) in AutoDockTools. The inositol phosphate-binding site was as defined in ref. 2. Probes were calculated at every 0.375 Å grid position of a grid box (box size *x, y, z*=21.406, 50.64, 23.036 Å, respectively), centred upon the inositol ring. The docking of the inositol phosphates was run using the Lamarckian genetic algorithm in AutoDock4. Other parameters were set to the default values.

## Additional information

**How to cite this article**: Watson, P. J. *et al*. Insights into the activation mechanism of class I HDAC complexes by inositol phosphates. *Nat. Commun.* 7:11262 doi: 10.1038/ncomms11262 (2016).

## Supplementary Material

Supplementary Information Supplementary Figures 1-12, Supplementary Discussion, Supplementary Methods and Supplementary References

## Figures and Tables

**Table 1:** Data collection and refinement statistics.

*Data collection*	
Wavelength (Å)	0.96862
Space group	P 3_2_ 2 1
	
Cell dimensions	
a, b, c (Å)	107.8, 107.8, 134.2
α, β, γ (°)	90, 90, 120
Resolution (Å)	93.4–3.3 (3.56–3.3)*
I/σI	2.5 (1.3)*
Completeness (%)	93.1 (92.0)*
Redundancy	2.4 (2.4)*
Rmerge (%)	38.3 (86.4)*
CC_1/2_	0.795 (0.456)*
	
*Refinement*	
*R*_work_/*R*_free_ (%)	25.5/29.9
Reflections	12,263
	
Number of atoms	
Protein	4,326
Zn ions, K ions and IP6	39
Water	0
	
B-factor (Å^2^)	
Protein	34.9
Zn ions, K ions and acetate	29.7
	
R.m.s.d.	
Bond lengths (Å)	0.008
Bond angles (°)	1.228
	
Ramachandran plot	
Favoured (%)	92.3
Allowed (%)	7.7
Outliers (%)	0.0

*The highest resolution shell is shown in parentheses.

## References

[b1] de Ruijter A. J. M., van Gennip A. H., Caron H. N., Kemp S. & van Kuilenburg A. B. P. Histone deacetylases (HDACs): characterization of the classical HDAC family. Biochem. J. 370, 737–749 (2003) . 12429021 10.1042/BJ20021321 PMC1223209

[b2] Watson P. J., Fairall L., Santos G. M. & Schwabe J. W. R. Structure of HDAC3 bound to co-repressor and inositol tetraphosphate. Nature 481, 335–340 (2012) . 22230954 10.1038/nature10728 PMC3272448

[b3] Millard C. J. . Class I HDACs share a common mechanism of regulation by inositol phosphates. Mol. Cell. 51, 57–67 (2013) . 23791785 10.1016/j.molcel.2013.05.020 PMC3710971

[b4] Itoh T. . Structural and functional characterization of a cell cycle associated HDAC1/2 complex reveals the structural basis for complex assembly and nucleosome targeting. Nucleic Acids Res. 43, 2033–2044 (2015) . 25653165 10.1093/nar/gkv068 PMC4344507

[b5] West A. C. & Johnstone R. W. New and emerging HDAC inhibitors for cancer treatment. J. Clin. Invest. 124, 30–39 (2014) . 24382387 10.1172/JCI69738 PMC3871231

[b6] Sumner C. J. . Valproic acid increases SMN levels in spinal muscular atrophy patient cells. Ann. Neurol. 54, 647–654 (2003) . 14595654 10.1002/ana.10743

[b7] Herman D. . Histone deacetylase inhibitors reverse gene silencing in Friedreich's ataxia. Nat. Chem. Biol. 2, 551–558 (2006) . 16921367 10.1038/nchembio815

[b8] Kilgore M. . Inhibitors of class 1 histone deacetylases reverse contextual memory deficits in a mouse model of Alzheimer's disease. Neuropsychopharmacology 35, 870–880 (2009) . 20010553 10.1038/npp.2009.197 PMC3055373

[b9] Shirakawa K., Chavez L., Hakre S., Calvanese V. & Verdin E. Reactivation of latent HIV by histone deacetylase inhibitors. Trends. Microbiol. 21, 1–9 (2013) . 23517573 10.1016/j.tim.2013.02.005 PMC3685471

[b10] Wagner J. M., Hackanson B., Lübbert M. & Jung M. Histone deacetylase (HDAC) inhibitors in recent clinical trials for cancer therapy. Clin. Epigenet. 1, 117–136 (2010) . 10.1007/s13148-010-0012-4 PMC3020651 21258646

[b11] Lee H. Z. . FDA approval: belinostat for the treatment of patients with relapsed or refractory peripheral T-cell lymphoma. Clin. Cancer Res. 21, 1–6 (2015) . 10.1158/1078-0432.CCR-14-3119 25802282

[b12] Laubach J. P., Moreau P., San-Miguel J. F. & Richardson P. G. Panobinostat for the treatment of multiple myeloma. Clin. Cancer Res. 21, 4767–4773 (2015) . 26362997 10.1158/1078-0432.CCR-15-0530

[b13] Micelli C. & Rastelli G. Histone deacetylases: structural determinants of inhibitor selectivity. Drug Discov. Today 20, 1–18 (2015) . 10.1016/j.drudis.2015.01.007 25687212

[b14] Watson P. J., Fairall L. & Schwabe J. W. R. Nuclear hormone receptor co-repressors: structure and function. Mol. Cell. Endocrinol. 348, 440–449 (2012) . 21925568 10.1016/j.mce.2011.08.033 PMC3315023

[b15] Zhang Y. . Analysis of the NuRD subunits reveals a histone deacetylase core complex and a connection with DNA methylation. Genes Dev. 13, 1924–1935 (1999) . 10444591 10.1101/gad.13.15.1924 PMC316920

[b16] Li J. . Both corepressor proteins SMRT and N-CoR exist in large protein complexes containing HDAC3. EMBO J. 19, 4342–4350 (2000) . 10944117 10.1093/emboj/19.16.4342 PMC302030

[b17] Lechner T. . Sds3 (suppressor of defective silencing 3) is an integral component of the yeast Sin3·Rpd3 histone deacetylase complex and is required for histone deacetylase activity. J. Biol. Chem. 275, 40961–40966 (2000) . 11024051 10.1074/jbc.M005730200

[b18] Zhang J., Kalkum M., Chait B. T. & Roeder R. G. The N-CoR-HDAC3 nuclear receptor corepressor complex inhibits the JNK pathway through the integral subunit GPS2. Mol. Cell. 9, 611–623 (2002) . 11931768 10.1016/s1097-2765(02)00468-9

[b19] Guenther M. G., Barak O. & Lazar M. A. The SMRT and N-CoR corepressors are activating cofactors for histone deacetylase 3. Mol. Cell. Biol. 21, 6091–6101 (2001) . 11509652 10.1128/MCB.21.18.6091-6101.2001 PMC87326

[b20] Wen Y. D. . The histone deacetylase-3 complex contains nuclear receptor corepressors. Proc. Natl Acad. Sci. USA 97, 7202–7207 (2000) . 10860984 10.1073/pnas.97.13.7202 PMC16523

[b21] Hu E. . Cloning and characterization of a novel human class I histone deacetylase that functions as a transcription repressor. J. Biol. Chem. 275, 15254–15264 (2000) . 10748112 10.1074/jbc.M908988199

[b22] Lee H., Rezai-Zadeh N. & Seto E. Negative regulation of histone deacetylase 8 activity by cyclic AMP-dependent protein kinase A. Mol. Cell. Biol. 24, 765–773 (2003) . 14701748 10.1128/MCB.24.2.765-773.2004 PMC343812

[b23] Xue Y. . NURD, a novel complex with both ATP-dependent chromatin-remodeling and histone deacetylase activities. Mol. Cell. 2, 851–861 (1998) . 9885572 10.1016/s1097-2765(00)80299-3

[b24] Hassig C. A., Fleischer T. C., Billin A. N., Schreiber S. L. & Ayer D. E. Histone deacetylase activity is required for full transcriptional repression by mSin3A. Cell 89, 341–347 (1997) . 9150133 10.1016/s0092-8674(00)80214-7

[b25] You A., Tong J. K., Grozinger C. M. & Schreiber S. L. CoREST is an integral component of the CoREST-human histone deacetylase complex. Proc. Natl Acad. Sci. USA 98, 1454–1458 (2001) . 11171972 10.1073/pnas.98.4.1454 PMC29278

[b26] Bantscheff M. . Chemoproteomics profiling of HDAC inhibitors reveals selective targeting of HDAC complexes. Nat. Biotechnol. 29, 255–265 (2011) . 21258344 10.1038/nbt.1759

[b27] Guenther M. G. . A core SMRT corepressor complex containing HDAC3 and TBL1, a WD40-repeat protein linked to deafness. Genes Dev. 14, 1048–1057 (2000) . 10809664 PMC316569

[b28] Irvine R. F. & Schell M. J. Back in the water: the return of the inositol phosphates. Nat. Rev. Mol. Cell Biol. 2, 327–338 (2001) . 11331907 10.1038/35073015

[b29] Jamaladdin S. . Histone deacetylase (HDAC) 1 and 2 are essential for accurate cell division and the pluripotency of embryonic stem cells. Proc. Natl Acad. Sci. USA 111, 9840–9845 (2014) . 24958871 10.1073/pnas.1321330111 PMC4103379

[b30] You S. H. . Nuclear receptor co-repressors are required for the histone-deacetylase activity of HDAC3 *in vivo*. Nat. Struct. Mol. Biol. 20, 182–187 (2013) . 23292142 10.1038/nsmb.2476 PMC3565028

[b31] Riley A. M. . *scyllo*-inositol pentakisphosphate as an analogue of *myo*-inositol 1,3,4,5,6-pentakisphosphate: chemical synthesis, physicochemistry and biological applications. Chembiochem 7, 1114–1122 (2006) . 16755629 10.1002/cbic.200600037 PMC1892220

[b32] Mills S. J. . Novel inositol phospholipid headgroup surrogate crystallized in the pleckstrin homology domain of protein kinase Bα. ACS Chem. Biol. 2, 242–246 (2007) . 17432822 10.1021/cb700019r

[b33] Takahashi S., Kinoshita T. & Takahashi M. Adenophostins A and B: potent agonists of inositol-1,4,5-trisphosphate receptor produced by Penicillium brevicompactum. Structure elucidation. J. Antibiot. 47, 95–100 (1994) . 8119867 10.7164/antibiotics.47.95

[b34] Marwood R. D., Correa V., Taylor C. W. & Potter B. V. L. Synthesis of adenophostin A. Tetrahedron: Asymmetry 11, 397–403 (2000) .

[b35] Worley J., Luo X. & Capaldi A. P. Inositol pyrophosphates regulate cell growth and the environmental stress response by activating the HDAC Rpd3L. Cell. Rep. 3, 1476–1482 (2013) . 23643537 10.1016/j.celrep.2013.03.043 PMC3672359

[b36] Pulloor N. K. . Human genome-wide RNAi screen identifies an essential role for inositol pyrophosphates in Type-I interferon response. PLoS Pathog. 10, e1003981 (2014) . 24586175 10.1371/journal.ppat.1003981 PMC3937324

[b37] Riley A. M., Wang H., Weaver J. D., Shears S. B. & Potter B. V. L. First synthetic analogues of diphosphoinositol polyphosphates: interaction with PP-InsP_5_ kinase. Chem. Commun. 48, 11292–11294 (2012) . 10.1039/c2cc36044f PMC3923271 23032903

[b38] Riley A. M., Windhorst S., Lin H.-Y. & Potter B. V. L. Cellular internalisation of an inositol phosphate visualised by using fluorescent InsP_5_. ChemBioChem 15, 57–67 (2014) . 24311195 10.1002/cbic.201300583 PMC4159588

[b39] Mwakwari S. C., Patil V., Guerrant W. & Oyelere A. K. Macrocyclic histone deacetylase inhibitors. Curr. Top. Med. Chem. 10, 1423–1440 (2010) . 20536416 10.2174/156802610792232079 PMC3144151

[b40] Hu E. . Identification of novel isoform-selective inhibitors within class I histone deacetylases. J. Pharmacol. Exp. Ther. 307, 720–728 (2003) . 12975486 10.1124/jpet.103.055541

[b41] Knutson S. K. . Liver-specific deletion of histone deacetylase 3 disrupts metabolic transcriptional networks. EMBO J. 27, 1017–1028 (2008) . 18354499 10.1038/emboj.2008.51 PMC2323257

[b42] Feng D. . A circadian rhythm orchestrated by histone deacetylase 3 controls hepatic lipid metabolism. Science 331, 1315–1319 (2011) . 21393543 10.1126/science.1198125 PMC3389392

[b43] McHugh C. A. . The Xist lncRNA interacts directly with SHARP to silence transcription through HDAC3. Nature 521, 232–236 (2015) . 25915022 10.1038/nature14443 PMC4516396

[b44] Casas-Delucchi C. S. . Histone acetylation controls the inactive X chromosome replication dynamics. Nat. Commun. 2, 222–11 (2011) . 21364561 10.1038/ncomms1218 PMC3072080

[b45] Sun Z. . Hepatic Hdac3 promotes gluconeogenesis by repressing lipid synthesis and sequestration. Nat. Med. 18, 934–942 (2012) . 22561686 10.1038/nm.2744 PMC3411870

[b46] Odom A. R. A role for nuclear inositol 1,4,5-trisphosphate kinase in transcriptional control. Science 287, 2026–2029 (2000) . 10720331 10.1126/science.287.5460.2026

[b47] Saiardi A. . Mammalian inositol polyphosphate multikinase synthesizes inositol 1,4,5-trisphosphate and an inositol pyrophosphate. Proc. Natl Acad. Sci. USA 98, 2306–2311 (2001) . 11226235 10.1073/pnas.041614598 PMC30134

[b48] Resnick A. C. & Saiardi A. Inositol polyphosphate multikinase: metabolic architect of nuclear inositides. Front. Biosci. J. Virtual 13, 856–866 (2007) . 10.2741/2726 17981594

[b49] Steger D. J., Haswell E. S., Miller A. L., Wente S. R. & O'Shea E. K. Regulation of chromatin remodeling by inositol polyphosphates. Science 299, 114–116 (2003) . 12434012 10.1126/science.1078062 PMC1458531

[b50] El Alami M., Messenguy F., Scherens B. & Dubois E. Arg82p is a bifunctional protein whose inositol polyphosphate kinase activity is essential for nitrogen and PHO gene expression but not for Mcm1p chaperoning in yeast. Mol. Microbiol. 49, 457–468 (2003) . 12828642 10.1046/j.1365-2958.2003.03562.x

[b51] Shen X., Xiao H., Ranallo R., Wu W.-H. & Wu C. Modulation of ATP-dependent chromatin-remodeling complexes by inositol polyphosphates. Science 299, 112–114 (2003) . 12434013 10.1126/science.1078068

[b52] Xu R. . Inositol polyphosphate multikinase is a transcriptional coactivator required for immediate early gene induction. Proc. Natl Acad. Sci. USA 110, 16181–16186 (2013) . 24043835 10.1073/pnas.1315551110 PMC3791727

[b53] Bosch D. & Saiardi A. Arginine transcriptional response does not require inositol phosphate synthesis. J. Biol. Chem. 287, 38347–38355 (2012) . 22992733 10.1074/jbc.M112.384255 PMC3488103

[b54] Dubois E., Dewaste V., Erneux C. & Messenguy F. Inositol polyphosphate kinase activity of Arg82/ArgRIII is not required for the regulation of the arginine metabolism in yeast. FEBS Lett. 486, 300–304 (2000) . 11119723 10.1016/s0014-5793(00)02318-8

[b55] Barker C. J., Wright J., Hughes P. J., Kirk C. J. & Michell R. H. Complex changes in cellular inositol phosphate complement accompany transit through the cell cycle. Biochem. J. 380, 465–473 (2004) . 14992690 10.1042/BJ20031872 PMC1224188

[b56] Unnikrishnan A., Gafken P. R. & Tsukiyama T. Dynamic changes in histone acetylation regulate origins of DNA replication. Nat. Struct. Mol. Biol. 17, 430–437 (2010) . 20228802 10.1038/nsmb.1780 PMC3060656

[b57] Bhaskara S. . Hdac3 is essential for the maintenance of chromatin structure and genome stability. Cancer Cell 18, 436–447 (2010) . 21075309 10.1016/j.ccr.2010.10.022 PMC3004468

[b58] Vannini A. . Substrate binding to histone deacetylases as shown by the crystal structure of the HDAC8-substrate complex. EMBO. Rep. 8, 879–884 (2007) . 17721440 10.1038/sj.embor.7401047 PMC1973954

[b59] Tsai C. J., del Sol A. & Nussinov R. Allostery: absence of a change in shape does not imply that allostery is not at play. J. Mol. Biol. 378, 1–11 (2008) . 18353365 10.1016/j.jmb.2008.02.034 PMC2684958

[b60] Cooper A. & Dryden D. T. F. Allostery without conformational change. Eur. Biophys. J. 11, 103–109 (1984) . 6544679 10.1007/BF00276625

[b61] Arrar M., Turnham R., Pierce L., de Oliveira C. A. F. & McCammon J. A. Structural insight into the separate roles of inositol tetraphosphate and deacetylase-activating domain in activation of histone deacetylase 3. Protein Sci. 22, 83–92 (2013) . 23139175 10.1002/pro.2190 PMC3575863

[b62] Winn M. D. . Overview of the CCP4 suite and current developments. Acta. Crystallogr. D. Biol. Crystallogr. 67, 235–242 (2011) . 21460441 10.1107/S0907444910045749 PMC3069738

[b63] Riley A. M., Mahon M. F. & Potter B. V. L. Rapid synthesis of the enantiomers of *myo*-inositol-1,3,4,5-tetrakisphosphate by direct chiral desymmetrization of *myo*-inositol orthoformate. Angewandte Chemie 36, 1472–1474 (1997) .

[b64] Pospisil P. . Computational and biological evaluation of quinazolinone prodrug for targeting pancreatic cancer. Chem. Biol. Drug Des. 79, 926–934 (2012) . 22304734 10.1111/j.1747-0285.2012.01350.x

