# Supplementary material for: Insights into the activation mechanism of class I HDAC complexes by inositol phosphates
Source: Nat Commun. 2016 Apr 25;7:11262. doi: 10.1038/ncomms11262 (PMC4848466; doi:10.1038/ncomms11262)
Supplement: Supplementary Information — Supplementary Figures 1-12, Supplementary Discussion, Supplementary Methods and Supplementary References [file ncomms11262-s1.pdf]

Supplementary Figures

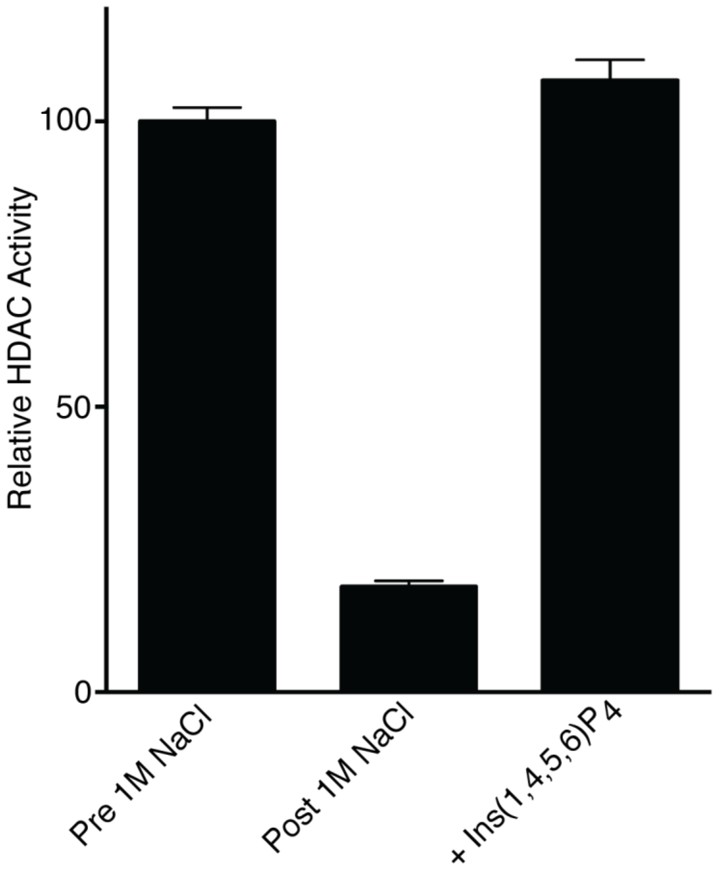

Supplementary Figure 1: Removal of endogenous Ins(1,4,5,6)P_4 from the HDAC3:SMRT complex

Endogenous Ins(1,4,5,6)P_4 can be removed from the HDAC3:SMRT complex by treatment with high ionic strength buffer (e.g. 1M NaCl) resulting in a complex which can be readily activated by exogenous Ins(1,4,5,6)P_4. Error bars indicate ± s.e.m. (n=3)

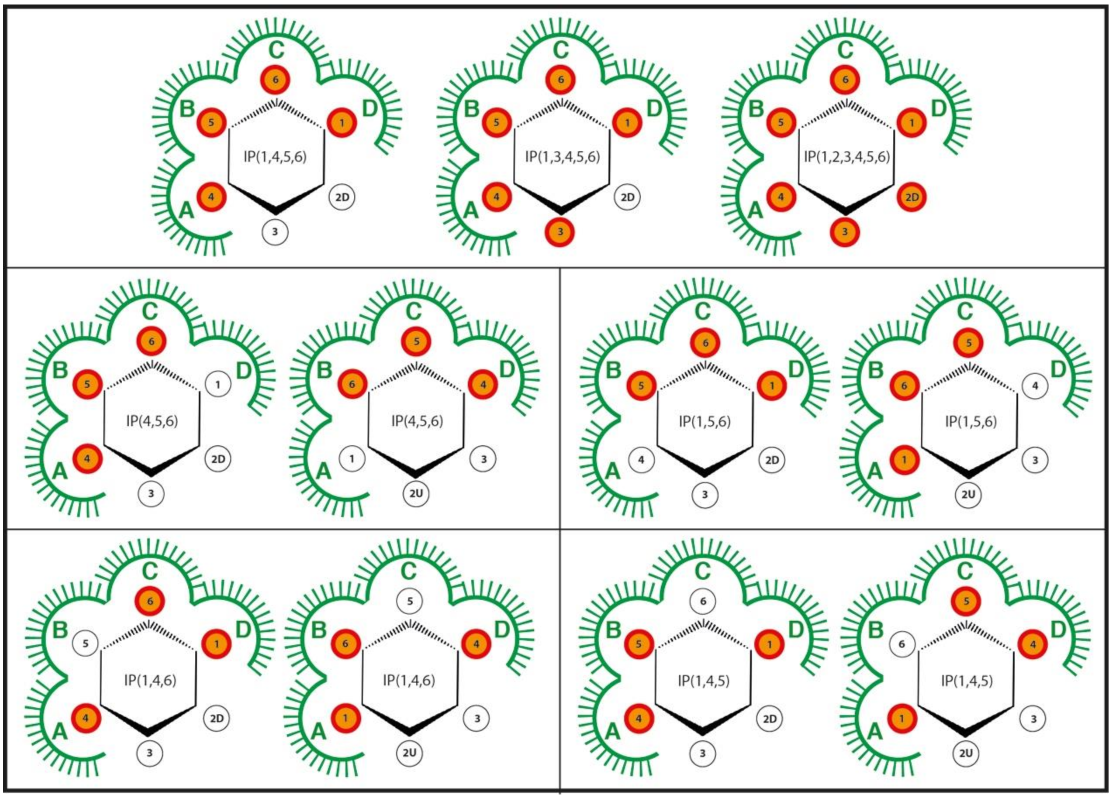

Supplementary Figure 2: The likely modes of binding of inositol trisphosphates to the HDAC3:SMRT complex

Schematic representation of the inositol phosphate binding pocket with the phosphate sub-sites designated A, B, C, and D as indicated. “2D” and “2U” indicate that the axial hydroxyl group at the second position of the inositol ring is pointing down or up respectively. The observed or expected binding modes of Ins(1,4,5,6)P4, Ins(1,3,4,5,6)P5, and Ins(1,2,3,4,5,6)P6 are shown in the top panel. The modes of binding for inositol trisphosphates, which preserve the correct orientation of the inositol chair conformation and fulfill 3 of the 4 phosphate sub-sites, are shown in the lower panels. Ins(4,5,6)P_3, Ins(1,5,6)P_3 are able to fulfill both sites B and C (along with site A or D) correlating with their ability to activate HDAC3.

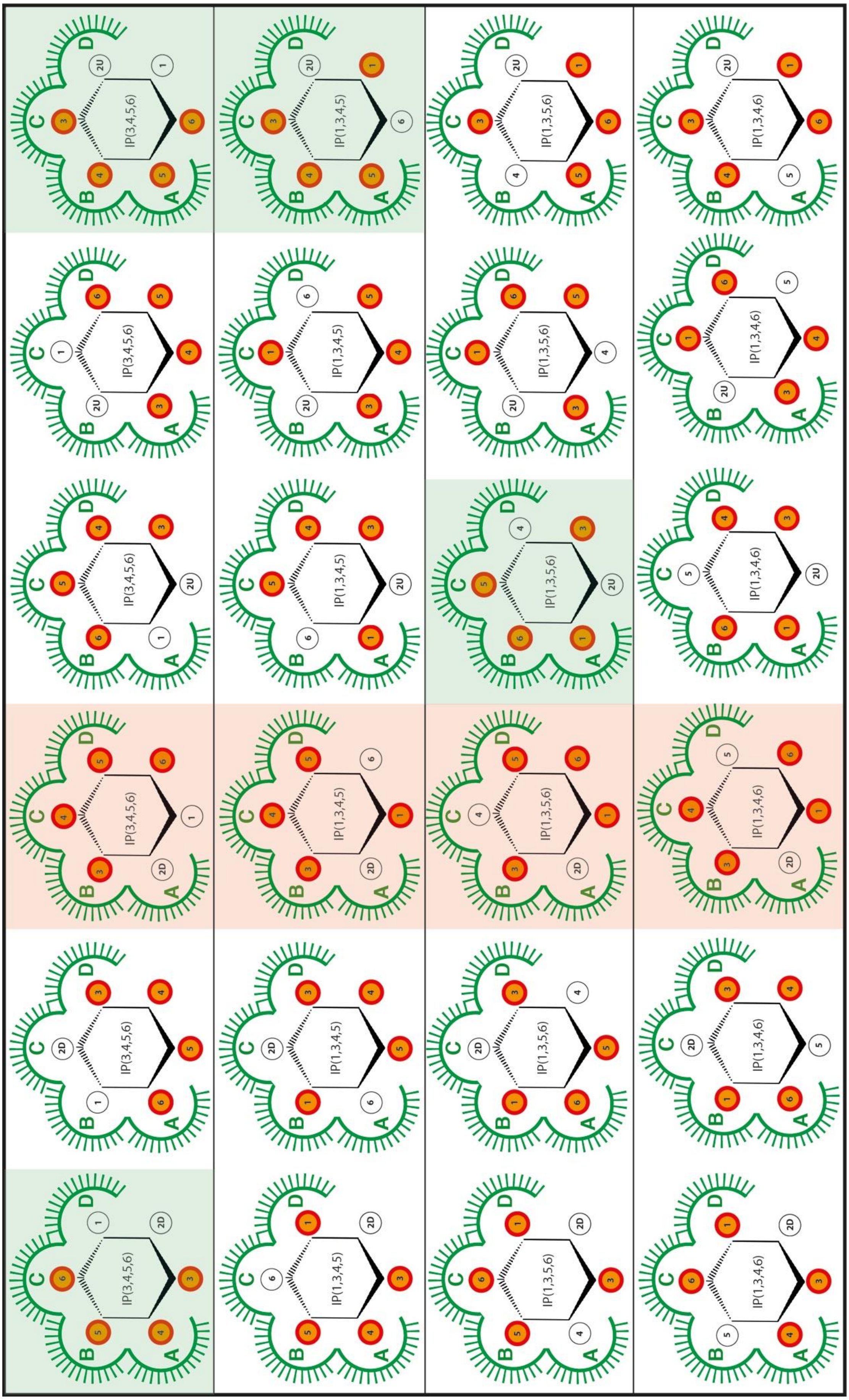
IP(3,4,5,6)
IP(1,3,4,5)
IP(1,3,5,6)
IP(1,3,4,6)
A
B
C
D

Supplementary Figure 3: The possible modes of binding of the inositol tetrakisphosphates

There are six possible binding modes for inositol phosphates that retain the “correct” orientation of the chair confirmation with respect to the binding site. One of these binding modes (shaded red) is likely to be strongly disfavored since it positions the axial hydroxyl such that it would result in a steric clash with the protein surface. Binding modes that fulfill sub-sites A, B & C, and are likely to be most active, are shaded green.

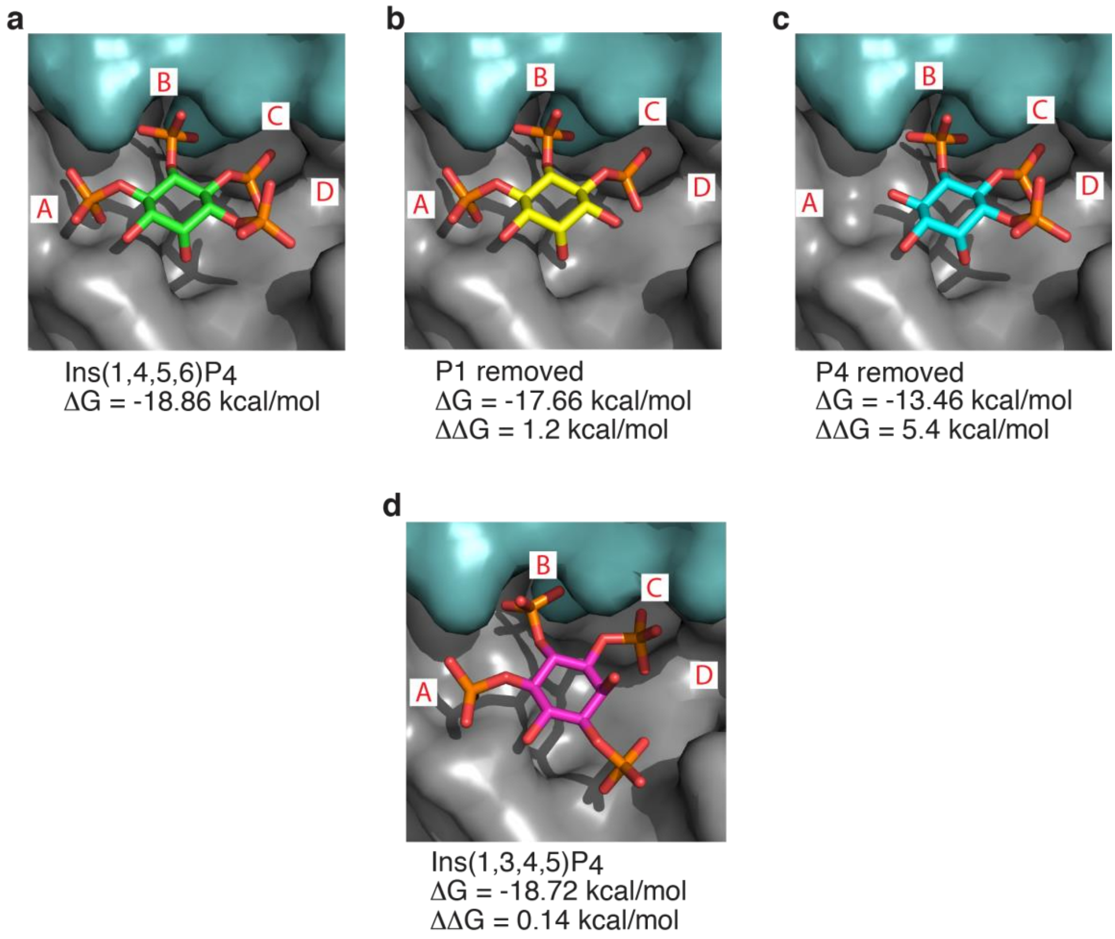

Supplementary Figure 4: Docking studies of inositol phosphates into HDADC3:SMRT –DAD structure.

A) Docked position of Ins(1,4,5,6)P_4 **B/C)** Docked position of Ins(1,4,5,6)P_4 where the phosphate groups in position 1 & 4 have been deleted respectively. **D)** Docked position of Ins(1,3,4,5,)P_4. ΔΔG values are calculated with respect to the free energy of binding of Ins(1,4,5,6)P_4 to HDAC3:SMRT-DAD.

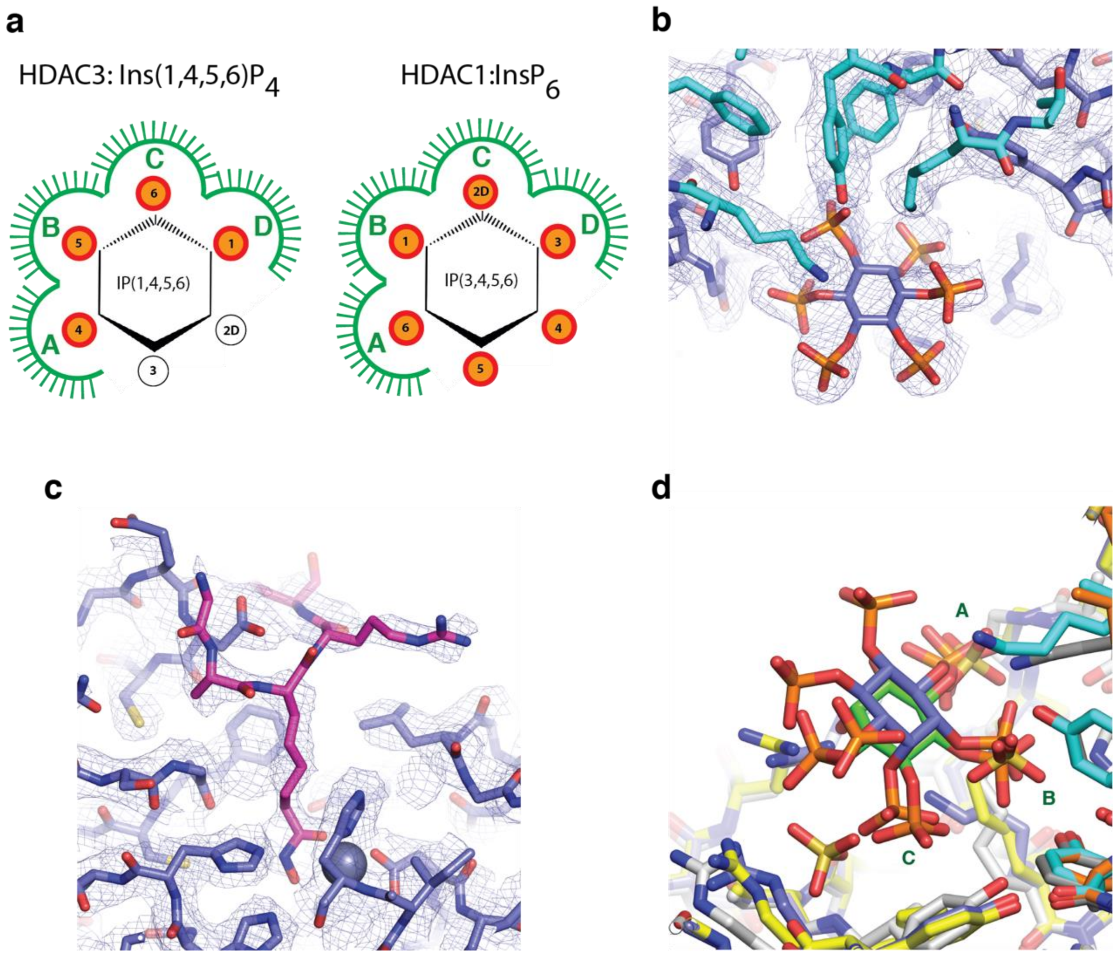

Supplementary Figure 5: Position of inositol phosphates and the novel peptide inhibitor in the structure of HDAC1:MTA1

A) Schematic representation of inositol phosphates in the HDAC3:SMRT and HDAC1:MTA1 binding pockets **B)** Electron density map of the inositol phosphate (orange and purple) positioned between HDAC1 (purple) and MTA1 (cyan). **C)** Electron density map of H4K16Hx (pink) bound to HDAC1 with the active site zinc is shown as a grey sphere. Both $2F_o$-F_c maps are contoured at 1.0σ. **D)** Occupation of the inositol phosphate-binding site at the interface between HDAC and corepressor proteins. Sites A, B and C are occupied by $InsP_6$ (purple) in the HDAC1:MTA1 structure, by Ins(1,4,5,6)P_4 (green) in the HDAC3:SMRT structure and sites A and B are occupied by sulfate molecules in the HDAC1:MTA1 apo structure.

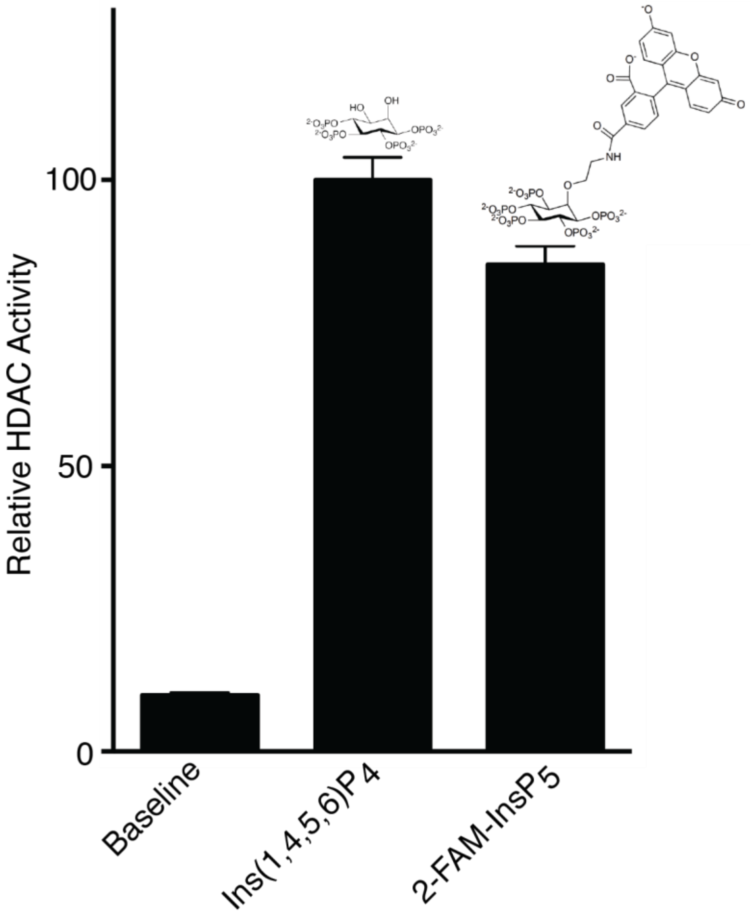

Supplementary Figure 6: Activation of HDAC3:SMRT by 2-FAM-InsP$_5$

The ability of 2-FAM-InsP$_5$ to activate HDAC3:SMRT is compared with Ins(1,4,5,6)P$_4$. HDAC activity was measured in the presence of 200 □mM inositol phosphate / 2-FAM-InsP$_5$. HDAC activity is expressed relative to the maximal Ins(1,4,5,6)P$_4$ activity. Error bars indicate ± s.e.m. (*n*=3)

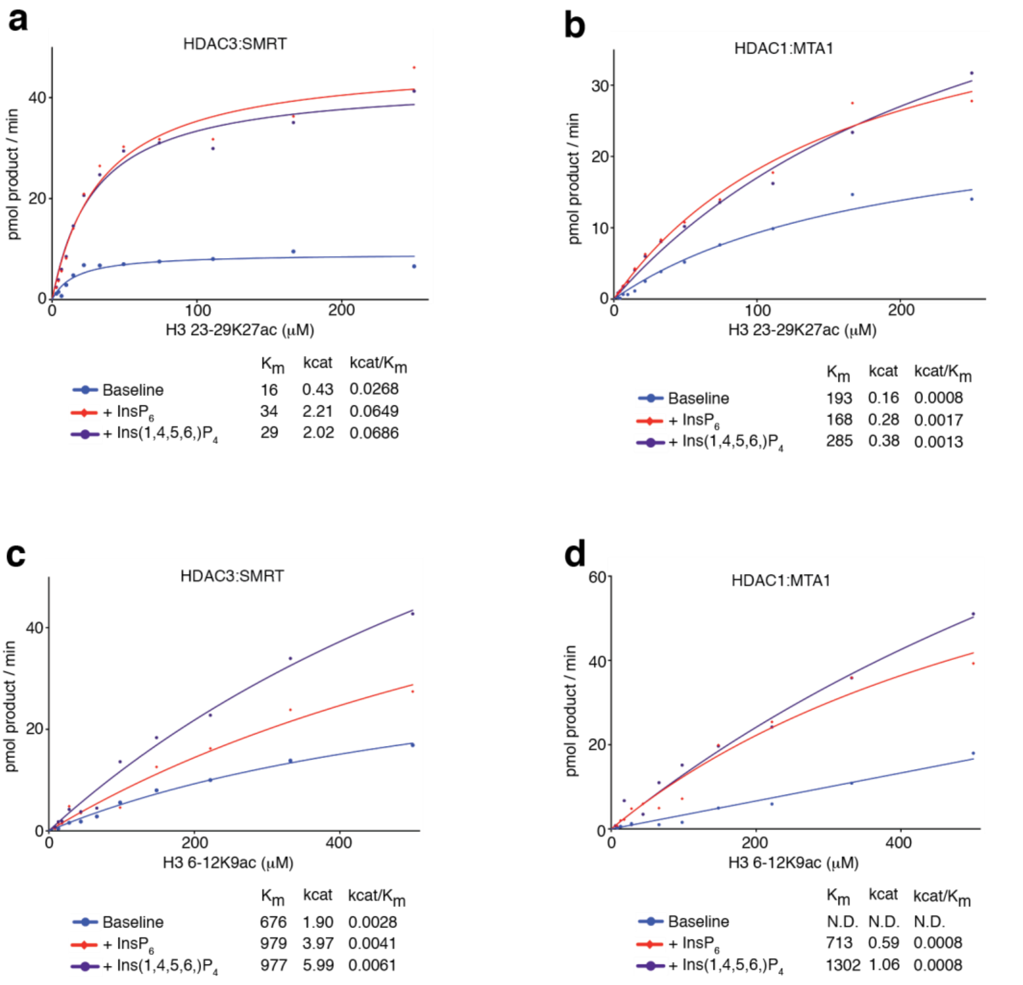

Supplementary Figure 7: The effect of inositol phosphate on kinetic parameters

Determination of enzyme kinetic parameters in the presence and absence of $InsP_6$ and Ins(1,4,5,6)P_4 for the HDAC3:SMRT and HDAC1:MTA1 complexes with H3 23-29K27ac and H3 6-12K9ac peptides.

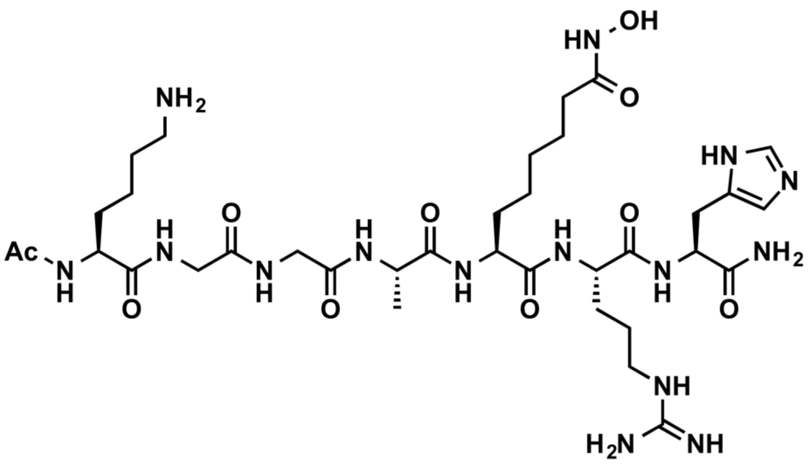

Supplementary Figure 8: H4K16Hx – A novel peptide based inhibitor

Schematic representation of the H4K16Hx peptide based inhibitor, which comprises residues 12-18 of histone H4 with K16 being replaced by a hydroxamic acid functionality

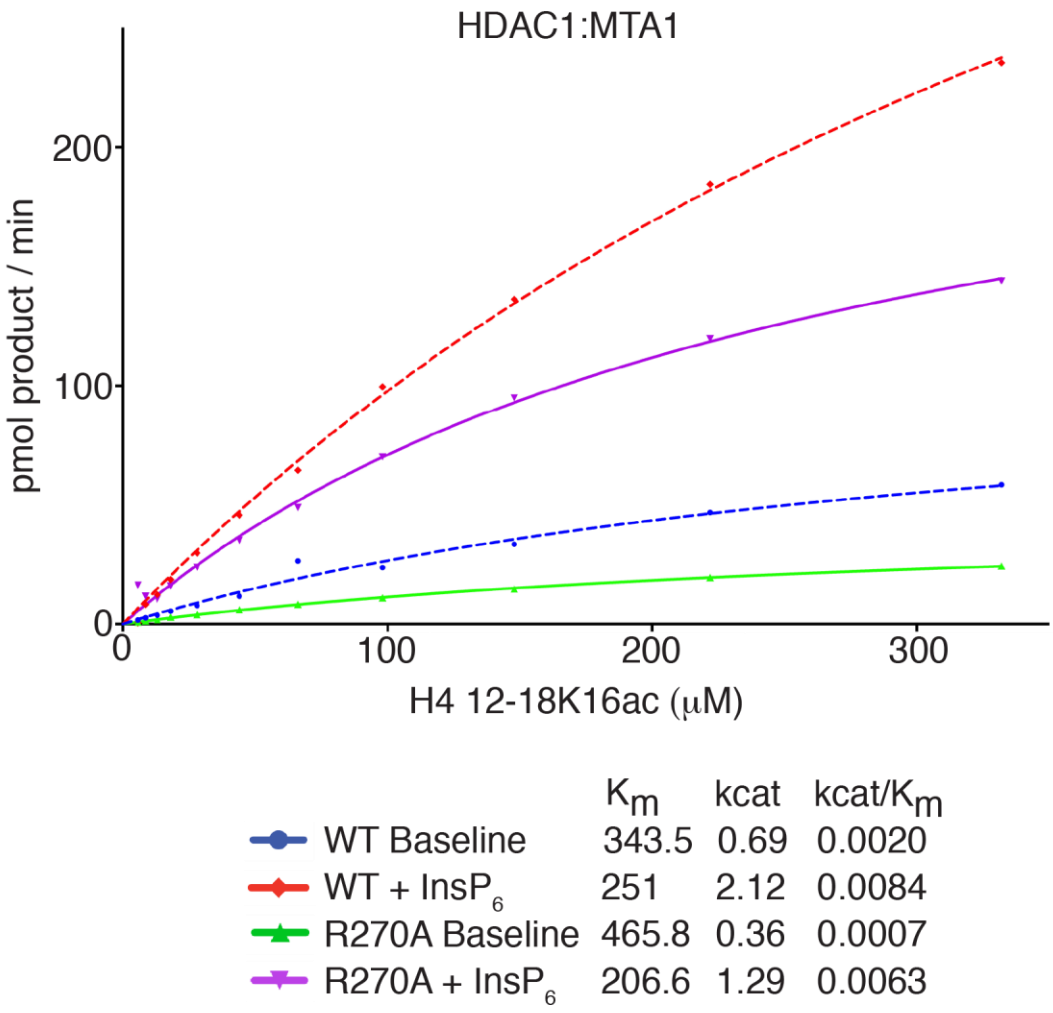

Supplementary Figure 9: The effect of the R270A mutation in HDAC1 on the kinetic parameters

Measurement of Km and kcat in the presence and absence of $InsP_6$, for HDAC1 (R270A):MTA1. Data for the wild type HDAC1:MTA1 is also shown for comparison.

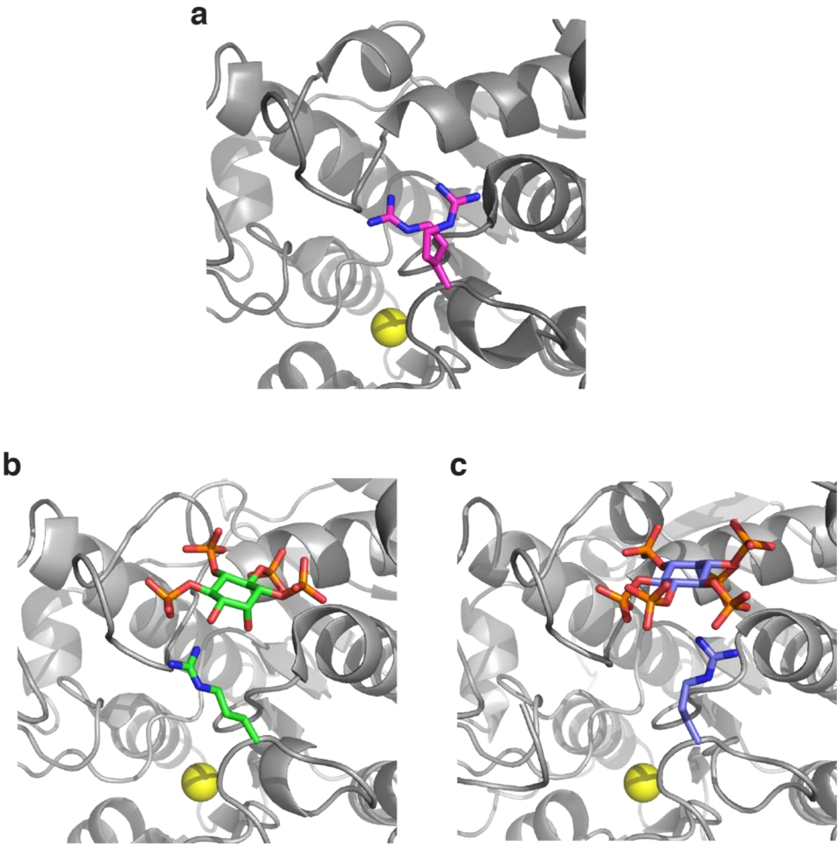

Supplementary Figure 10: Relative mobility of arginine 275 in HDAC2

A) Arginine 275 in HDAC2 (pink sticks) is found in two conformations in the available crystal structures (pdb codes: 3MAX, 4LXZ, 4LY1). **B/C)** R265 of HDAC3 and Ins(1,4,5,6)P_4 from the HDAC3:SMRT structure (green sticks) and R270 of HDAC1 and $InsP_6$ (purple sticks) from HDAC1:MTA1 structure are shown with R275 from HDAC2 (pink sticks) for comparison. Catalytic zinc atoms are shown in yellow.

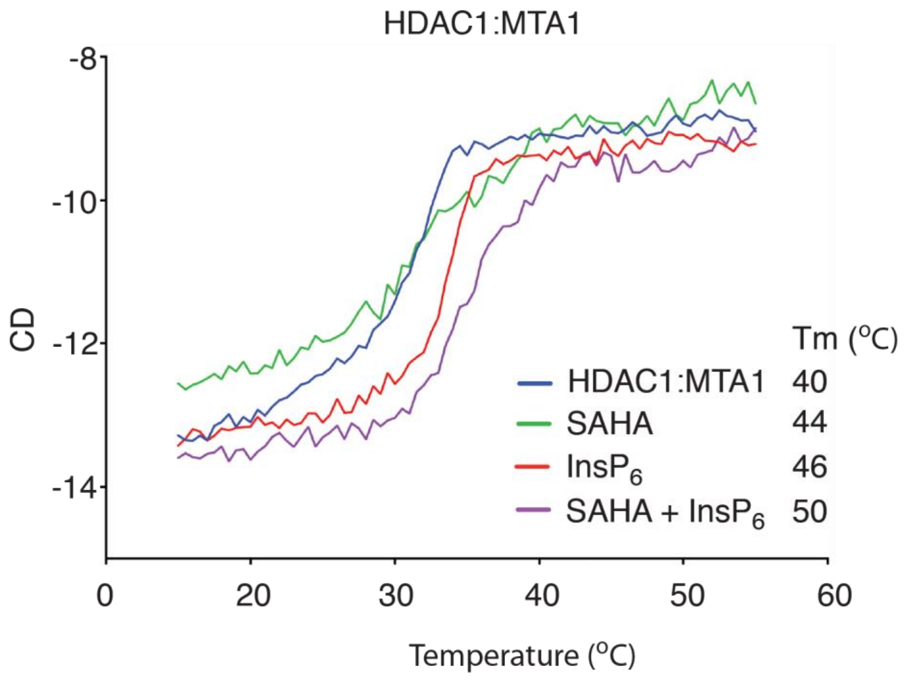

Supplementary Figure 11: The effect of inhibitor and inositol phosphate on the thermal stability of the HDAC1:MTA1 complex

CD denaturation curves of the HDAC1:MTA1 complex showing that InsP6 and SAHA both stabilize the complex and have a greater effect when combined. Tm (^{o}C) = melting temperature. Molar ellipticiy was monitored at 222 nm, and melting curves fitted using GraphPad Prism.

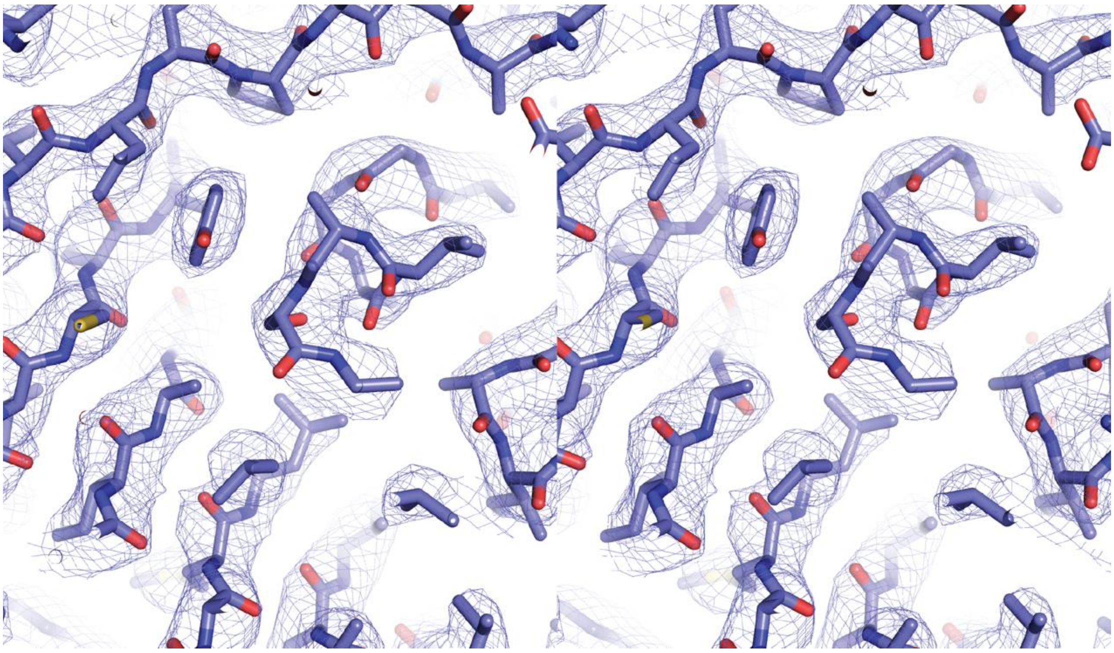

Supplementary Figure 12: Stereo image of a representative portion of electron density for the HDAC1:MTA1:K16Hx:InsP$_6$ structure

Map shown is 2F$_o$-F$_c$ map contoured at 1.0σ.

Supplementary Discussion

Stereochemistry of HDAC activation by inositol phosphates

To establish whether all four phosphates are required for activation of the HDAC3 complex we tested four inositol trisphosphates. Remarkably, while Ins(4,5,6)P_3 and Ins(1,5,6)P_3 were able to activate to 85% and 60% respectively, the Ca^{2+}-releasing second messenger Ins(1,4,5)P_3 and its regioisomer, Ins(1,4,6)P_3 were completely inert (**Figure 1D**). At first sight this would appear to suggest that phosphates on both the 5 and 6 positions are essential for HDAC3 activation and those in the 1 and 4 position are less important. However, the interpretation is complicated because there are six potential binding-modes for a substituted inositol ring that maintain the same orientation of the cyclitol chair conformation relative to the binding pocket. These binding modes differ in the position and orientation of the axial hydroxyl group at position 2. Of the six potential binding modes, one is likely sterically excluded due to a significant clash between the axial hydroxyl and residues in HDAC3. A further complication is that it is likely that some of the inositol phosphates may bind in multiple poses which could differ in their ability to activate the complex. Despite these complications we suggest that it is possible to draw tentative conclusions regarding the stereochemical requirements for activation.

Since the orientation of the inositol ring is not fixed, we will refer to the phosphate positions in the binding pocket as sites A, B, C & D (**Figure 1A**). When the allowable alternative binding modes are considered, it is clear that both Ins(1,5,6)P_3 and Ins(4,5,6)P_3 can occupy either phosphate binding sites A, B & C or B, C & D. In contrast, Ins(1,4,5)P_3 and Ins(1,4,6)P_3 occupy either sites A, B & D or A, C & D (**Supplementary Figure 2**). We can therefore conclude from these experiments that it is essential that both phosphate sites B and C are occupied for HDAC3 activity. The experiments with the inositol trisphosphates do not rule out the possibility that a phosphate may also be required in either site A or site D, but clearly it is not necessary to occupy both.

To explore the requirements further we tested the ability of a number of naturally occurring and synthetic inositol tetrakisphosphates to activate HDAC3. Of these, Ins(1,3,4,6)P_4[1], is unable to activate HDAC3 (**Figure 1D**). Importantly, in one binding mode, Ins(1,3,4,6)P_4 is able to occupy both sites B and C (with sites A and D unoccupied) (**Supplementary Figure 3**), yet this is insufficient to activate HDAC3. We can conclude therefore that in addition to occupying sites B and C, either site A or site D *must* be occupied.

In contrast to Ins(1,3,4,6)P_4, the other tetrakisphosphates tested are all able to achieve 75% or greater activation of the complex compared with Ins(1,4,5,6)P_4 (**Figure 1D**). It is apparent that both Ins(3,4,5,6)P_4 and Ins(1,3,5,6)P_4 have potential binding modes in which both sites B and C and either A or D are occupied. In contrast, for Ins(1,3,4,5)P_4 when sites B and C are occupied, only site A can be occupied. We can therefore conclude that occupancy of sites A, B and C is sufficient to activate HDAC3. The requirement for site A to be occupied fits well with our understanding of the structure, since the phosphate at this site forms a salt bridge with Arginine 265 which has been shown to be essential for activation of the enzyme[2]. Indeed, Arginine 265 is a key residue proximal to the active site of the enzyme.

Supplementary Methods

General Chemistry Methods

Chemicals were purchased from Sigma-Aldrich or Alfa Aesar and used without further purification. Anhydrous solvents from Sigma-Aldrich were used without further treatment. TLC was performed on pre-coated plates (Merck Aluminum sheets silica 60 F_{254}, art No. 5554). Chromatograms were visualised under UV light and by dipping plates into phosphomolybdic acid in EtOH, followed by heating. Proton ^{1}H NMR spectra were recorded on Bruker Avance III (400 MHz and 500 MHz) spectrometers. Proton chemical shifts are reported in ppm (δ) relative to internal tetramethylsilane (TMS, δ 0.0 ppm) or with the solvent reference relative to TMS employed as the internal standard (D_2O: 4.79 ppm). The following abbreviations are used to describe resonances: br, broad; s, singlet; d, doublet; dd, double doublet; q, quartet; m, multiplet; t, triplet. ^{13}C spectra were recorded on Bruker Avance III (100 MHz and 126 MHz) spectrometers with complete proton decoupling. Carbon chemical shifts are reported in ppm (δ) relative to TMS with the respective solvent resonance as the internal standard (CD_3OD: 49.15 ppm). ^{31}P NMR NMR spectra were recorded on a Bruker Avance III (162 MHz) spectrometer with complete proton decoupling. Phosphorus chemical shifts are reported in ppm (δ) relative to an 85% H_3PO_4 external standard (H_3PO_4, 0.0 ppm). Melting points were determined using a Stanford Research Systems Optimelt MPA100 automated melting point system and are uncorrected. Mass spectra were recorded at the University of Bath on a Bruker MicroTOF instrument. Flash column chromatography was performed on an ISCO CombiFlash Rf automated flash chromatography system using RediSep Rf disposable flash columns.

Synthesis of *myo*-inositol 4,5,6-trisphosphate (1)

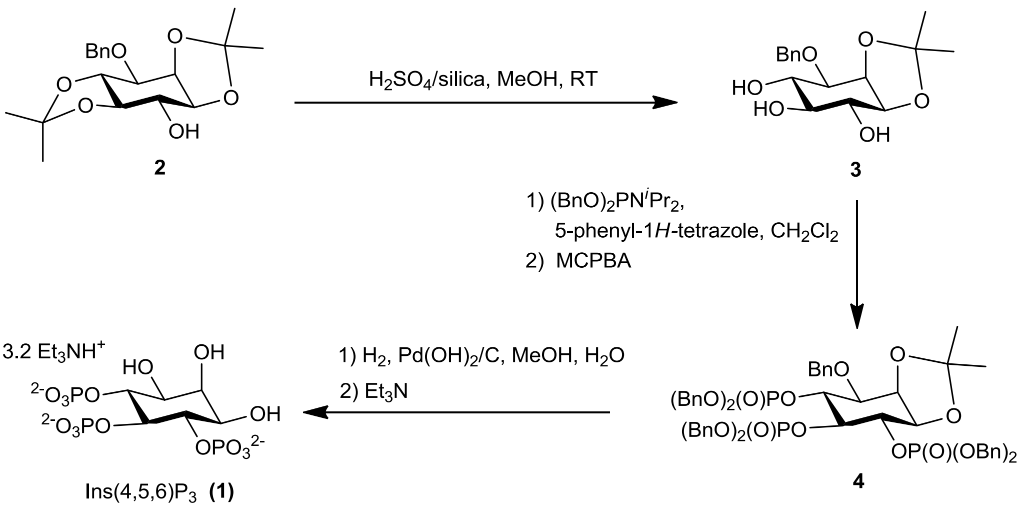

myo-Inositol 4,5,6-trisphosphate[3-5] was synthesised from DL-1,2:4,5-di-*O*-isopropylidene-3-*O*-benzyl-*myo*-inositol (**2**) [6]. The *trans*-isopropylidene group protecting O-4 and O-5 was removed using silica-supported H_2SO_4[7], and phosphitylation of the resulting triol (**3**) followed by oxidation with MCPBA gave the fully protected intermediate **4**. Hydrogenolysis over $Pd(OH)_2$ on carbon removed all benzyl protecting groups from **4**, and the remaining *cis*-isopropylidene group was cleaved *in situ,* with the newly generated acidic phosphate monoesters providing the required acid catalysis.

DL-3-*O*-benzyl-1,2-*O*-isopropylidene-*myo*-inositol (3)

To a stirred solution of DL-3-*O*-benzyl-1,2:4,5-di-*O*-isopropylidene-*myo*-inositol (**2**) [6] (326 mg, 0.930 mmol) in methanol (8 mL) at room temperature was added H_2SO_4/silica[8] (8 mg). After 30 min, TLC (ethyl acetate) showed that the starting material (R_f 0.80) had been converted into two polar products (R_f 0.20 and R_f 0). Solid $NaHCO_3$ was added to quench the reaction. The resulting suspension was filtered, and the filtrate was concentrated to give an oil, which was purified by flash chromatography (ethyl acetate) to give **3** as a white solid (117 mg, 0.378 mmole, 41%, not optimised): mp: 148.5–150 ^oC (from ethyl acetate:light petroleum);. TLC (ethyl acetate): R_f = 0.20; ^1H NMR ($CDCl_3$, 500 MHz) δ 7.40–7.28 (m, 5H), 4.75 (AB system, J_{AB} = 12.2 Hz, 2H), 4.25 (t, J = 4.5 Hz, 1H), 3.94 (br s, OH, 1H), 3.89–3.83 (m, 2H), 3.65–3.61 (m, 2H), 3.49 (dd, J = 9.5, 4.1 Hz, 1H), 3.46 (br s, OH, 1H), 3.24 (t, J = 9.5 Hz, 1H), 1.51 (s, 3H), 1.31 (s, 3H); ^{13}C NMR ($CDCl_3$, 100 MHz) δ 137.77, 128.57, 128.17,

128.12, 110.15, 78.82, 77.34 (buried by $CDCl_3$ peaks, signal obtained from DEPT-135 experiment), 75.03, 73.59, 73.18, 72.62, 71.76, 28.35, 26.00; HRMS (*m*/*z*) $[M+H]^+$ cacld for $C_{16}H_{22}O_6$, 311.1489; found 311.1486.

DL-3-*O*-benzyl-1,2-*O*-isopropylidene-*myo*-inositol 4,5,6-tris(dibenzylphosphate) (4)

To a solution of of triol **3** (90 mg, 0.29 mmol) in dry CH_2Cl_2 (3 mL) was added 5-phenyl-1*H*-tetrazole (190 mg 1.30 mmol). The suspension was stirred under N_2 and bis(benzyloxy)diisopropylaminophosphine (0.4 mL, 1.2 mmol) was added. The suspension became clear within 5 min. The resulting solution was stirred at room temperature for 1.5 h and then cooled to –78 ºC, before MCPBA (70%, 428 mg, 1.74 mmol) was added in portions over 1 min. The mixture was allowed to warm to room temperature and then diluted with EtOAc (30 mL). The clear solution was washed with 10% aq. sodium sulfite solution (3 × 30 mL), dried over $MgSO_4$ and concentrated. The residue was purified by flash chromatography eluting with 0% to 100% EtOAc in CH_2Cl_2 to give **4** as a colourless oil (271 mg, 0.248 mmol, 86%); TLC (CH_2Cl_2:EtOAc, 2:1 v/v) $R_f = 0.28$; ^{1}H NMR ($CDCl_3$, 400 MHz) δ 7.35–7.20 (m, 35H), 5.07–4.93 (m, 14H), 4.71–4.62 (m, 3H), 4.28 (dd, J = 7.1, 3.5 Hz, 1H), 4.23 (t, J = 6.4 Hz, 1H), 3.91 (dd. J = 6.9, 3.5 Hz, 1H), 1.46 (s, 3H), 1.25 (s, 3H); ^{13}C NMR ($CDCl_3$, 100 MHz) δ 137.70, 136.05–135.80 (overlapping *C*H signals with $^3J_{CP}$ couplings), 128.46–127.74, 110.66, 79.03 (C-5), 77.82, 77.77, 75.84, 74.66, 73.21, 73.19 (CH_2), 69.60–69.40 (overlapping CH_2 signals with $^3J_{CP}$ couplings), 26.46, 24.79; ^{31}P NMR ($CDCl_3$, 162 MHz) δ –1.97 (1P), –2.01 (1P), –2.38 (1P); HRMS (*m*/*z*) $[M+H]^+$ cacld for $C_{58}H_{61}O_{15}P_3$, 1091.3296; found 1091.3250.

myo-Inositol 4,5,6-trisphosphate (1)

To a solution of **4** (75 mg, 69 µmole) in methanol (4 mL) and deionised water (1mL) was added $Pd(OH)_2$ on carbon (20%, 50% water, 15mg). The suspension was stirred vigorously under an atmosphere of H_2 (balloon) for 24 h. The catalyst was removed by filtration through a PTFE syringe filter and the solvents were removed by evaporation under reduced pressure. ^{1}H NMR and ^{31}P NMR spectra of the residue showed that all benzyl groups and the isopropylidene acetal had been removed. The residue was dissolved in deionised water (5 mL) and triethylamine (0.2 mL) was added. The resulting solution was concentrated under reduced pressure, re-dissolved in deionised water, and lyophilised to give the triethylammonium salt of Ins(4,5,6)P_3 as a colourless glassy solid (46 mg, 62 µmole, 90%): ^{1}H NMR (D_2O, 400 MHz) δ 4.28 (ddd, apparent q, J = 9.4 Hz, 2H, H-4 and H-6), 4.10 (dt, apparent q, J = 9.2 Hz, 1H, H-5), 4.05 (br s, 1H, H-2), 3.67 (dd, J = 9.8, 2.5 Hz, 2H, H-1 and H-3), 3.16 (q, J = 7.3 Hz, ~19H, CH_2 of TEA^{+}), 1.24 (t, J = 7.3 Hz, ~29H, CH_3 of TEA^{+}); ^{13}C NMR (D_2O, 100 MHz) δ 77.56 (C-5), 76.97 (C-4 and C-6), 71.33 (C-2), 70.55 (C-1 and C-3), 46.57 (CH_2 of TEA^{+}), 8.20 (CH_3 of TEA^{+}); ^{31}P NMR (D_2O, 162 MHz) δ 1.15 (2P), 0.62 (1P); HRMS (*m*/*z*) [M–H]$^{-}$ cacld for $C_6H_{15}O_{15}P_3$, 418.9551; found 418.9287.

Synthesis of D-2-*O*-benzyl-*myo*-inositol 1,4,5,6-tetrakisphosphate (5) and D-2,3,-di-*O*-benzyl-*myo*-inositol 1,4,5,6-tetrakisphosphate (6).

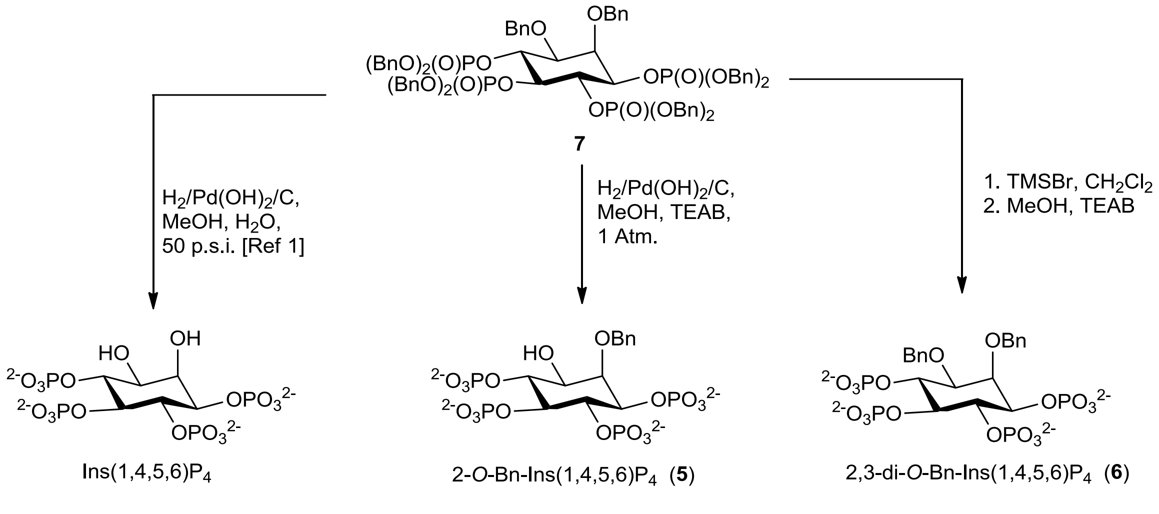

These compounds were synthesised by selective deprotections of D-2,3-di-*O*-benzyl-*myo*-inositol 1,4,5,6-tetrakis(dibenzylphosphate) (**7**). As previously reported[9], hydrogenolysis of **7** over $Pd(OH)_2$ on carbon in methanol/water at 50 p.s.i. removed all ten benzyl protecting groups, giving Ins(1,4,5,6)P_4 Noting that triethylammonium bicarbonate (TEAB) suppresses hydrogenolytic cleavage of the 2-*O*-benzyl ether in protected inositol phosphate derivatives[10], we repeated the hydrogenolysis of **7** in methanol/TEAB at atmospheric pressure. This procedure removed the eight benzyl protecting groups on phosphates, and also the 3-*O*-benzyl ether, while leaving the 2-*O*-benzyl ether completely unaffected, giving D-2-*O*-Bn-Ins(1,4,5,6)P_4 (**5**) as the triethylammonium salt. Alternatively, treatment of **7** with TMSBr in dichloromethane, followed by MeOH removed the eight benzyl ester protecting groups on phosphates, leaving the benzyl ethers at O-2 and O-3 intact, giving D-2,3-di-*O*-Bn-Ins(1,4,5,6)P_4 (**6**), again isolated as the triethylammonium salt.

D-2-*O*-benzyl-*myo*-inositol 1,4,5,6-tetrakisphosphate (5)

To a solution of D-2,3-di-*O*-benzyl-*myo*-inositol 1,4,5,6-tetrakis(dibenzylphosphate) (**7**)[9] (60 mg, 43 μmole) in methanol (8 mL) was added aqueous TEAB (1.0 moldm^{-3}, pH 7.6, 2 mL) followed by $Pd(OH)_2$ on carbon (20%, 50% water, 20 mg). The suspension was stirred vigorously under an atmosphere of H_2 (balloon) for 30 min. The catalyst was removed by filtration through a PTFE syringe filter and the solvents were removed by evaporation under reduced pressure. ^{1}H and ^{31}P NMR spectra of the residue showed that, while all benzyl protecting groups on phosphates had been removed and the 3-*O*-benzyl group had been partially cleaved, the 2-*O*-benzyl group remained unaffected. The residue was re-dissolved in methanol and aqueous TEAB as before, and a further 20 mg of catalyst was added. The suspension was stirred vigorously under an atmosphere of H_2 (balloon) for a further 7 hours, then filtered and concentrated as before. ^{1}H and ^{31}P NMR spectra of the residue now showed that that the 3-*O*-benzyl group had been completely removed, while the 2-*O*-benzyl group remained intact. The product was re-dissolved in deionised water and lyophilised to give the triethylammonium of D-2-*O*-Bn-Ins(1,4,5,6)P_4 (**5**) as a colourless solid (38 mg,

38 μmole, 88%); $[\alpha]_D$ = –11 (c = 0.7 % w/v in MeOH); ^{1}H NMR (CD_3OD, 400 MHz) δ 7.48–7.45 (m, 2H), 7.32–7.21 (m, 3H), 4.98, 4.88 (AB system, J_{AB} = 11.2 Hz, 2H), 4.66 (ddd, apparent q, J = 9.5 Hz, 1H), 4.47 (ddd, apparent q, J = 9.5 Hz, 1H), 4.23–4.15 (m, 3H), 3.68 (d, J = 9.9, 2.0 Hz, 1H), 3.15 (q, J = 7.3 Hz, ~23H, CH_2 of TEA^+), 1.27 (t, J = 7.3 Hz, ~36H, CH_3 of TEA^+); ^{13}C NMR (CD_3OD, 100 MHz) δ 141.11, 129.23, 128.97, 128.34, 81.64 (C-2), 79.74, 78.96, 78.47, 76.87 (CH_2Ph), 76.73, 73.37 (C-3), 47.33 (CH_2 of TEA^+), 9.21 (CH_3 of TEA^+); ^{31}P NMR (CD_3OD, 162 MHz) δ 1.89 (1P), 0.75 (1P), 0.68 (1P), 0.56 (1P); HRMS (*m*/*z*) $[M–H]^–$ cacld for $C_{13}H_{22}O_{18}P_4$, 588.9684; found 588.9665.

D-2,3-di-*O*-benzyl-*myo*-inositol 1,4,5,6-tetrakisphosphate (6)

To a stirred solution of D-2,3-di-*O*-benzyl-*myo*-inositol 1,4,5,6-tetrakis(dibenzylphosphate) (**7**)[9] (45 mg, 32 μmole) in dry CH_2Cl_2 (1 mL) at 0 °C under N_2 was added TMSBr (66 μL, 500 μmole). The cooling bath was removed and the solution was stirred for 2 hours and then concentrated *in vacuo*. The residue was taken up in methanol (3 mL), stirred at room temperature for 30 min and then concentrated to give a white gum. This residue was washed with diethyl ether (3 × 3 mL) and then taken up in aqueous TEAB (1.0 $moldm^{-3}$, pH 7.6, 5 mL). The resulting cloudy suspension was washed with diethyl ether (3 × 5 mL), to give a clear, colourless solution, which was concentrated under reduced pressure. The residue was re-dissolved in deionised water and lyophilised to give the triethylammonium salt of D-2,3-di-*O*-Bn-Ins(1,4,5,6)P_4 (**6**) as a colourless solid (32 mg, 29 μmole, 91%); $[\alpha]_D$ = approx. 0 (c = 0.6 % w/v in MeOH); ^{1}H NMR (D_2O, 400 MHz) δ 7.55–7.52 (m, 4H), 7.47–7.40 (m, 6H), 5.01, 4.84 (AB system, J_{AB} = 11.5 Hz, 2H), 4.72, 4.62 (AB system, J_{AB} = 11.8 Hz, 2H), 4.59 (ddd, apparent q, J = 9.2 Hz, 1H), 4.50 (ddd, apparent q, J = 9.6 Hz, 1H), 4.43 (dd, apparent t, J = 2.5 Hz, 1H), 4.24–4.16 (m, 2H), 3.67 (dd, J = 10.0, 2.5 Hz, 1H), 3.22 (q, J = 7.3 Hz, ~25H, CH_2 of TEA^+), 1.31 (t, J = 7.3 Hz, ~37H, CH_3 of TEA^+); ^{13}C NMR (D_2O, 101 MHz) δ 138.04, 137.87, 128.96, 128.64, 128.60, 128.43, 128.15, 128.02, 77.98, 77.41 (C-3), 76.59, 75.84 (C-2), 75.13, 75.00 (CH_2Ph), 72.42 (CH_2Ph), 46.67 (CH_2 of TEA^+), 8.27 (CH_3 of TEA^+); ^{31}P NMR (CD_3OD, 162

MHz) δ 1.27 (1P), 0.90 (1P), 0.67 (1P), –0.44 (1P); HRMS (*m*/*z*) $[M+Na]^+$ cacld for $C_{20}H_{28}O_{18}P_4$, 703.0118; found 703.0096.

Myo **inositol 1,3,4,6-tetrakisphosphate (7)**

Ins(1,3,4,6)P_4 was synthesised by hydrogenolytic deprotection of 2,5-di-*O*-benzyl-*myo*-inositol 1,3,4,6-tetrakisphosphate[1] in the presence of acetic acid, which accelerates the *O*-debenzylation reactions.

2,5-Di-*O*-benzyl-*myo*-inositol 1,3,4,6-tetrakisphosphate (80 mg, 0.08 mmol, triethylammonium salt) was dissolved in methanol (10 mL). Acetic acid (1 mL) and $Pd(OH)_2$ on carbon (20%, 50% water, 25mg) were added. The resulting suspension was stirred under H_2 (balloon) for 20 h at room temperature. The catalyst was removed by filtration through a PTFE syringe filter and the filtrate was evaporated under reduced pressure. The residue was then purified by ion exchange chromatography on Q Sepharose Fast Flow resin, eluting with a gradient of aqueous TEAB (0 to 2.0 $moldm^{-3}$) to afford Ins(1,3,4,6)P_4 (46 mg, 72%) as the triethylammonium salt; ^1H NMR (400 MHz, D_2O) δ 4.30–4.23 (m, 3H, H-2, H-4 and H-6); 4.02 (dt, *J* = 2.3, 9.4 Hz, 2H, C-1-H and C-3-H), 3.56 (t, *J* = 9.4 Hz, 1H, C-5-H), 3.08 (q, *J* = 7.4 Hz, ~23H, CH_2 of TEA^+), 1.16 (t, *J* = 7.4 Hz, ~35H, CH_3 of TEA^+);^{13}C NMR (D_2O, 100 MHz) δ 76.7 (C-4 and C-6), 74.1 (t, C-1 and C-3), 72.9 (C-5), 70.6 (C-2), 46.5 (CH_2 of TEA^+), 8.1 (CH_3 of TEA^+);^{31}P NMR (162 MHz, ^1H-decoupled, D_2O, TEA added) δ 4.29 (2P) and 3.89 (2P); HRMS (*m/z*): $[M–H]^–$ Calcd for $C_6H_{15}O_{18}P_4$ 498.9214; found 498.9202.

Synthesis of D-*myo*-Inositol 1,4,5,6-tetrakisphosphate (8)

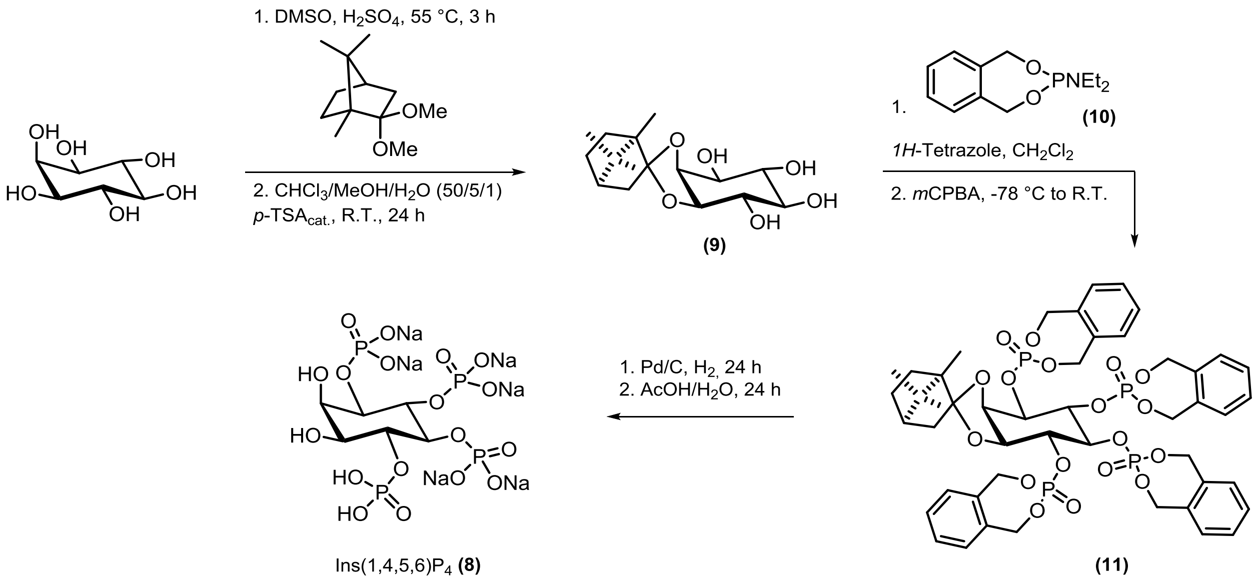

1D-2,3-O-(D-1',7',7'-Trimethy1[2.2.1]bicyclohept-2'-ylidene)-*myo*-inositol (9)

myo-Inositol (12.9 g, 71.8 mmol, 1 equiv.) was solubilised in DMSO (100 mL) by heating the suspension at 110 °C. The resulting solution was treated with D-camphor dimethyl acetal (22.5 g, 113.5 mmol, 1.6 equiv.) and TMS-triflate (0.3 mL). The mixture was heated at 90 °C over a period of 3 h. After this time, ethylene glycol (2.3 g) and chloroform (240 mL) were added, and the mixture was heated at 50 °C for 2 h. The mixture was concentrated under vacuum, redissolved in chloroform-ether (1:2, 500 mL), and stirred at 4 °C for 24 h. After this time colourless precipitate formed and was separated. The clear supernatant was concentrated under vacuum and added to a solution of chloroform, methanol (9:1, 200 mL) and 4-toluenesulfonic acid (0.7 g), and the solution was stirred at room temperature for 12 h. The solid product was removed by filtration, and both batches of the solid product were crystallised from the minimum amount of methanol. A second crystallisation from methanol gave 1D-2,3-O-(D-1',7',7'-trimethy1[2.2.1]bicyclohept-2'-ylidene)-*myo*-inositol compound (4.3 g, 19%) essentially free from other isomers. A further three crystallisations of the mother liquor, from methanol, afforded another crop of (**9**) (2.2 g, 10%) as a colourless crystalline solid; R_f 0.25 ($CHCl_3$/MeOH: 4:1); m.p. 231-232 °C (from methanol) [lit.[11] 230-231 °C]; $[\alpha]_D^{20}$

= +43.9 (*c* 1.9, pyridine) [lit.[11] $[\alpha]_D^{20}$ = +44.3 (*c* 1.9, pyridine)]; ^{1}H NMR (400 MHz, MeOD) δ 4.27 (dd, *J* = 5.5, 5.5 Hz, 1H), 3.78 (dd, *J* = 7.1, 5.5 Hz, 1H), 3.69 (dd, *J* = 9.7, 4.2 Hz, 1H), 3.52 (dd, *J* = 9.7, 9.7 Hz, 1H), 3.44 (dd, *J* = 10.1, 7.1 Hz, 1H), 3.13 (t, *J* = 9.7 Hz, 1H), 2.13 - 1.95 (m, 2H), 1.80 - 1.64 (m, 2H), 1.50 (d, *J* = 13.0 Hz, 1H), 1.45-1.32 (m, 1H), 1.27-1.14 (m, 1H), 1.04 (s, 3H), 0.89 (s, 3H), 0.86 (s, 3H); ^{13}C NMR (101 MHz, MeOD) δ 118.9, 77.9, 77.7, 77.4, 75.1, 73.8, 71.8, 53.6, 52.6, 46.6, 46.5, 30.6, 28.0, 21.2, 20.9, 10.4; HRMS (*m*/*z*) $[M+H]^+$ cacld for $C_{16}H_{26}O_6$, 315.3846; found 315.3832; Anal. Calcd for $C_{15}H_{26}O_6$: C, 61.1; H, 8.33. Found: C, 60.94; H, 8.56. To prevent acetal group migration or deprotection triethylamine was added to methanol used for crystallisation, and the final compound was stored at −10 °C. A mixture of other three diastereomers was obtained following crystallisation of the mother liquor. The data are in good agreement with the literature values[11].

_N_,_N_-Diethyl-1,3,2-benzodioxaphosphol-2-amine (10)

1,2-Benzenedimethanol (0.8 g, 5.74 mmol, 1 equiv.) and two molar equivalents of anhydrous triethylamine in anhydrous ether/THF was added dropwise over a 1 h period to a solution of the diethylaminophosphorus dichloride (1.0 g, 5.74 mmol, 1 equiv.) in diethyl ether (0.01-0.05 M) at −78 °C under a dry, argon atmosphere. A colourless precipitate of triethylamine hydrochloride was formed. The reaction was allowed to warm to room temperature over a period of 1–2 h. After this time, the reaction mixture was filtered through a coarse sintered glass funnel under an argon atmosphere, and the resulting ether solution was concentrated under high vacuum. The product **10** (1.18 g, 86%) was obtained as a colourless oil; ^{1}H NMR (250 MHz, $CDCl_3$) δ 7.37-7.09 (m, 4H), 5.21 (dd, *J* = 13.8, 7.0 Hz, 2H), 4.94 (dd, *J* = 19.5, 13.8 Hz, 2H), 3.21 (m, 4H), 1.13 (t, *J* = 7.1 Hz, 6H); ^{13}C NMR (63 MHz, $CDCl_3$) δ 138.2 (2C), 128.2 (2C), 127.9 (2C), 65.8 (d, *J* = 10.2 Hz, 2C), 38.5 (d, *J* = 20.8 Hz, 2C), 15.0 (d, *J* = 2.9 Hz, 2C); ^{31}P NMR (101 MHz, $CDCl_3$) δ 146.59 (1P).

D-1,4,5,6-Tetra-_O_-(2-oxo-5.6-benzo-1,3,2-dioxaphosphep-2-yl)-1,2-_O_-(D-1,7,7-trimethyl-[2.2.l]bicyclohept-2-ylidene)-_myo_-inositol (11)

N,*N*-Diethyl-1,3,2-benzodioxaphosphol-2-amine (**10**) (0.39 g, 1.87 mmol, 5.7 equiv.) dissolved in dry CH_2Cl_2 (10 mL), was stirred with *1H*-tetrazole (0.45 M solution in acetonitrile, 8.9 mL, 4.0 mmol, 12 equiv.) under an atmosphere of nitrogen for 10 min. Tetrol (**9**) (0.11 g, 0.33 mmol, 1 equiv.) was added and the resulting mixture was stirred for 1 h (until the solid was fully dissolved). The mixture was cooled to –78 °C and 3-chloroperoxybenzoic acid (0.32 g, 1.87 mmol) was added. The mixture was stirred at –78 °C for 30 min, and then allowed to warm to room temperature and stirred for a further 1 h. The reaction mixture was diluted with EtOAc (30 mL) and washed consecutively with a saturated aqueous solution of sodium bisulfite and an aqueous saturated solution of sodium bicarbonate. After concentration under reduced pressure, the residue was purified by column chromatography on silica gel (chloroform/acetone: 30:1) to yield (**11**) (0.31 g, 89%) as a glass; R_f 0.16 (chloroform/acetone: 30:1); ^{1}H NMR (500 MHz, $CDCl_3$) δ 7.41-7.26 (m, 16H), 5.67-4.79 (m, 20H), 4.77 (dd, *J* = 4.7, 4.7 Hz, 1H), 4.25 (dd, *J* = 6.9, 5.3 Hz, 1H), 2.17-2.00 (m, 1H), 1.83-1.80 (m, 1H), 1.78-1.74 (m, 1H), 1.63 (d, *J* = 13.2 Hz, 1H), 1.48 (td, *J* = 12.3, 4.6 Hz, 1H), 1.31-1.22 (m, 1H), 1.04 (s, 3H), 1.02 (s, 3H), 0.88 (s, 3H). ^{13}C NMR (126 MHz, $CDCl_3$) δ 135.9, 135.73, 135.67, 135.55, 135.47, 135.33, 135.28, 135.23, 130.15, 129.54, 129.49 (2C), 129.46, 129.35 (2C), 129.29 (2C), 129.27, 129.25, 129.21, 129.17, 129.11, 129.06, 128.9, 120.2, 79.4, 77.4, 76.6, 74.6, 74.4, 73.8, 69.8-69.6 (m, 1C), 69.6-69.0 (m, 4C), 68.7 (d, *J* = 6.8 Hz), 68.5 (d, *J* = 6.6 Hz), 66.9 (d, *J* = 5.1 Hz), 52.1, 48.1, 45.3, 45.2, 29.3, 27.0, 20.42, 20.39, 9.6; ^{31}P NMR (202 MHz, $CDCl_3$) δ –0.96 (1P), –2.91 (1P), –2.95 (1P), –3.08 (1P); HRMS (*m/z*) $[M+H]^+$ cacld for $C_{48}H_{55}O_{18}P_4$, 1043.8457; found 1043.8424; LRMS *m/z* (ES^+) 1043 ($[M+H]^+$, 100%), 786 (25), 503 (75), 284 (20). The data are in good agreement with the literature values for the enantiomer of compound **11**[12].

D-*myo*-Inositol 1,4,5,6-tetrakisphosphate (8)

To a solution of (**11**) (30 mg, 28.77 □mol) in methanol (2 mL) was added palladium on carbon (10%, 28 mg) and the mixture was stirred under hydrogen at room temperature for 5h. After catalyst removal and evaporation of solvent, 1 mL of water and 1 mL of acetic acid were added and the resulting mixture was stirred at room temperature for 18 h. After this time, the

reaction mixture was evaporated to dryness and the residue was dissolved in a minimum amount of water. Sodium hydroxide (1 mL of solution at 10% in water) was added and the resulting solution was diluted with methanol to precipitate (**8**) as its hexasodium salt (18 mg, 83%); mp 200-201°C (from methanol) [lit. [12] 200 °C (dec) for the enantiomer]; ^{1}H NMR (500 MHz, D_2O) δ 4.27 (q, *J* = 9.4 Hz, 1H), 4.13 (dd, *J* = 16.0, 5.9 Hz, 2H), 4.02-3.82 (m, 2H), 3.58 (dd, *J* = 9.7, 2.7 Hz, 1H); ^{13}C NMR (126 MHz, D_2O) δ 77.9, 76.4, 76.2, 74.6, 71.4 (2C); ^{31}P NMR (202 MHz, D_2O) δ 4.68 (1P), 3.23 (1P), 2.90 (1P), 2.38 (1P); HRMS (*m*/*z*) $[M+H]^+$ cacld for $C_6H_{11}Na_6O_{18}P_4$, 632.8267; found 632.8256; LRMS *m/z* (ES^-) 418 ($[C_6H_{14}O_{15}P_3]^-$, 100%), 609 $[C_6H_{10}Na_5O_{18}P_4]^-$ (15), 586 $[C_6H_{11}Na_4O_{18}P_4]^-$ (20), 563 $[C_6H_{12}Na_3O_{18}P_4]^-$ (60), 540 $[C_6H_{13}Na_2O_{18}P_4]^-$ (45), 441 $[C_6H_{13}NaO_{15}P_3]^-$ (75),

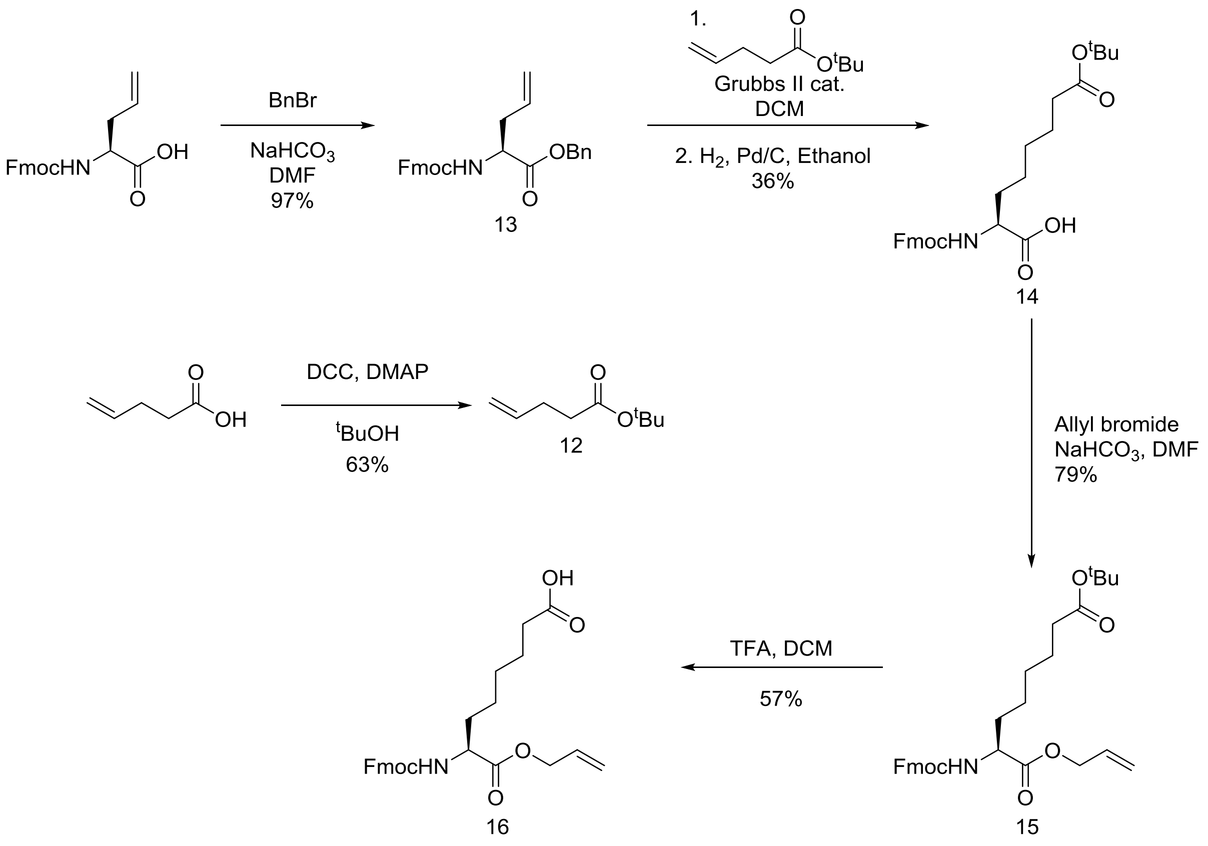

Fmoc-L-allylglycine-OBn (13)

Fmoc-allylglycine-OH (100 mg, 0.30 mmol, 1 equiv.) and sodium hydrogen carbonate (124 mg, 1.48 mmol, 5 equiv.) were taken up in *N,N*-dimethylformamide (0.6 mL, 0.5 M). Benzyl bromide (71 µL, 0.59 mmol, 2 equiv.) was added and the reaction allowed to stir at room temperature for 20 h. Water (10 mL) was added to crash out the product. The resulting solution was extracted with dichloromethane (3 x 10 mL). The combined organic layers were washed with 5% aqueous lithium chloride solution (3 x 30 mL) and brine (3 x 30 mL), dried ($MgSO_4$) and concentrated to give a colourless oil. The oil was dissolved in chloroform and left to slowly crystallise, yielding Fmoc-allylglycine-OBn as white crystals (125 mg, 97%); mp 76-78 °C (from chloroform); $[\alpha]_D^{20}$ -10.2 (c = 0.5 in $CHCl_3$); ^{1}H NMR (300 MHz, $CDCl_3$) δ 7.78 (d, *J* = 7.6 Hz, 2H), 7.61 (d, *J* = 7.3 Hz, 2H), 7.45-7.29 (m, 9H), 5.75-5.61 (m, 1H), 5.37 (d, *J* = 7.8 Hz, 1H), 5.27-5.06 (m, 4H), 4.59-4.50 (m, 1H), 4.41 (d, *J* = 7.4 Hz, 2H), 4.24 (t, *J* = 6.8 Hz, 1H), 2.70-2.49 (m, 2H); ^{13}C NMR (100MHz, $CDCl_3$) δ 171.6, 155.7, 143.8, 141.3, 135.2, 131.9, 128.6, 128.5, 128.4, 127.7, 127.0, 125.1, 120.0, 119.5, 67.3, 67.1, 53.3, 47.1, 36.7; IR (FTIR): 3328,

3036, 2952, 1728, 1691, 1536, 1450, 1259, 1182; HRMS (*m/z*) $[M+Na]^+$ calcd for $C_{27}H_{25}NO_4Na$, 450.1681; found 450.1677.

_t_-Butyl pent-4-enoate (12)

To a solution of 4-pentenoic acid (22 g, 220 mmol, 1 equiv.) 4-(dimethylamino)pyridine (436 mg, 3.6 mmol, 0.02 equiv.) and *t*-butanol (85 mL, 892 mmol, 4 equiv.), was added dicyclohexylcarbodiimide (62 g, 300 mmol, 1.5 equiv.) at 0 °C. The reaction was stirred at 0 °C for 25 mins, then left to stir at room temperature for 18 h. The dicyclohexylurea precipitate was removed by filtration through celite and the filtrate concentrated *in vacuo*. Purification was carried out by flash column chromatography (0-20% ethyl acetate/hexane) to give *t*-butyl pent-4-enoate (23.4 g, 63%) as a colourless oil; ^{1}H NMR (300 MHz, $CDCl_3$) δ: 5.69-5.88 (m, 1H), 4.91-5.07 (m, 2H), 2.22-2.39 (m, 4H), 1.42 (s, 9H); ^{13}C NMR (75 MHz, $CDCl_3$) δ: 172.6, 137.3, 115.5, 80.4, 34.8, 29.2, 28.2; IR (FTIR): 2979, 2931, 1730, 1367, 1256, 1148. HRMS (*m/z*) $[M+H]^+$ calcd for $C_9H_{17}O_2$, 157.1229; found 157.1230.

Fmoc-octanoate(_t_Bu)-OH (14)

To a solution of Fmoc-allylGlycine-OBn (13) (1.0 g, 4.66 mmol, 1 equiv.) and ^{t}Bu pent-4-enoate (12) (1.99 g, 23.3 mmol, 5 equiv.) in dichloromethane (24 mL, 0.1 M) was added Grubbs 2nd generation catalyst (100 mg, 0.23 mmol, 5 mol%). The solution was heated to reflux for 2 h, cooled to room temperature and the solvent evaporated *in vacuo*. The residue was taken up in ethanol (24 mL, 0.1 M) followed by the addition of Pd/C (20 mg, 10% loading). The flask was flushed with hydrogen then left under a positive hydrogen pressure for 72 hours at room temperature. The solution was filtered through celite and the solution concentrated *in vacuo*. Purification was carried out using flash column chromatography (0-5% methanol/dichloromethane) to give Fmoc-octanoate(*t*Bu)-OH as a light brown powder (774 mg, 36%); $[\alpha]_D^{20}$ -9.6 (c = 0.5 in $CHCl_3$); ^{1}H NMR (300 MHz, $CDCl_3$) δ: 7.77 (d, *J* = 7.6 Hz, 2H), 7.59 (d, *J* = 7.3 Hz, 2H), 7.39 (t, *J* = 7.3 Hz, 2H), 7.33 (t, *J* = 7.0 Hz, 2H), 4.42 (d, *J* = 6.7 Hz, 2H), 4.23 (t, *J* = 6.7 Hz, 1H), 2.22 (td, *J* = 7.0, 2.3 Hz, 2H), 1.82-2.00 (m, 1H), 1.67-1.81 (m, 1H), 1.50-1.67 (m, 3H), 1.44-1.47 (m, 9H), 1.28-1.42 (m, 4H); ^{13}C NMR (500 MHz, $CDCl_3$) δ: 176.5, 173.3, 156.1, 143.7, 141.3,

127.7, 127.0, 125.1, 120.0, 80.2, 67.1, 53.7, 47.2, 35.4, 33.6, 28.6, 28.1, 24.9, 24.7; IR (FTIR) cm^{-1}: 3331, 2932, 2858, 1715, 1526, 1449, 1211, 1151; HRMS (*m/z*) $[M+H]^+$ calcd for $C_{27}H_{34}NO_6$, 468.2386; found 468.2383.

Fmoc- octanoate(*t*Bu)-OAllyl (15)

To a suspension of Fmoc-octanoate(*t*Bu)-OH (14) (100 mg, 0.21 mmol, 1 equiv.) and sodium hydrogen carbonate (81 mg, 0.96 mmol, 4.5 equiv.) in *N,N*-dimethylformamide (3.6 mL, 0.06 M) was added allyl bromide (23 µL, 0.27 mmol, 1.25 equiv.). The reaction was allowed to stir at room temperature for 18h, and then concentrated *in vacuo.* The residue was taken up in ethyl acetate (5 mL) and washed with 5% aqueous lithium chloride solution (3 x 5 mL) followed by brine (3 x 5 mL). The organics were combined, dried ($MgSO_4$) and concentrated *in vacuo*. Purification was carried out by flash column chromatography (20% ethyl acetate/hexane) to give Fmoc-octanoate(*t*Bu)-OAllyl (85 mg, 79%) as a colourless gum; $[\alpha]_D^{20}$ -10.4 (c = 0.5 in $CHCl_3$); ^1H NMR (300 MHz, $CDCl_3$) δ: 7.77 (d, *J* = 7.3 Hz, 2H), 7.60 (d, *J* = 7.6 Hz, 2H), 7.37-7.44 (m, 2H), 7.33 (td, *J* = 7.6, 1.2 Hz, 2H), 5.92 (ddt, *J* = 5.6, 11.4, 16.4 Hz, 1H), 5.24-5.40 (m, 2H), 4.66, (d, *J* = 5.6 Hz, 2H), 4.36-4.47 (m, 3H), 4.24 (t, *J* = 6.7 Hz, 1H), 2.21 (t, *J* = 7.5 Hz, 2H), 1.80-1.95 (m, 1H), 1.53-1.77 (m, 3H), 1.46 (s, 9H), 1.30-1.41 (m, 4H); ^{13}C NMR (75 MHz, $CDCl_3$) δ: 173.0, 172.3, 155.9, 143.8, 141.3, 131.2, 127.7, 127.0, 125.1, 120.0, 118.9, 80.0, 67.0, 65.9, 53.9, 47.2, 35.4, 32.5, 28.6, 28.1, 24.9, 24.8; IR (FTIR) cm^{-1}: 3336, 2935, 1722, 1525, 1450, 1366, 1248, 1151; HRMS (*m/z*) $[M+Na]^+$ calcd for $C_{30}H_{37}NO_6Na$, 530.2519; found 530.2551.

Fmoc-oxooctanoic acid-OAllyl (16)

Fmoc-octanoate(*t*Bu)-OAllyl (15) (75 mg, 0.15 mmol, 1 equiv.) was stirred in 90% trifluoroacetic acid/dichloromethane (1 mL) for 30 minutes then concentrated. Purification was carried out by flash column chromatography (0-3% methanol/dichloromethane) to give the product (38 mg, 57%); $[\alpha]_D^{20}$ -13.9 (c = 0.5 in $CHCl_3$); ^1H NMR (300 MHz, $CDCl_3$) δ: 7.77 (d, *J* = 7.3 Hz, 2H), 7.61

(d, *J* = 7.0 Hz, 2H), 7.41 (t, *J* = 7.0 Hz, 2H), 7.32 (t, *J* = 7.3 Hz, 2H), 5.93 (ddt, *J* = 16.4, 11.1, 5.8 Hz, 1H), 4.66 (d, *J* = 5.3 Hz, 2H), 4.42 - 4.39 (m, 3H), 4.24 (t, *J* = 6.7 Hz, 1H), 2.35 (t, *J* = 7.5 Hz, 2H), 1.80 - 2.00 (m, 1H), 1.54-1.79 (m, 4H), 1.29-1.48 (m, 4H); ^{13}C NMR (75 MHz, $CDCl_3$) δ: 179.2, 172.4, 156.0, 143.8, 141.4, 131.5, 127.7, 127.1, 125.1, 120.0, 119.0, 67.1, 66.1, 53.9, 47.2, 33.8, 32.5, 28.6, 24.9, 24.4; IR (FTIR) cm^{-1}: 3320, 2934, 1700, 1525, 1449, 1191; HRMS (*m/z*) $[M+H]^+$ calcd for $C_{26}H_{30}NO_6$, 452.2073; found 452.2082.

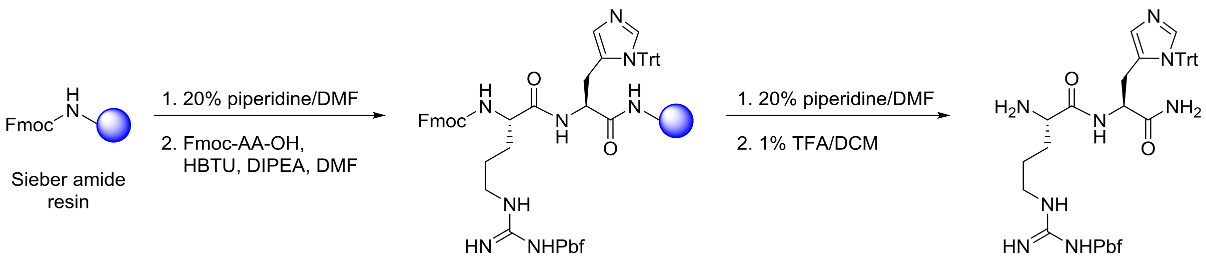

17

Fmoc deprotection on resin: Fmoc-protected peptide on resin was treated with 20 % piperidine in *N,N*-dimethylformamide (v/v, 1 mL/50 mg resin) at room temperature (2 x 15 mins), filtered then washed with *N,N*-dimethylformamide (3 x 3 mL), methanol (3 x 3 mL) and dichloromethane (3 x 3 mL). Kaiser test was used to confirm positive test for a free amine.

Manual coupling of Fmoc-protected amino acids: Fmoc-protected amino acid (3 equiv.) was dissolved in *N,N*-dimethylformamide, then *N,N*-diisopropylethylamine (6 equiv.) and *O*-(Benzotriazol-1-yl)-*N,N,N',N'*-tetramethyluronium hexafluorophosphate (HBTU) (3 equiv.) were added. The resulting solution was shaken for 5 mins before addition to the prepared resin and shaken for 1 h. The resin was washed with *N,N*-dimethylformamide (3 x 3 mL), methanol (3 x 3 mL) and dichloromethane (3 x 3 mL). Kaiser test was used to confirm negative test for a free amine.

Capping of unreacted primary amines on resin: Acetic anhydride (2 equiv.) and diisopropylethylamine (4 equiv.) were taken up in *N,N*-dimethylformamide, added to the resin and shaken for 30 mins. The resin was filtered then washed with *N,N*-dimethylformamide (3 x 3 mL), methanol (3 x 3 mL) and dichloromethane (3 x 3 mL).

Cleavage of Arg(Pbf)-His(Trt)-NH$_2$ from Sieber amide resin: Peptide on resin was treated with 1% trifluoroacetic acid in dichloromethane at room temperature for 3 h. The filtrate was collected and the solvent removed. The resulting residue was taken up in water and lyophilised.

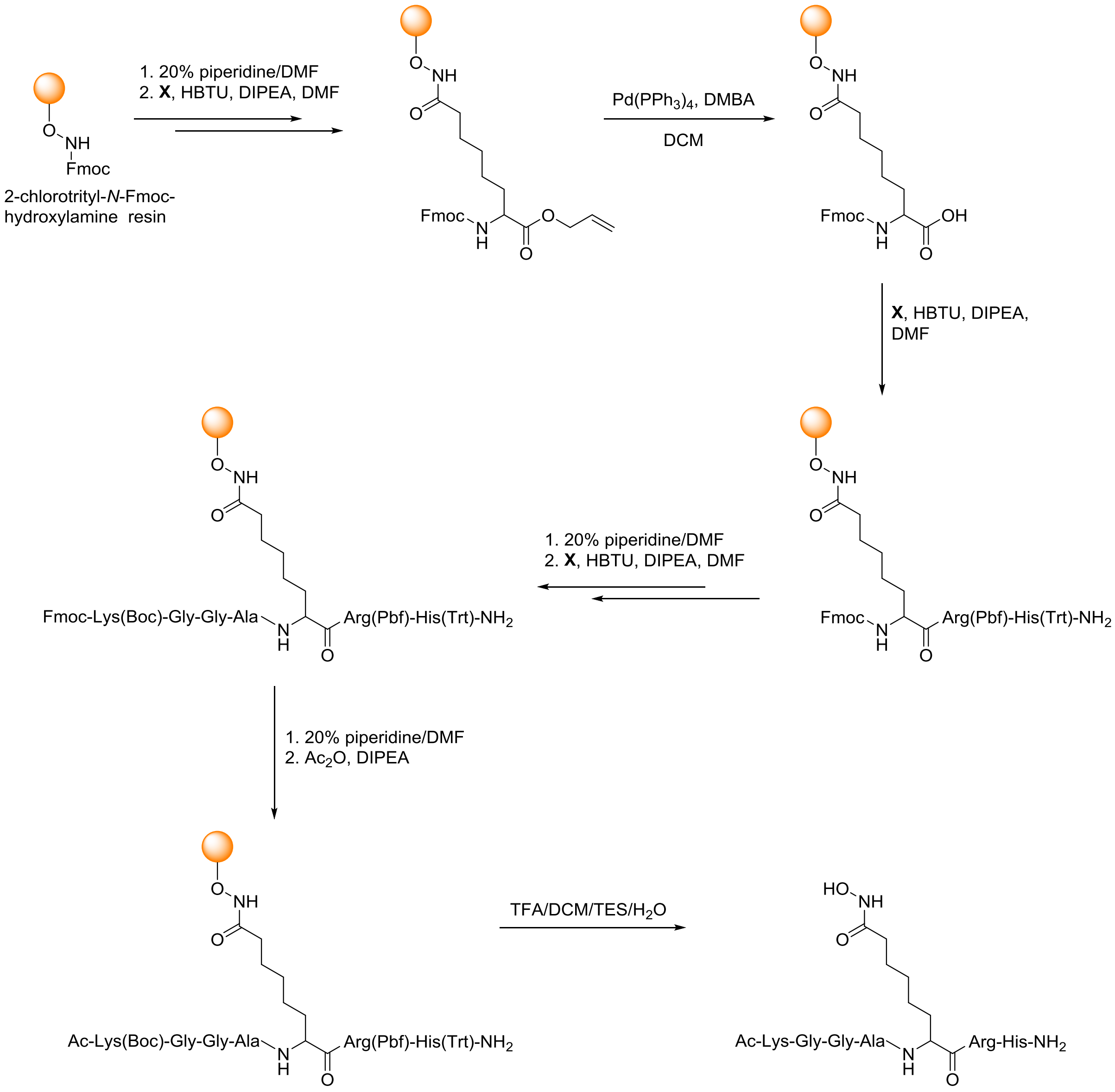

Initial loading onto 2-chlorotityl-N-Fmoc- hydroxylamine resin: 2-Chlorotityl-*N*-Fmoc- hydroxylamine resin (0.69 mmol/g resin substitution) was swollen in *N,N*-dimethylformamide for 30 mins, filtered, then treated with 20% piperidine/*N,N*-dimethylformamide (2 x 30 min). The resin was washed with *N,N*-dimethylformamide (3 x 3 mL), methanol (3 x 3 mL) and dichloromethane (3 x 3 mL). Fmoc-oxooctanoic acid-OAllyl (1.2 equiv.) was taken up in *N,N*-

dimethylformamide (0.1 M) followed by the addition of *N,N*-diisopropylethylamine (2.4 equiv.) and HBTU (1.2 equiv.). The resulting solution was allowed to activate for 2 mins before addition to the prepared resin. The resin suspension was shaken for 1 h then filtered. The resin was washed with *N,N*-dimethylformamide (3 x 3 mL), methanol (3 x 3 mL) and dichloromethane (3 x 3 mL), then dried under a stream of nitrogen.

Allyl deprotection: Functionalised resin was swollen in dichloromethane for 30 mins, filtered and taken up in dichloromethane (0.1 M) and degassed for 2 mins. 1,3-Dimethylbarbituric acid (1.3 equiv.) was added and the resin suspension agitated before $Pd(PPh_3)_4$ (0.11 equiv.) was also added. The vessel was flushed with nitrogen and rocked for 1 h. The resin was drained and washed with dichloromethane until the solvent appeared colourless, then washed with *N,N*-dimethylformamide (3 x 3mL), and dichloromethane (3 x 3mL), then dried under a stream of nitrogen.

Dipeptide coupling onto Fmoc-oxooctanoic acid-OH functionalised 2-chlorotityl-N-Fmoc-hydroxylamine resin: Functionalised resin was swollen in *N,N*-dimethylformamide for 30 mins then filtered. The resin was taken up in HBTU (1.1 equiv.) in *N,N*-dimethylformamide (0.5 M) with *N,N*-diisopropylethylamine (5 equiv.) and shaken for 5 mins. H-Arg(Pbf)-His(Trt)-NH_2 (1 equiv.) was added to the resin suspension and shaken for 1 h. The resin was filtered and then washed with *N,N*-dimethylformamide (3 x 3 mL), methanol (3 x 3 mL) and dichloromethane (3 x 3 mL).

Cleavage of peptide side chain protecting groups and of peptide from resin: Peptide on resin was treated with trifluoroacetic acid/dichloromethane/triethylsilane/water (90:5:2.5:2.5) at room temperature for 3 h. The peptide was precipitated and washed three times in cold diethyl ether and spun down to a pellet before the diethyl ether removed and the peptide dried under a steady stream of nitrogen.

General procedure for automated peptide synthesis: The peptide was synthesized on a CEM Liberty1 automated microwave-assisted solid-phase peptide synthesizer (CEM Corporation) using a 30 mL Teflon reactor vessel at 50 µmol scale using Rink Amide MBHA resin (substitution: 0.60 mmol/g). Fmoc-protected amino acids were made up as a solution of 0.2 M in *N,N*-dimethylformamide to give 5 equivalents relative to the resin. The activator

was made up to 0.25 M HCTU in *N,N*-dimethylformamide and the activator base was made up to 1 M *N,N*-diisopropylethylamine in *N*-methylpyrrolidine. Deprotection solution was made up to a 20% v/v piperidine in *N,N*-dimethylformamide. Double coupling of Fmoc-Arg(Pbf)-OH was carried out at room temperature for 25 mins, then heated to 75 °C for 5 mins, drained and a second coupling step carried out at 75 °C for 5 mins. Coupling of Fmoc-His(Trt)-OH was carried out at room temperature for 2 mins followed by 50 °C for 4 mins. All other amino acids were coupled at 75 °C for 5 mins. Deprotections were carried out at 75 °C for 30 s, followed by a second deprotection at 75 °C for 3 mins.

Manual coupling of fluorescein isothiocyanate: Fluorescein isothiocyanate (2 equiv.) was dissolved in *N*-methylpyrrolidine (0.05 M) followed by addition of *N,N*-diisopropylethylamine (4 equiv.). The resulting solution was added to the prepared resin. The resin suspension was shaken in the dark at room temperature for 18 h, then the resin drained and washed with *N,N*-dimethylformamide (3 x 3 mL), methanol (3 x 3 mL) and dichloromethane (3 x 3 mL).

Cleavage of peptide from resin: Peptide on resin was treated with trifluoroacetic acid/triethylsilane/water (95:2.5:2.5) at room temperature for 3 h before being drained and the trifluoroacetic acid blown off with a stream of nitrogen. The peptide was precipitated and washed three times in cold diethyl ether and spun down to a pellet before the diethyl ether removed and the peptide dried under a steady stream of nitrogen.

Peptide purification: Crude peptides were purified by reverse-phase HPLC using a Dionex Ultimate 3000 system with a Phenomenex Gemini-NX 5μm C18 110Å AXIA packed column with dimensions 250 x 21.20 mm and purity confirmed by analytical reverse-phase HPLC using a Dionex Ultimate 3000 system with a Phenomenex Gemini-NX 5μm C18 110Å packed column with dimensions 150 x 4.60 mm and by LC-MS as above. Water was removed by lyophilisation using an Edwards Modulyo lyophylizer.

Ac-Lys-Gly-Gly-Ala-Hao-Arg-His-NH$_2$: 0.3 mg, 1% (91% purity); HRMS (*m/z*) $[M+H]^+$ calcd for $C_{35}H_{62}N_{15}O_{10}$, 852.4804; found 852.4839; analytical HPLC 5-50% acetonitrile/H_2O (0.1% trifluoroacetic acid), 15 min gradient, T_R

8.337 min; 5-20% acetonitrile/water (0.1% trifluoroacetic acid), 15 min gradient, T_R 10.180 min.

FITC-Ahx-Lys-Gly-Gly-Ala-Hao-Arg-His-NH$_2$: 13 mg, 20% (95% purity); HRMS (*m/z*) $[M+H]^+$ calcd for $C_{60}H_{82}N_{17}O_{14}S$, 1296.5948; found 1296.5983; analytical HPLC 5-100% acetonitrile/water (0.1% trifluoroacetic acid), 30 min gradient, T_R 16.900min; 5-100% acetonitrile/water (0.1% trifluoroacetic acid), 60 min gradient, T_R 18.440 min.

FITC-Ahx-Ser-Glu-Arg-Gly-Lys(Ac)Gly-GLy-Lys-NH$_2$ (H4(1-8)K5Ac): 6 mg, 10% (91% purity); HRMS (m/z)) [M+H]+ calcd for $C_{58}H_{81}N_{16}O_{16}S$; 1289.5737; found 1289.5824; analytical HPLC 5-100% acetonitrile/H_2O (0.1% trifluoroacetic acid), 15 min gradient, T_R 13.967 min; 5-100% acetonitrile/H_2O (0.1% trifluoroacetic acid), 30 min gradient, T_R 17.283 min.

FITC-Ahx-Thr-Ala-Arg-Lys(Ac)-Ser-Thr-Gly-NH$_2$ (H3(6-12)K9Ac): 12 mg, 17% (98% purity); HRMS (m/z)) [M+H]+ calcd for $C_{57}H_{79}N_{14}O_{17}S$; 1263.5468; found 1263.5522; analytical HPLC 5-100% acetonitrile/H_2O (0.1% trifluoroacetic acid), 15 min gradient, T_R 14.490 min; 5-100% acetonitrile/H_2O (0.1% trifluoroacetic acid), 30 min gradient, T_R 18.287 min.

FITC-Ahx-Thr-Gly-Gly-Lys(Ac)-Ala-Pro-Arg-NH$_2$ (H3(11-17)K14Ac): 11 mg, 16% (98% purity); HRMS (m/z)) [M+H]+ calcd for $C_{57}H_{77}N_{14}O_{15}S$; 1229.5414; found 1229.5468; analytical HPLC 5-100% acetonitrile/H2O (0.1% trifluoroacetic acid), 15 min gradient, T_R 14.580 min; 5-100% acetonitrile/H_2O (0.1% trifluoroacetic acid), 30 min gradient, T_R 18.470 min.

FITC-Ahx-Lys-Ala-Ala-Arg-Lys(Ac)-Ser-Ala-NH$_2$ (H3(23-29)K27Ac): 18 mg, 14% (98% purity); HRMS (m/z) [M+H]+ calcd for $C_{59}H_{84}N_{15}O_{15}S$; 1274.5992; found 1274.5996; analytical HPLC 5-100% acetonitrile/H_2O (0.1% trifluoroacetic acid), 15 min gradient, T_R 14.080 min; 5-50% acetonitrile/H_2O (0.1% trifluoroacetic acid), 30 min gradient, T_R 10.237 min.

FITC-Ahx-Lys-Gly-Gly-Ala-Lys(Ac)-Arg-His-NH2 (H3(12-18)K16Ac): 42 mg, 33% (>99% purity); HRMS (*m/z*) [M+H]+ calcd for $C_{60}H_{82}N_{17}O_{14}S$,

1296.5948; found 1296.5977; analytical HPLC 5-100% acetonitrile/water (0.1% trifluoroacetic acid), 15 min gradient, T_R 13.757 min; 5-100% acetonitrile/water (0.1% trifluoroacetic acid), 30 min gradient, T_R 18.443 min.

NMR SPECTRA

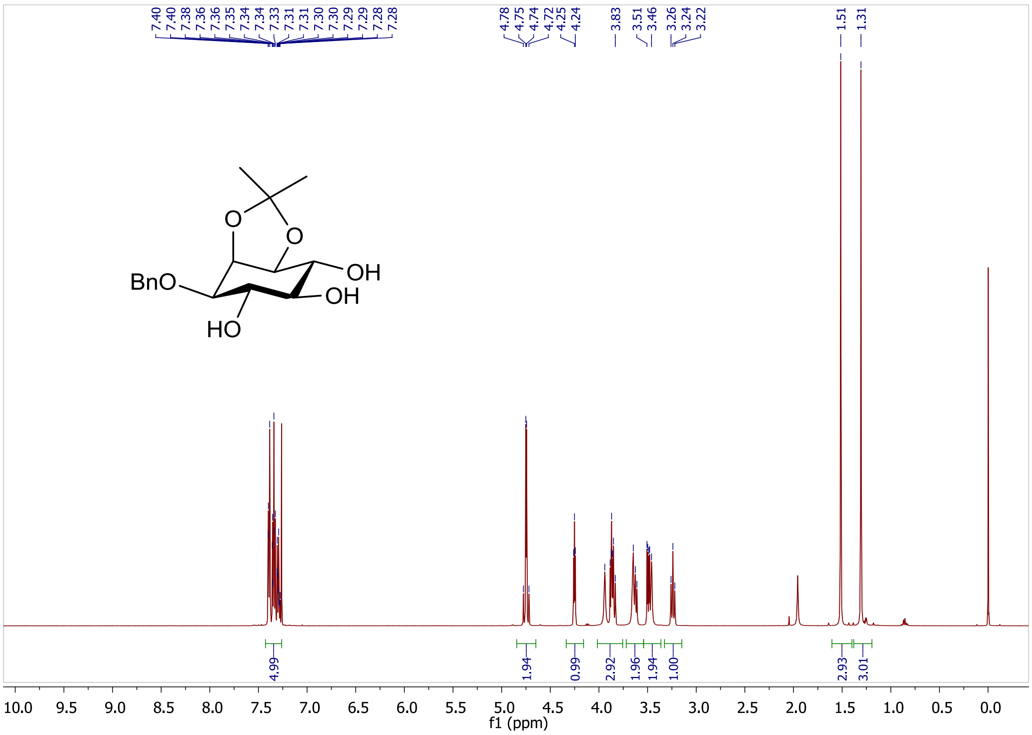

DL-3-*O*-benzyl-1,2-*O*-isopropylidene-*myo*-inositol (3): ^{1}H NMR ($CDCl_3$, 400 MHz)

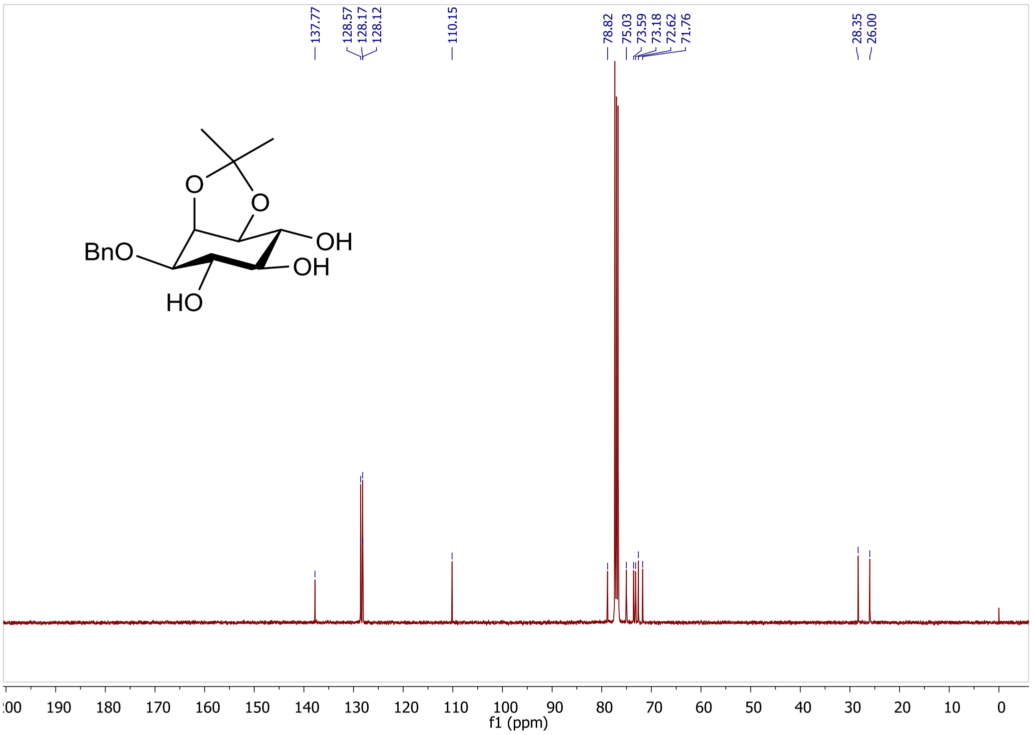

DL-3-*O*-benzyl-1,2-*O*-isopropylidene-*myo*-inositol (3): ^{13}C NMR ($CDCl_3$, 100 MHz)

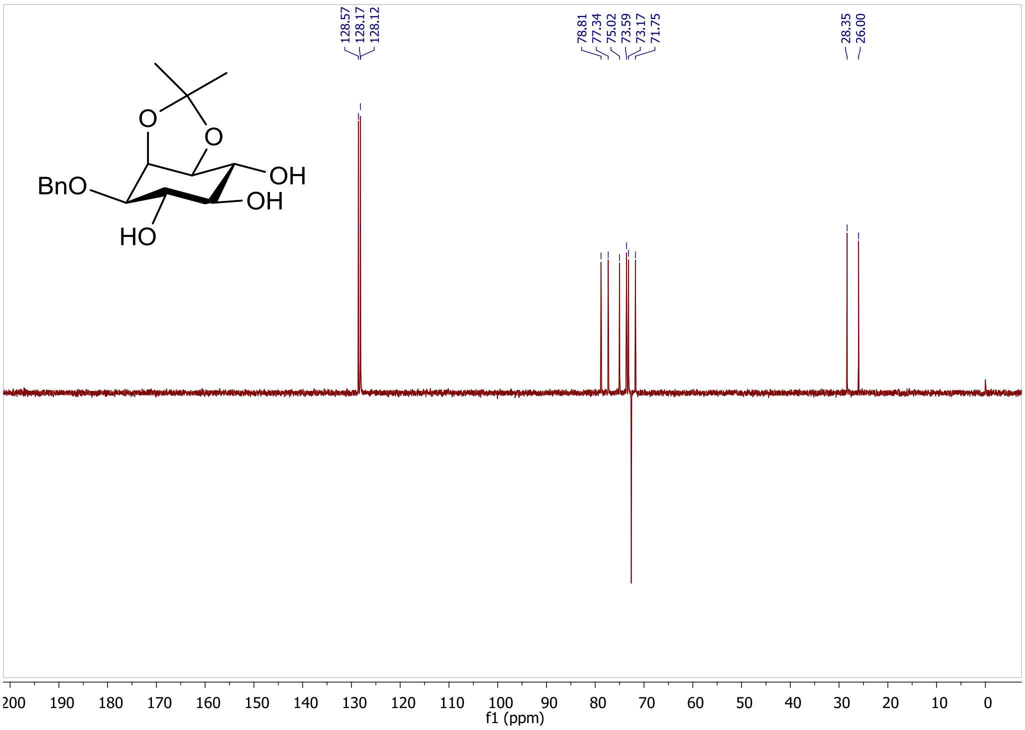

DL-3-*O*-benzyl-1,2-*O*-isopropylidene-*myo*-inositol (3): DEPT-135 NMR ($CDCl_3$, 100 MHz)

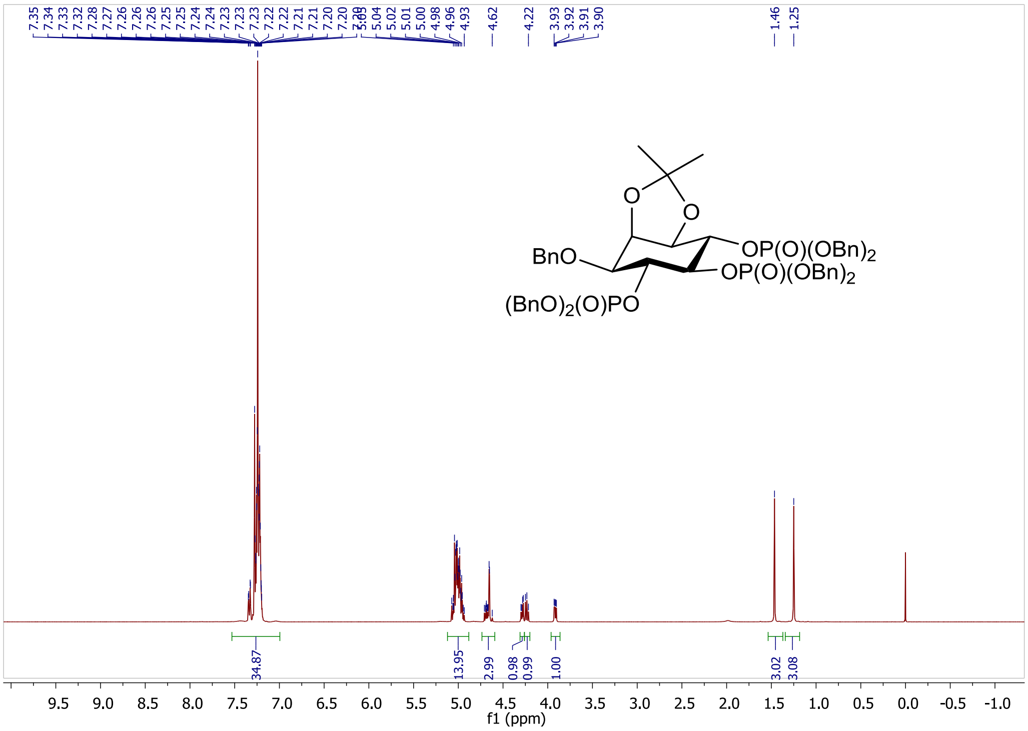

DL-3-*O*-benzyl-1,2-*O*-isopropylidene-*myo*-inositol 4,56-tris(dibenzylphosphate) (4): ^{1}**H NMR (** $CDCl_3$ **, 400 MHz)**

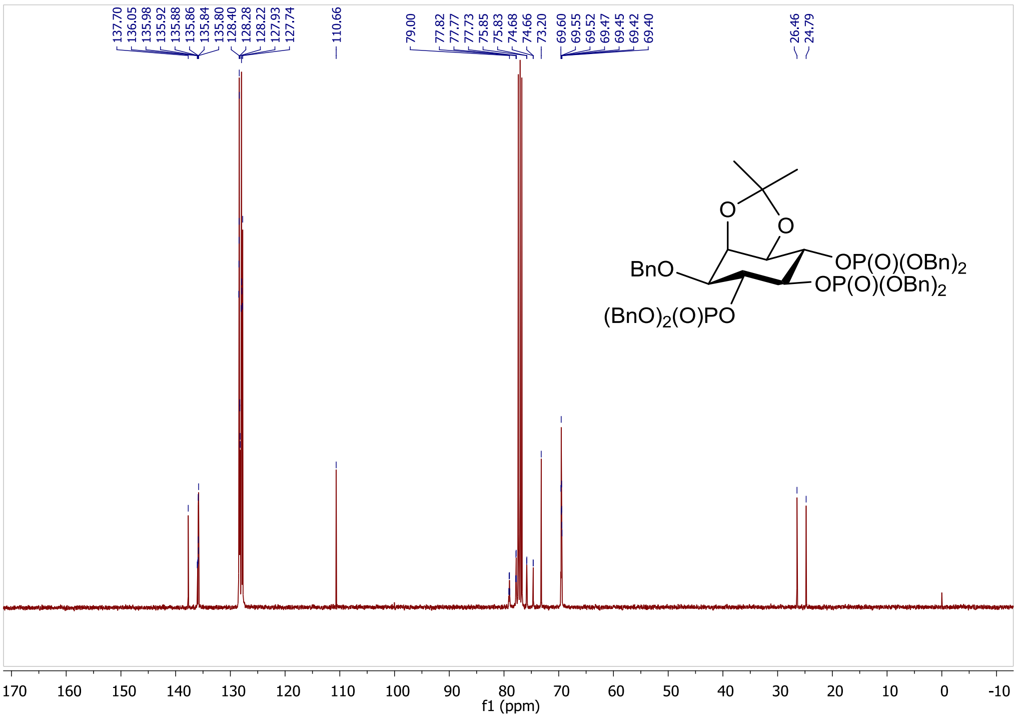

DL-3-*O*-benzyl-1,2-*O*-isopropylidene-*myo*-inositol 4,56-tris(dibenzylphosphate) (4): ^{13}C NMR (CDCl$_3$, 100 MHz)

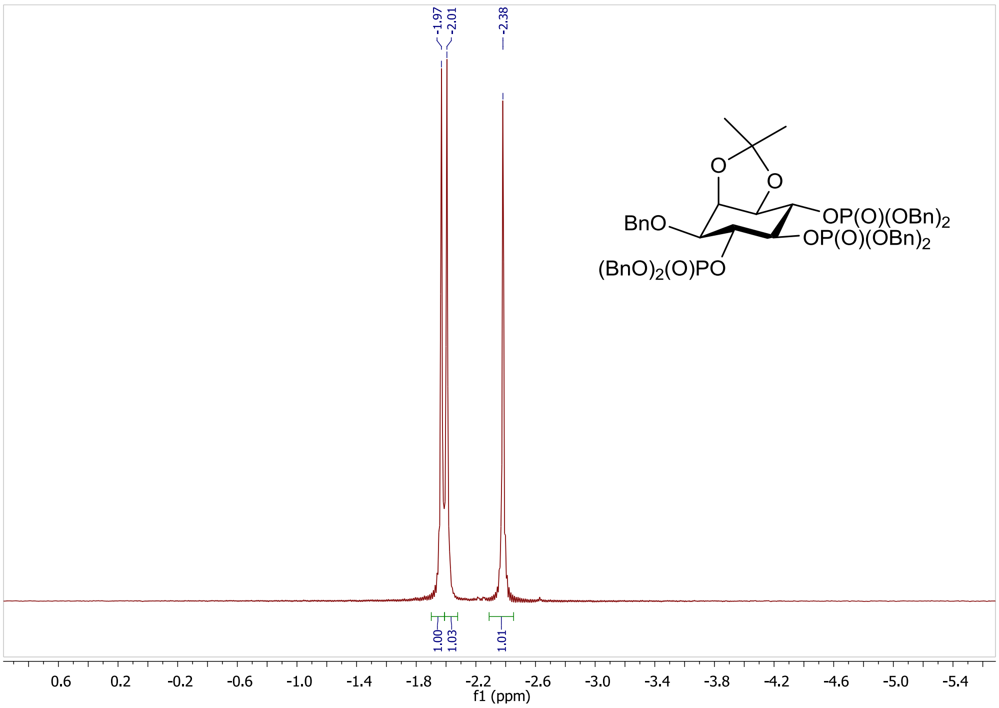

DL-3-*O*-benzyl-1,2-*O*-isopropylidene-*myo*-inositol 4,56-tris(dibenzylphosphate) (4):^{31}P NMR ($CDCl_3$, 162 MHz)

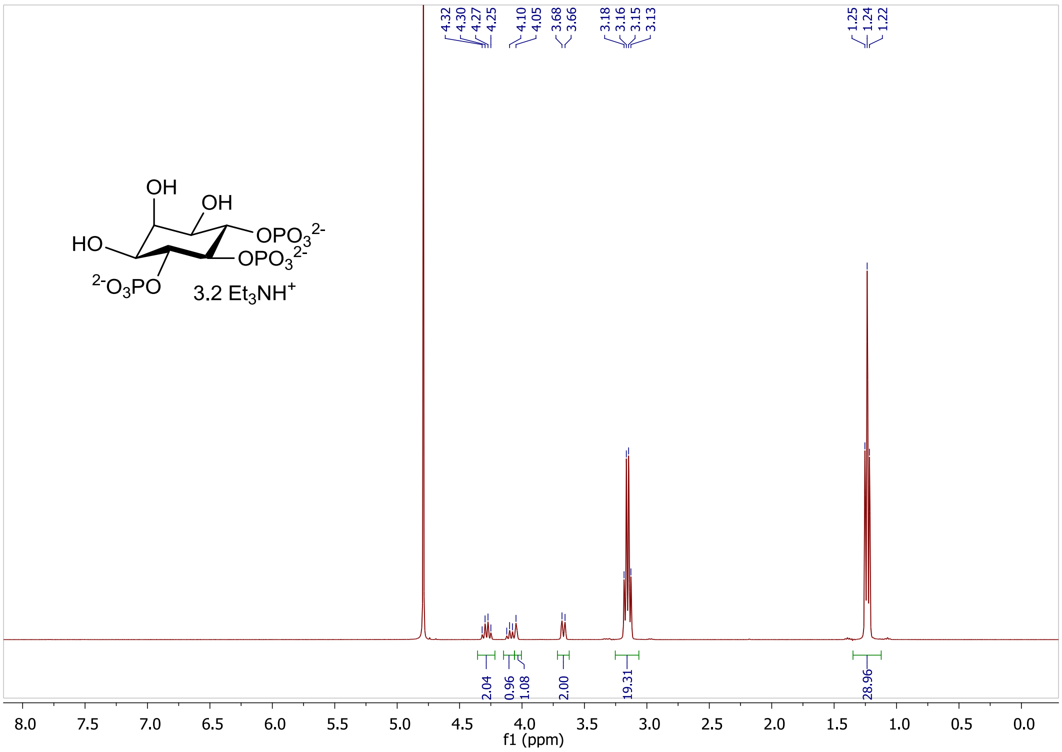

***myo*-Inositol 4,5,6-trisphosphate (1): ^{1}H NMR (D_2O, 400 MHz)**

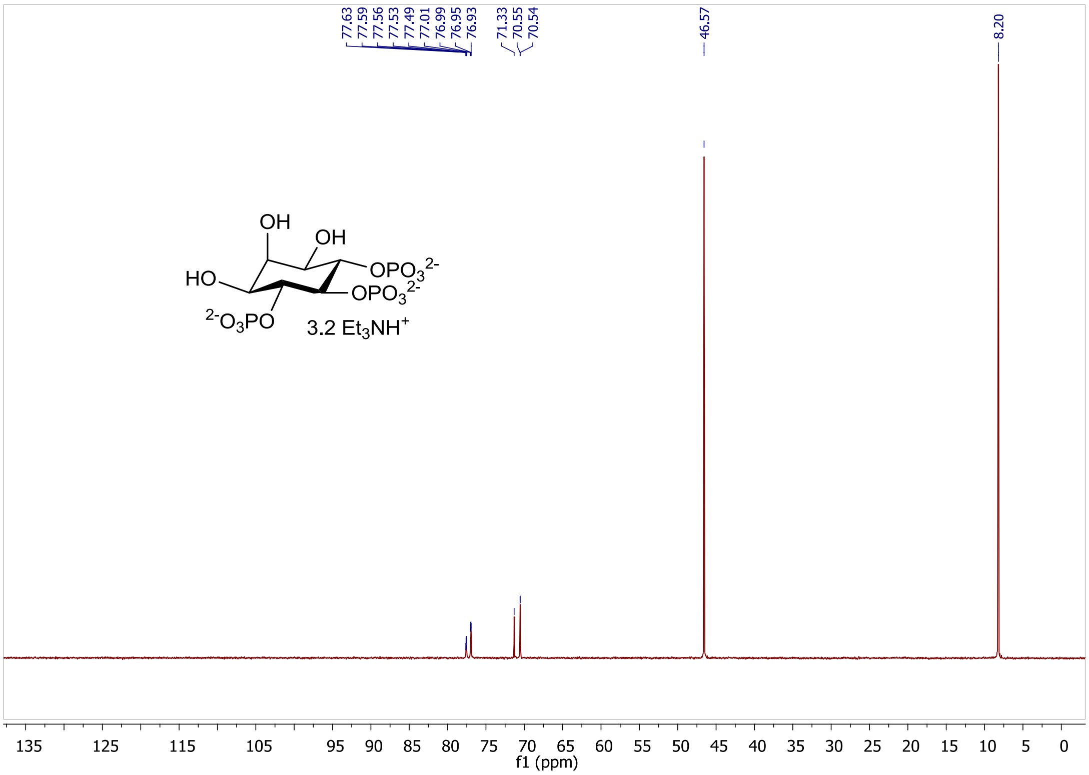

***myo*-Inositol 4,5,6-trisphosphate (1): ^{13}C NMR (D_2O, 100 MHz)**

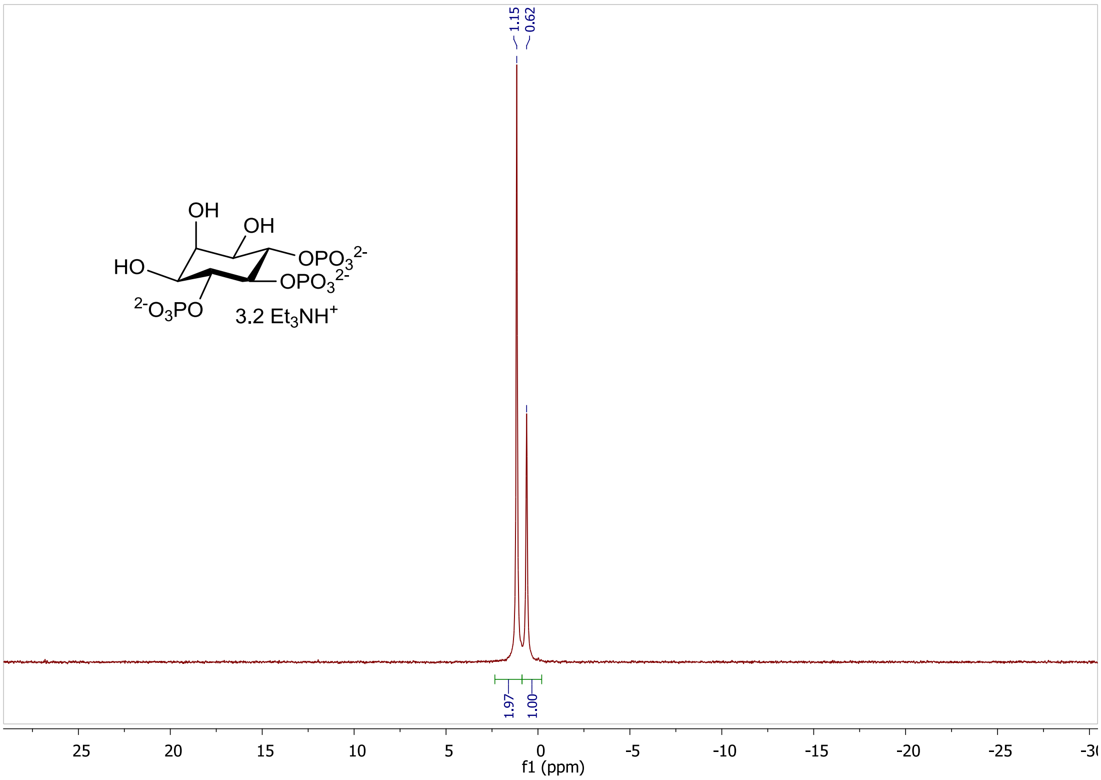

***myo*-Inositol 4,5,6-trisphosphate (1): ^{31}P NMR (D2O, 162 MHz)**

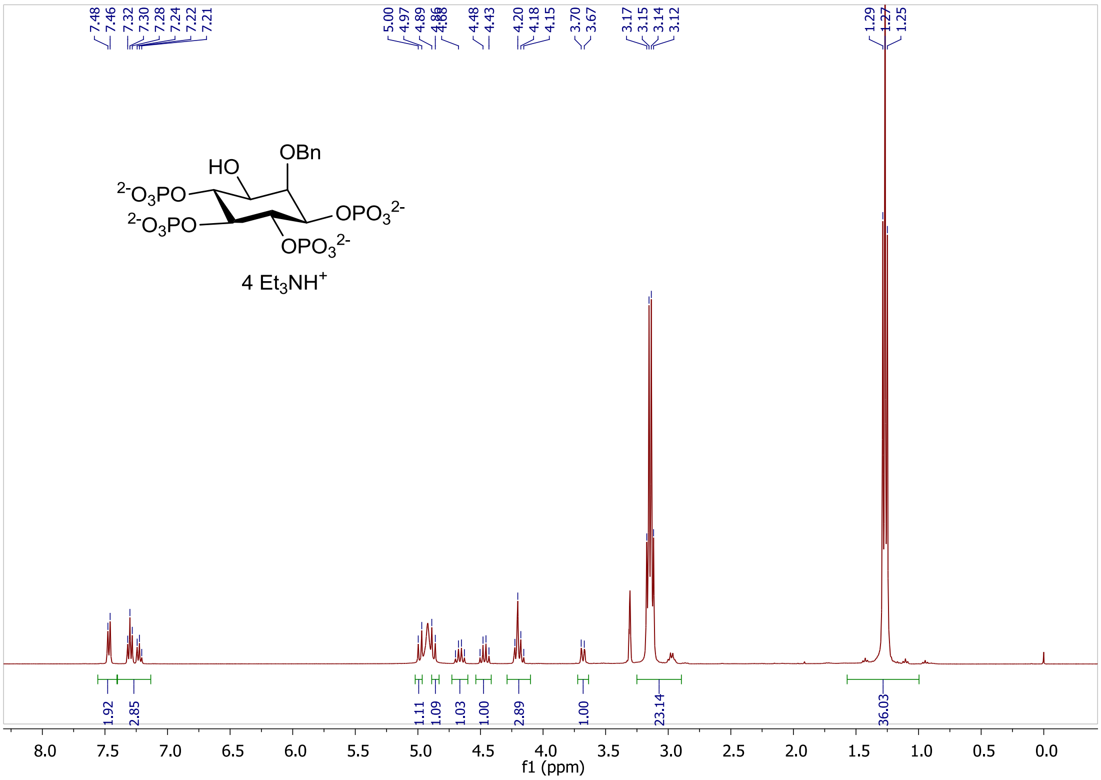

D-2-O-Benzyl-myo-inositol 1,4,5,6-tetrakisphosphate (5): ^{1}H NMR (CD_3OD, 400 MHz)

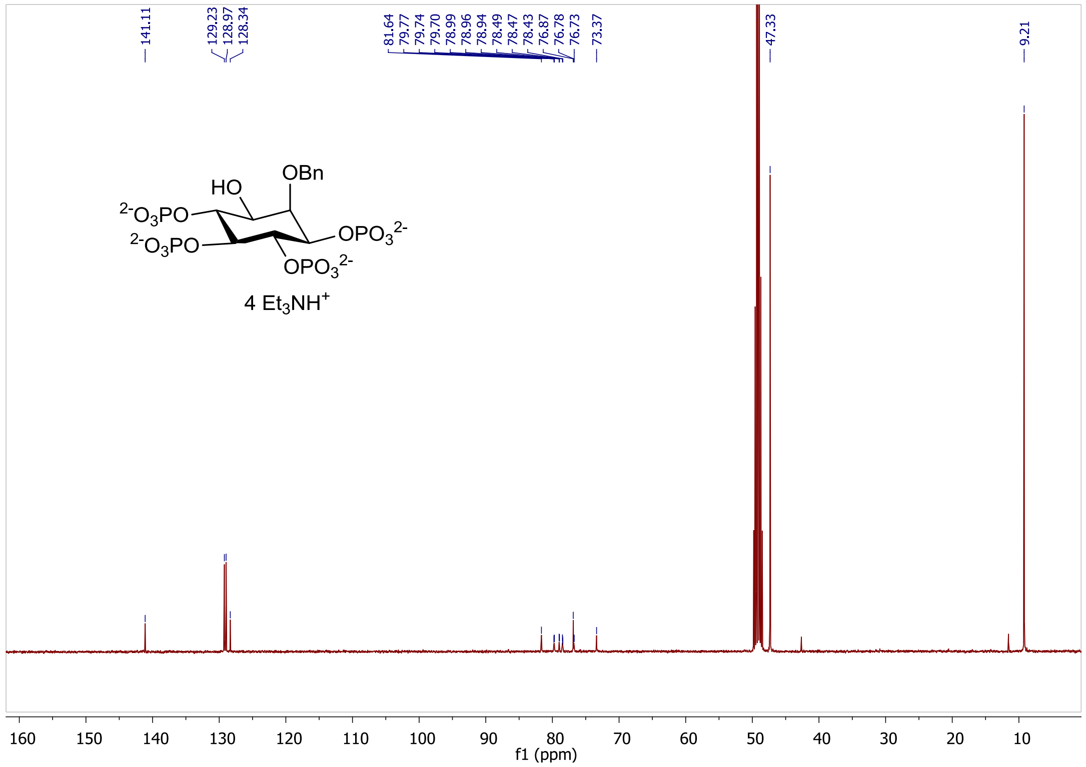

D-2-*O*-Benzyl-*myo*-inositol 1,4,5,6-tetrakisphosphate (5): ^{13}C NMR (CD_3OD, 100 MHz)

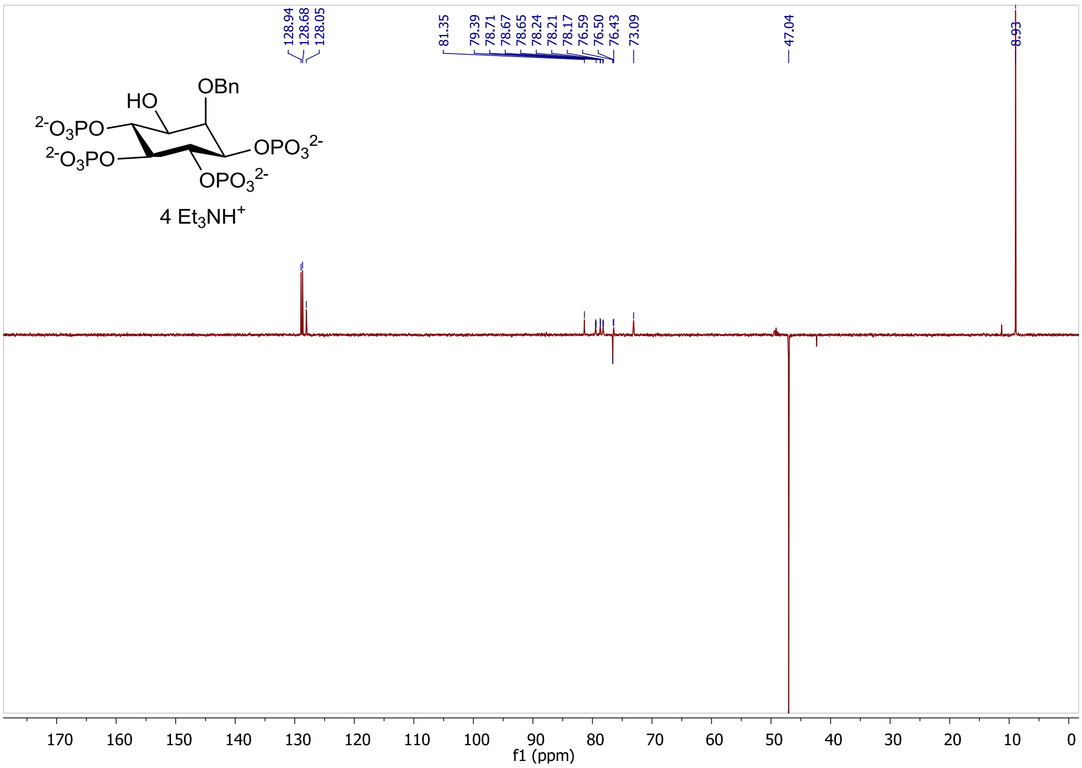

D-2-O-Benzyl-*myo*-inositol 1,4,5,6-tetrakisphosphate (5): DEPT-135 NMR (CD3OD, 100 MHz)

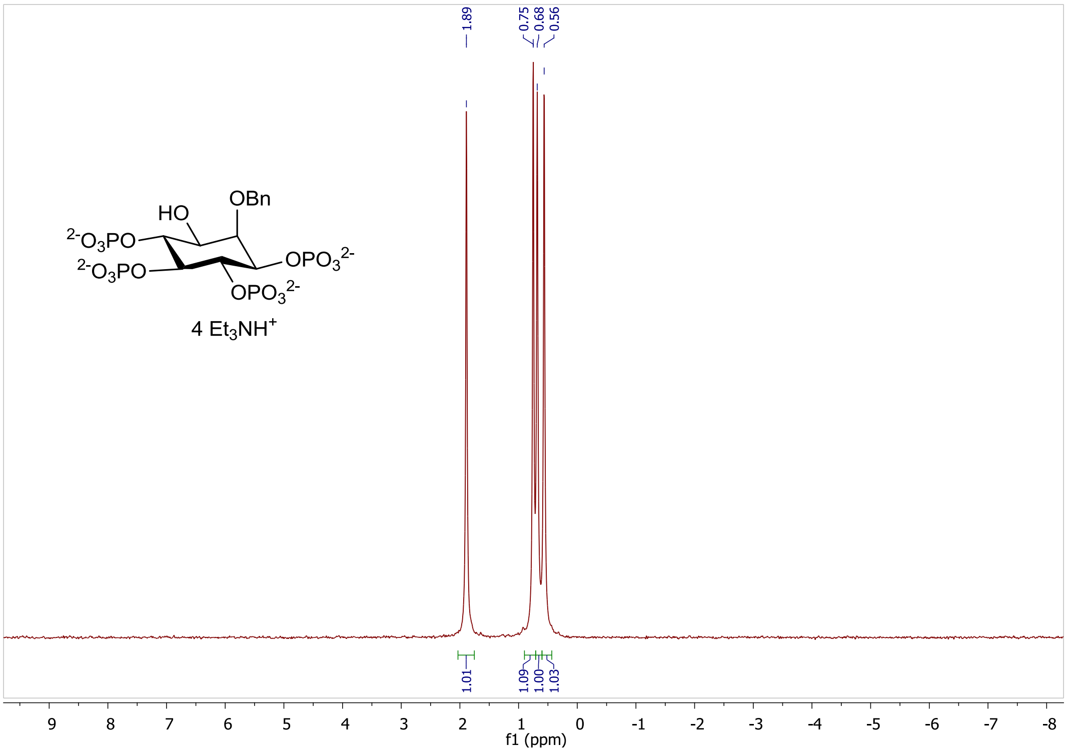

D-2-*O*-Benzyl-*myo*-inositol 1,4,5,6-tetrakisphosphate (5): ^{31}P NMR (CD_3OD, 162 MHz)

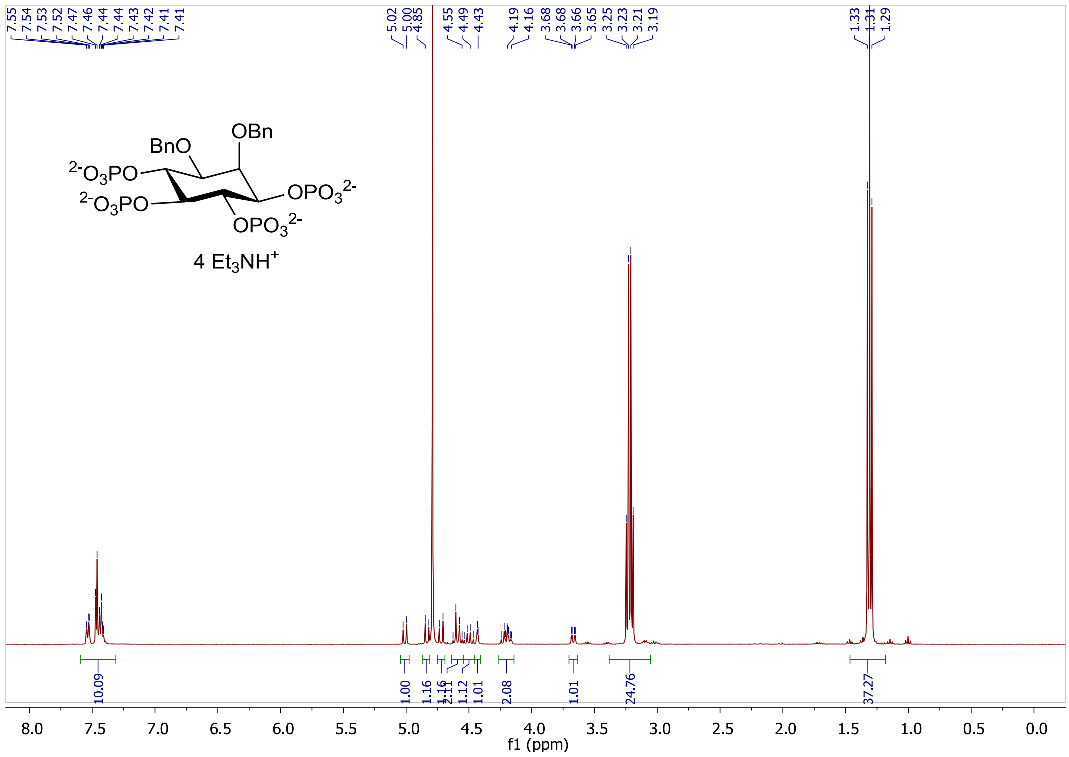

D-2,3-di-*O*-benzyl-*myo*-inositol 1,4,5,6-tetrakisphosphate (6): ^{1}H NMR (D_2O, 400 MHz)

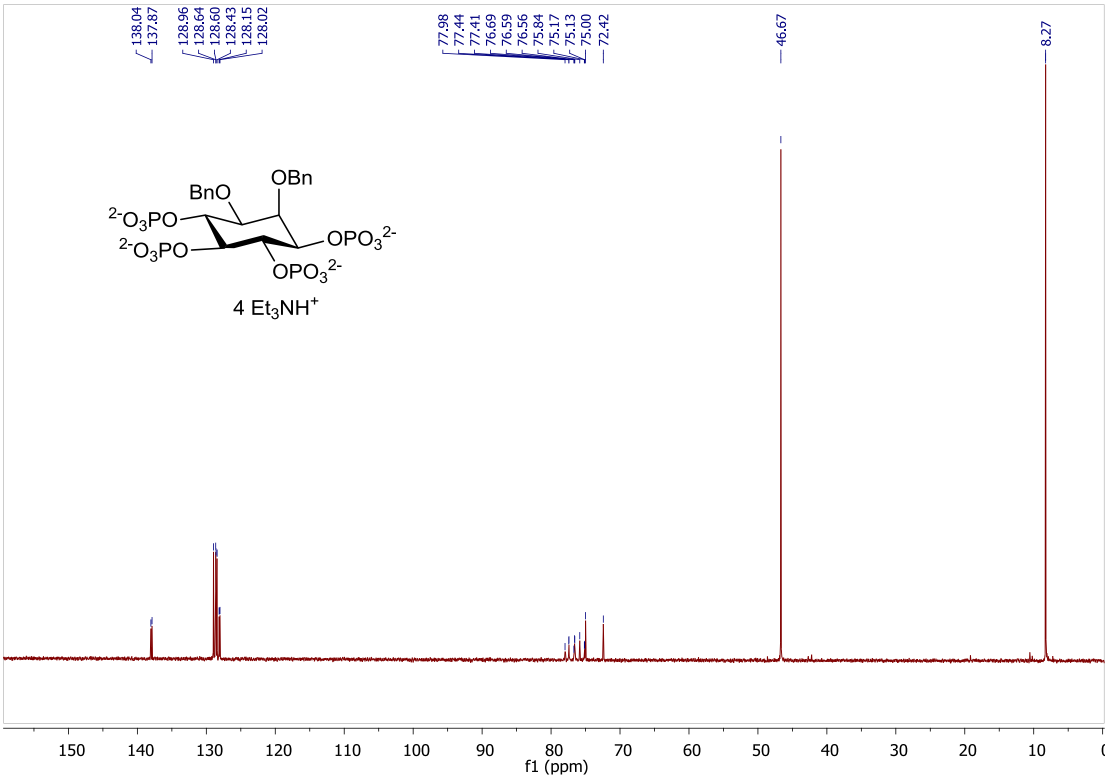

D-2,3-di-*O*-benzyl-*myo*-inositol 1,4,5,6-tetrakisphosphate (6): ^{13}C NMR (D_2O, 100 MHz)

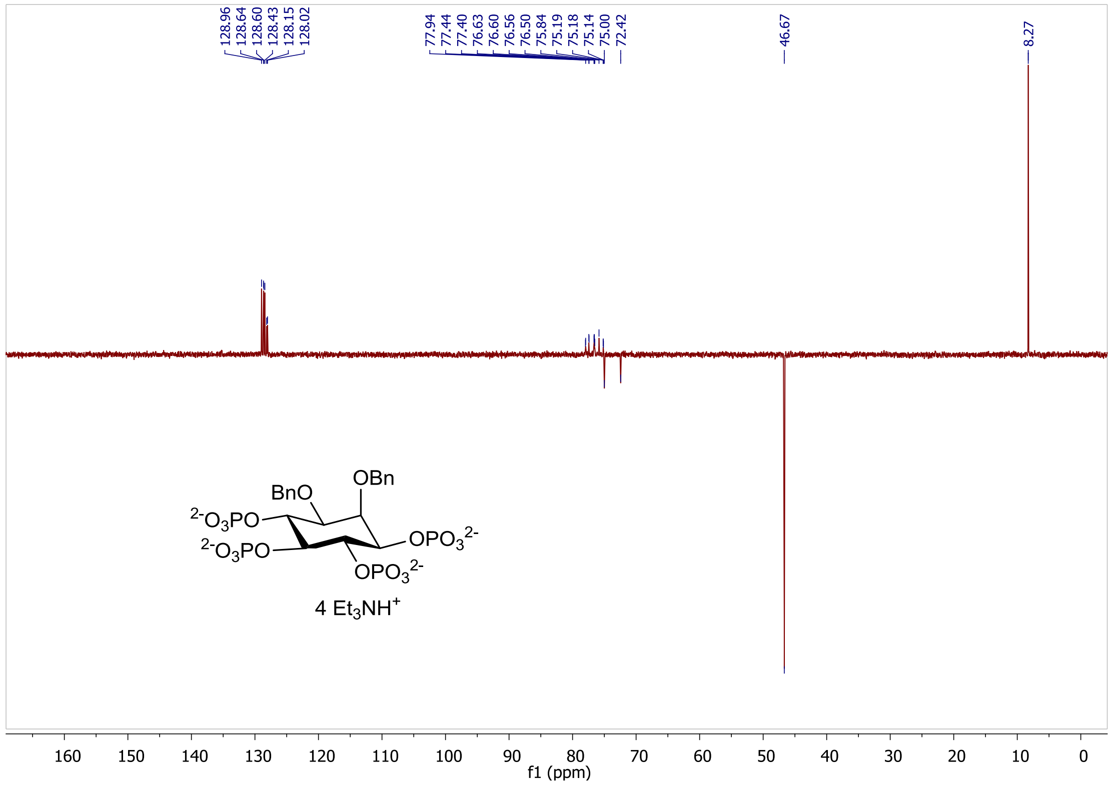

D-2,3-di-*O*-benzyl-*myo*-inositol 1,4,5,6-tetrakisphosphate (6): DEPT-135 NMR (D_2O, 100 MHz)

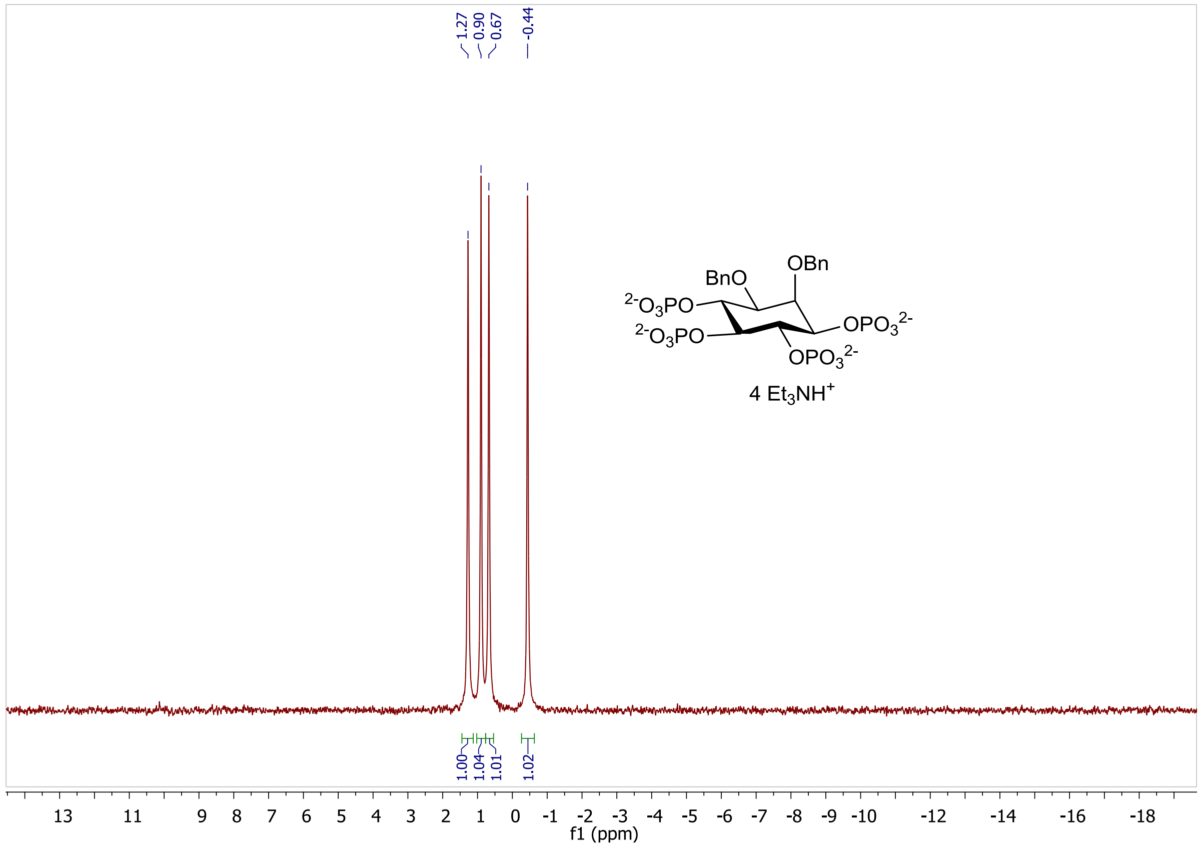

D-2,3-di-*O*-benzyl-*myo*-inositol 1,4,5,6-tetrakisphosphate (6): ^{31}P NMR (CD_3OD, 162 MHz)

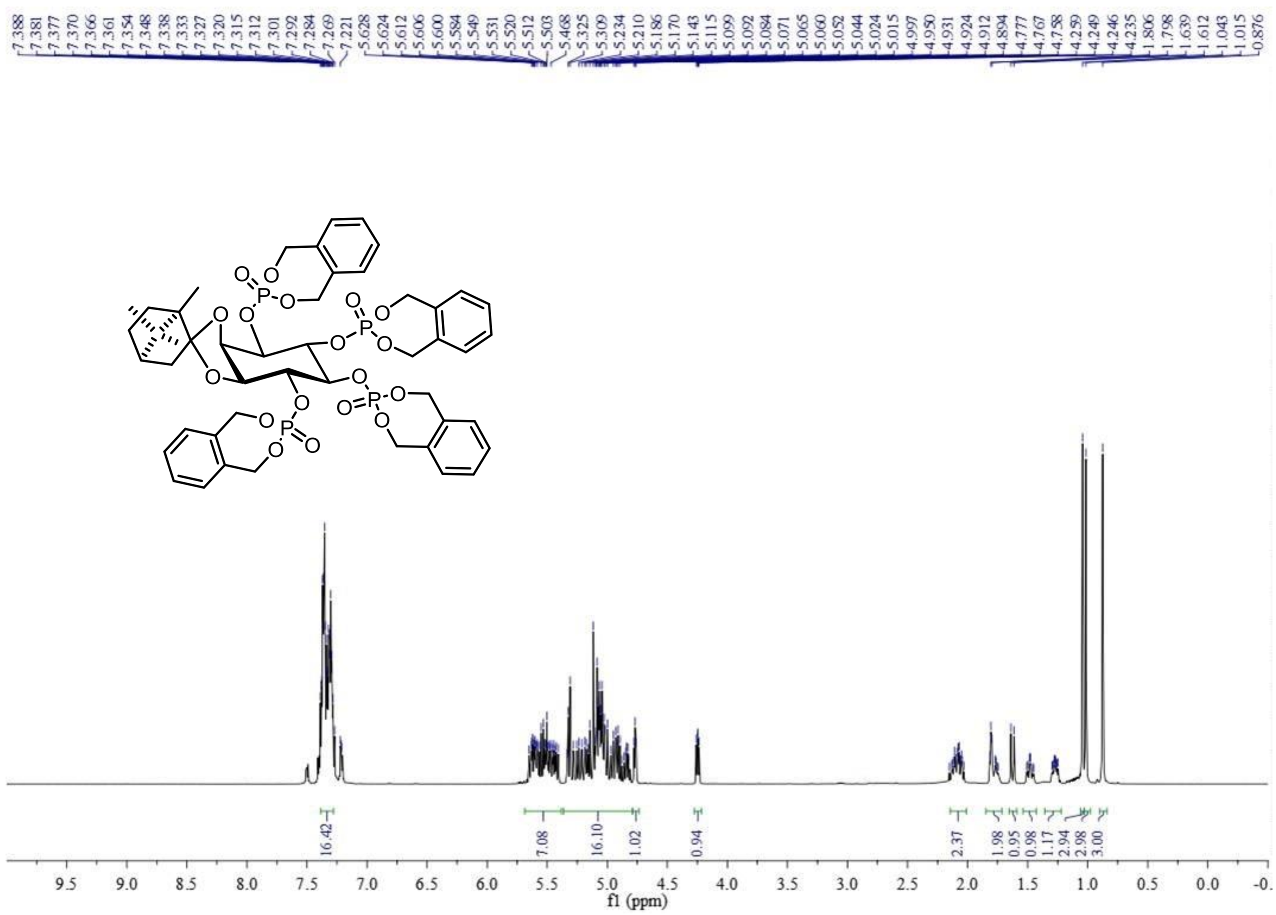

D-1,4,5,6-Tetra-*O*-(2-oxo-5.6-benzo-1,3,2-dioxaphosphep-2-yl)-1,2-*O*-(D-1,7,7-trimethyl-[2.2.l]bicyclohept-2-ylidene)-*myo*-inositol (11) : ^{1}H NMR ($CDCl_3$, 500 MHz)

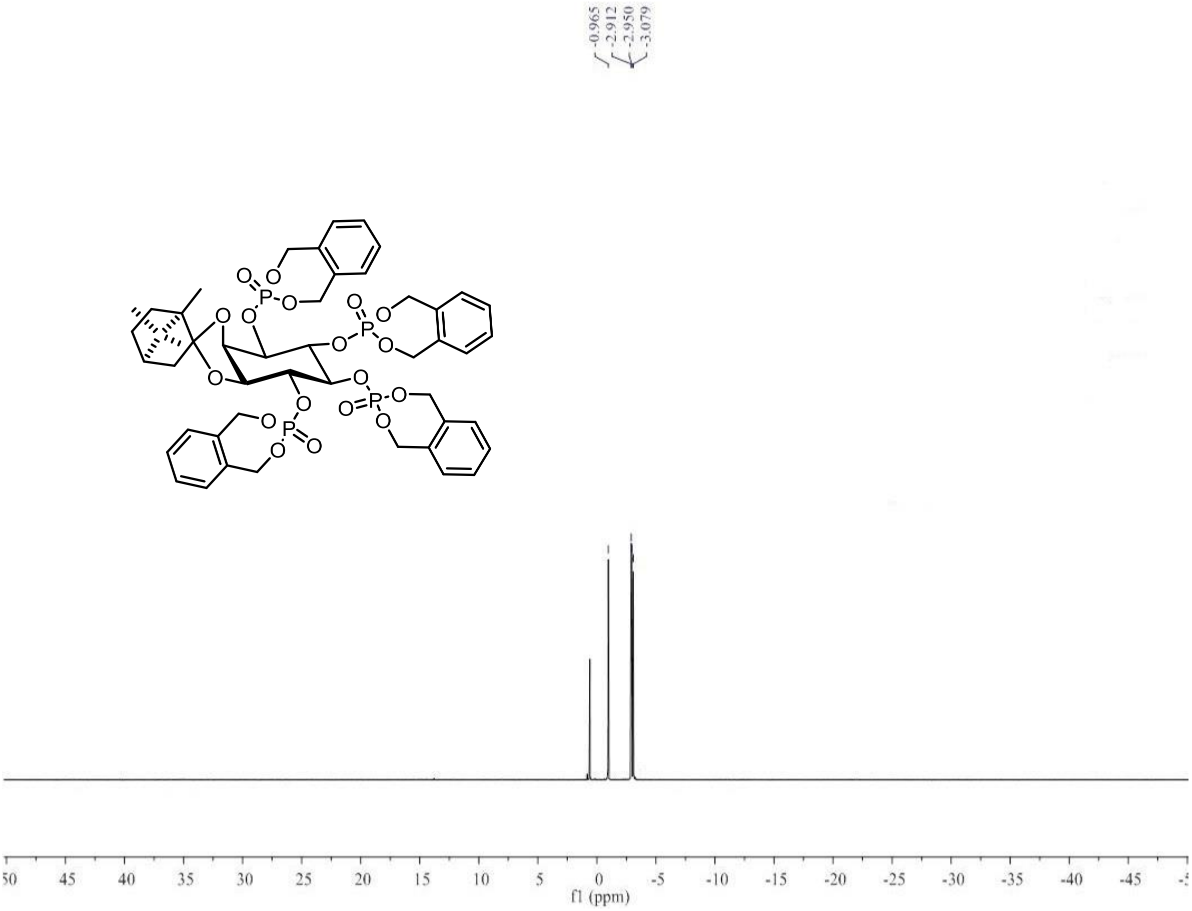

D-1,4,5,6-Tetra-*O*-(2-oxo-5.6-benzo-1,3,2-dioxaphosphep-2-yl)-1,2-*O*-(D-1,7,7-trimethyl-[2.2.l]bicyclohept-2-ylidene)-*myo*-inositol (11) : ^{31}P NMR ($CDCl_3$, 202 MHz)

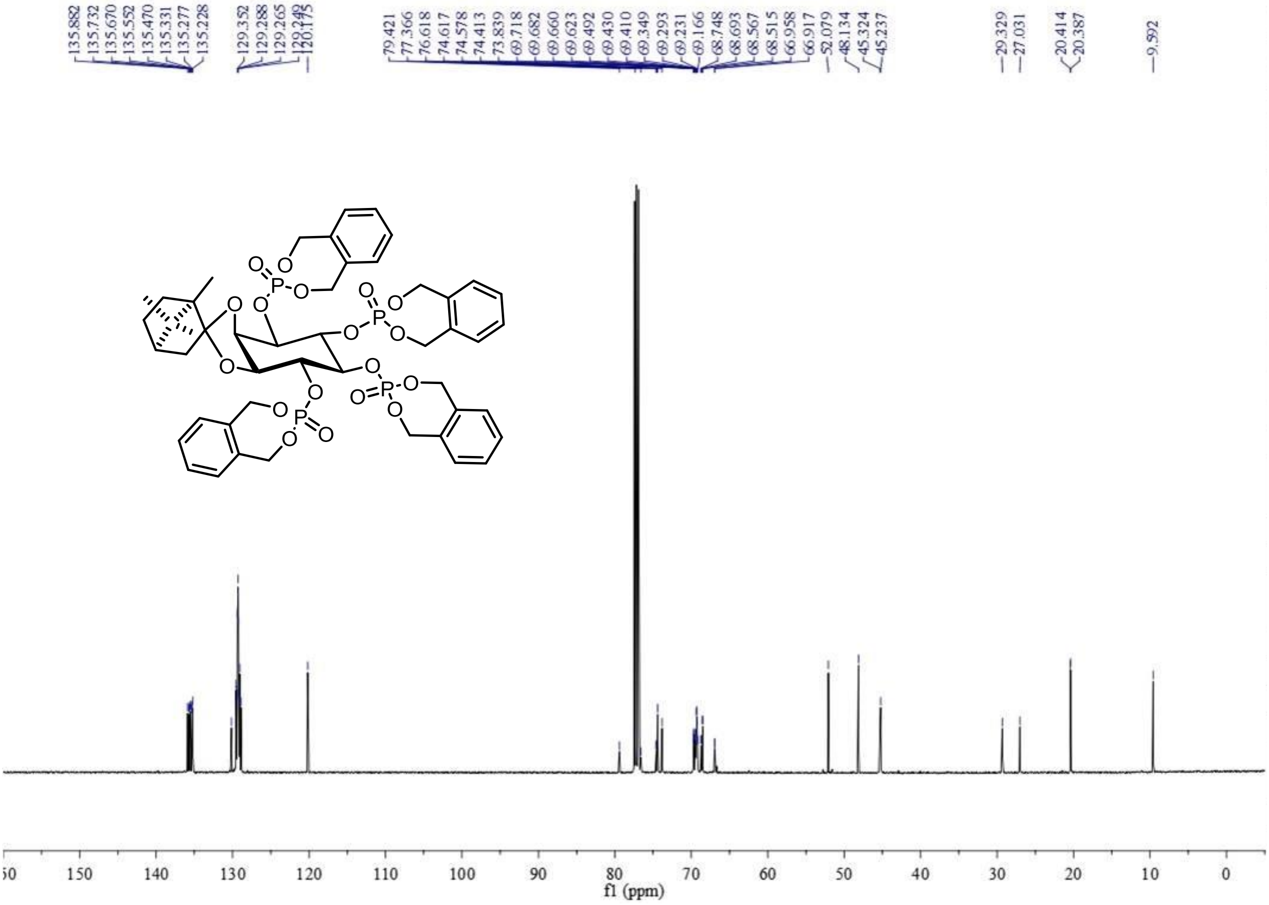

D-1,4,5,6-Tetra-*O*-(2-oxo-5.6-benzo-1,3,2-dioxaphosphep-2-yl)-1,2-*O*-(D-1,7,7-trimethyl-[2.2.l]bicyclohept-2-ylidene)-*myo*-inositol (11) : ^{13}C NMR ($CDCl_3$, 126 MHz)

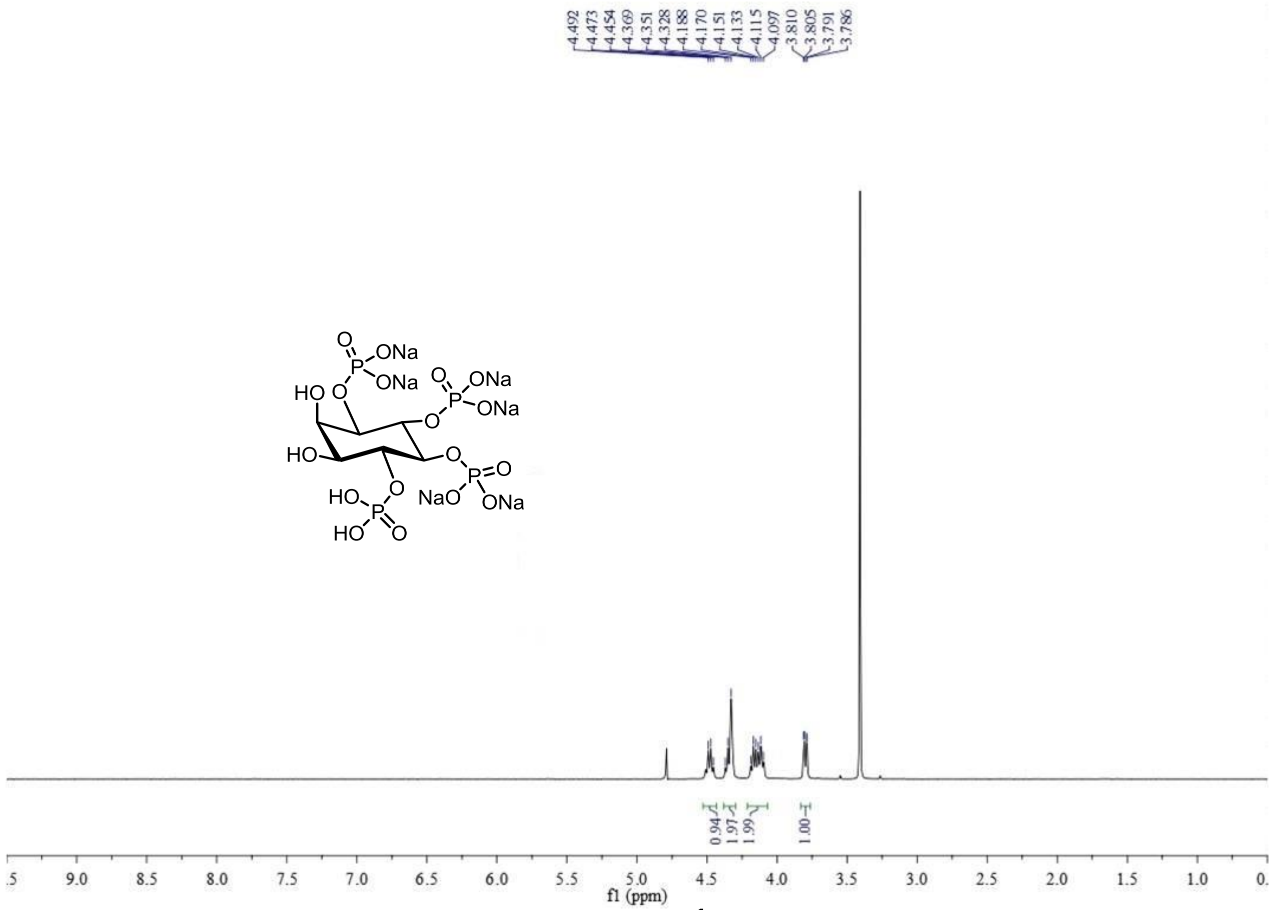

D-*myo*-Inositol 1,4,5,6-tetrakisphosphate (8) : ^{1}H NMR (D_2O, 500 MHz)

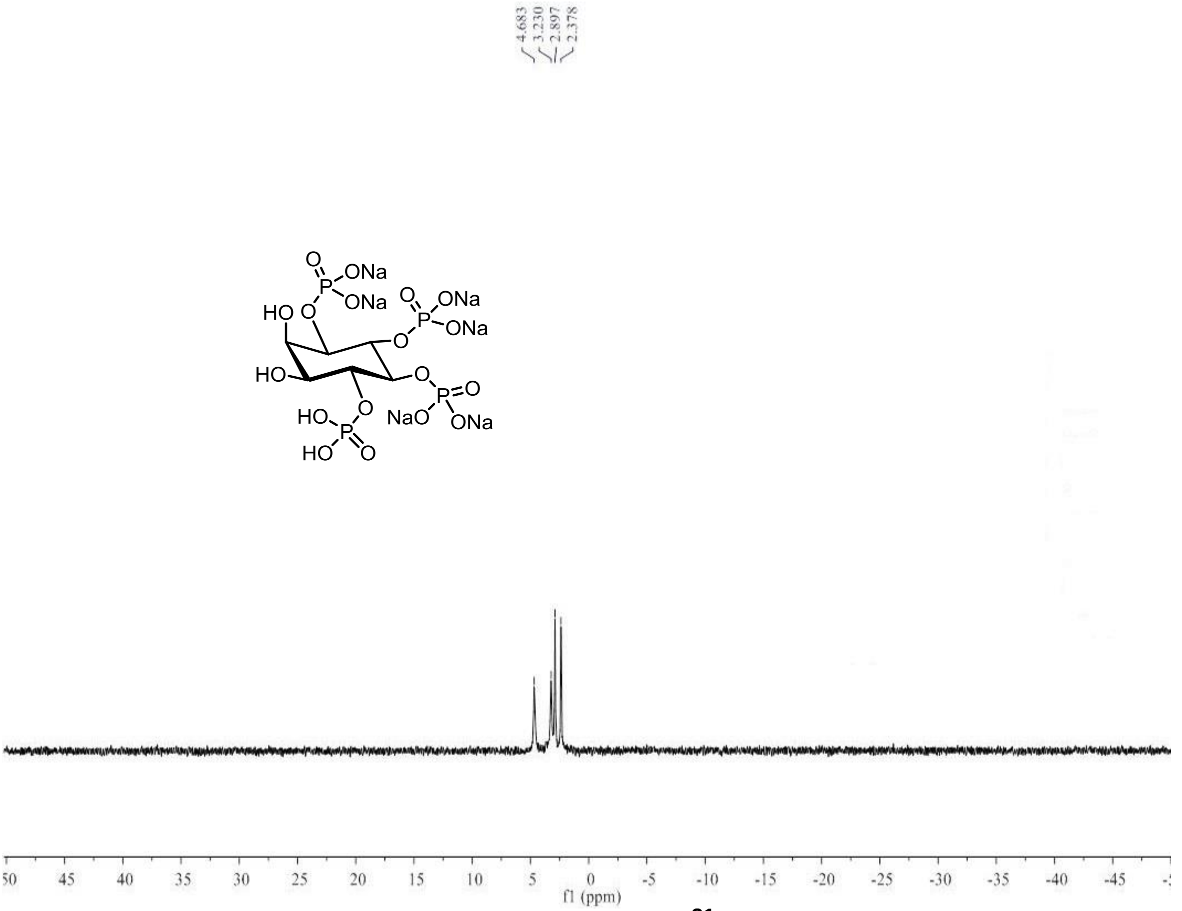

D-*myo*-Inositol 1,4,5,6-tetrakisphosphate (8) : ^{31}P NMR (D_2O, 202 MHz)

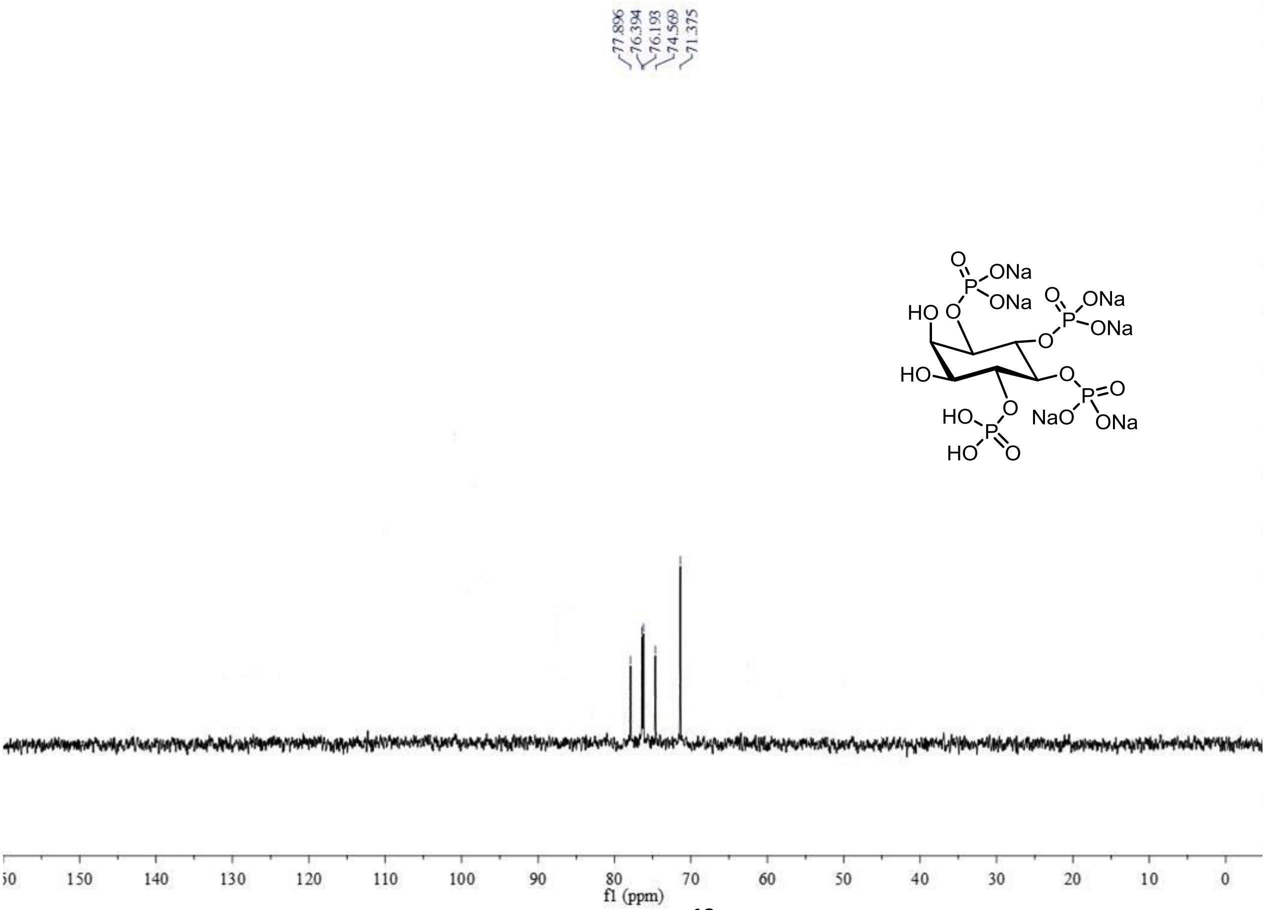

D-*myo*-Inositol 1,4,5,6-tetrakisphosphate (8) : ^{13}C NMR (D_2O, 126 MHz)

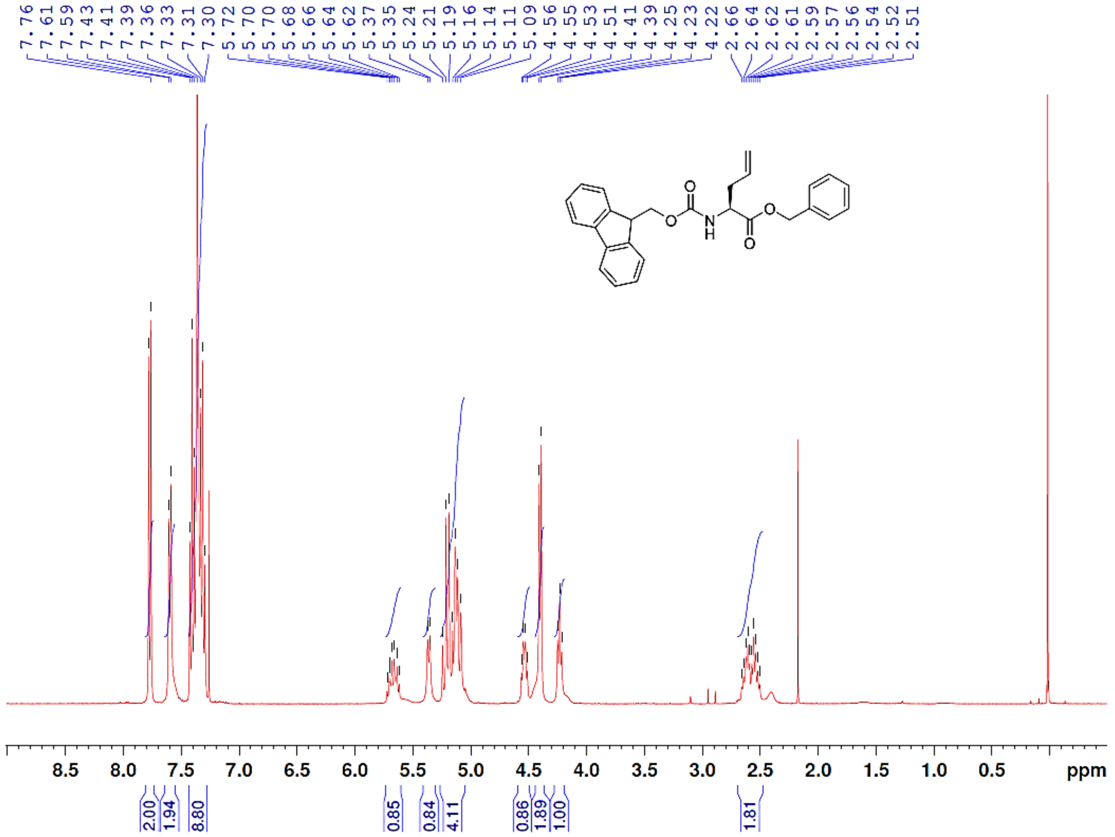

Fmoc-L-allylglycine-OBn (13): ^{1}H NMR ($CDCl_3$, 300 MHz)

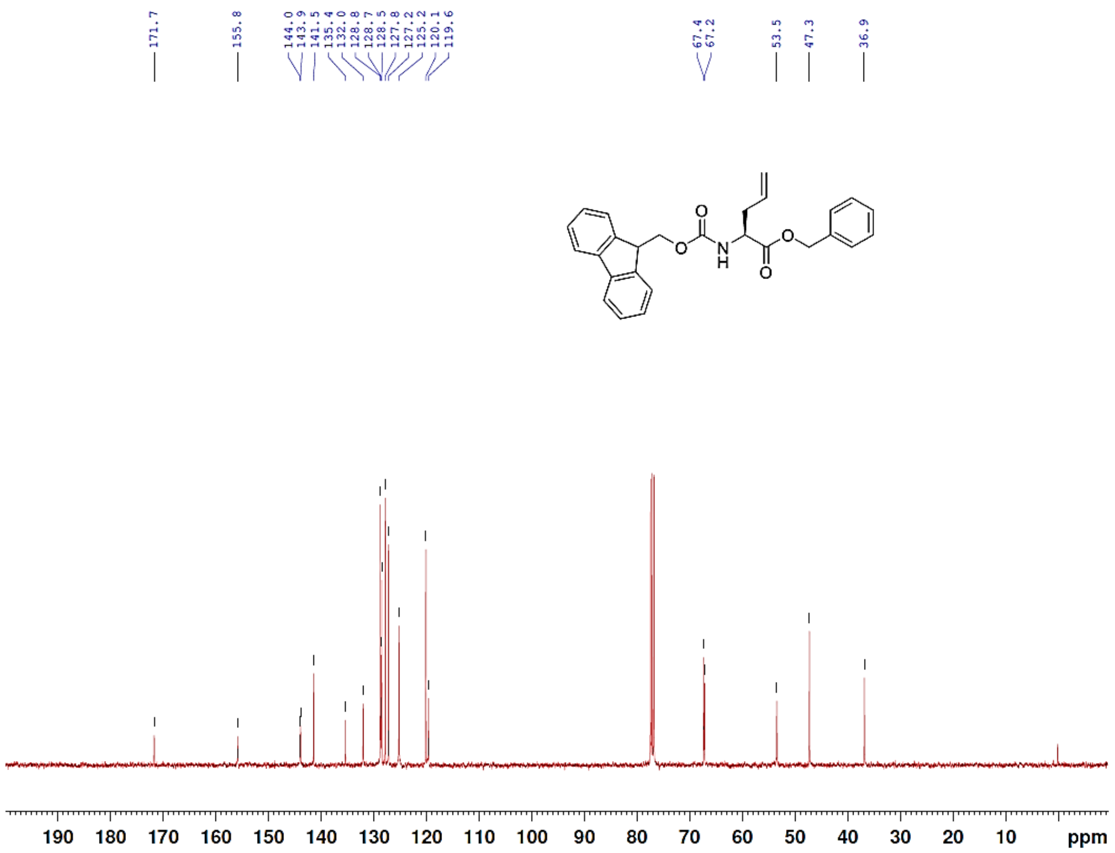

Fmoc-L-allylglycine-OBn (13): ^{13}C NMR ($CDCl_3$, 100 MHz)

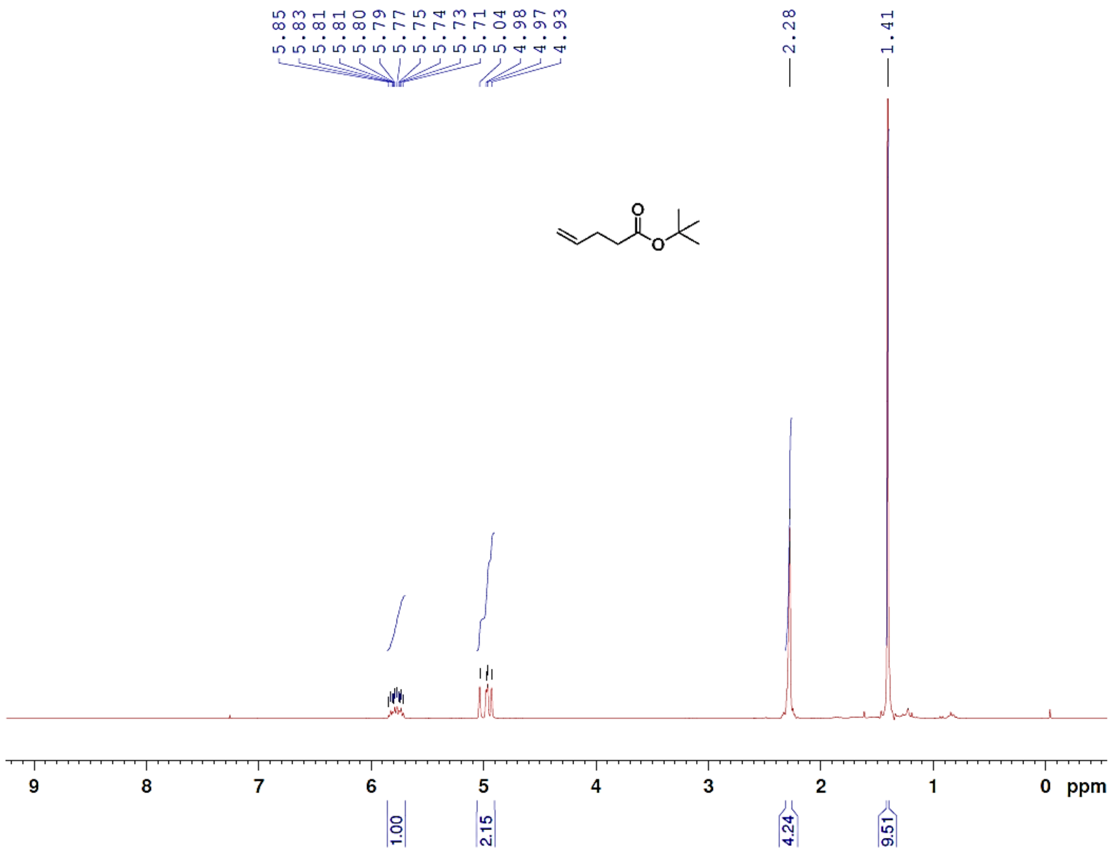

***t*-Butyl pent-4-enoate (12): ^{1}H NMR ($CDCl_3$, 300 MHz).**

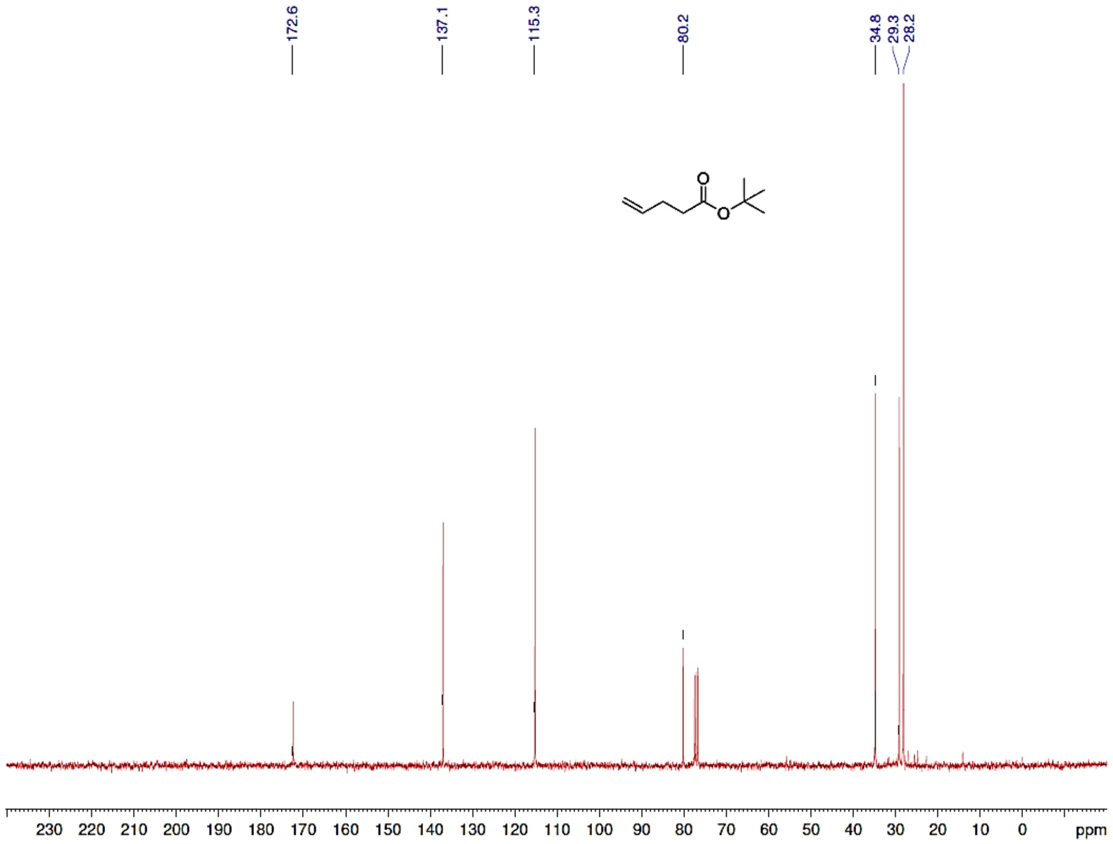

***t*-Butyl pent-4-enoate (12): ^{13}C NMR ($CDCl_3$, 75 MHz).**

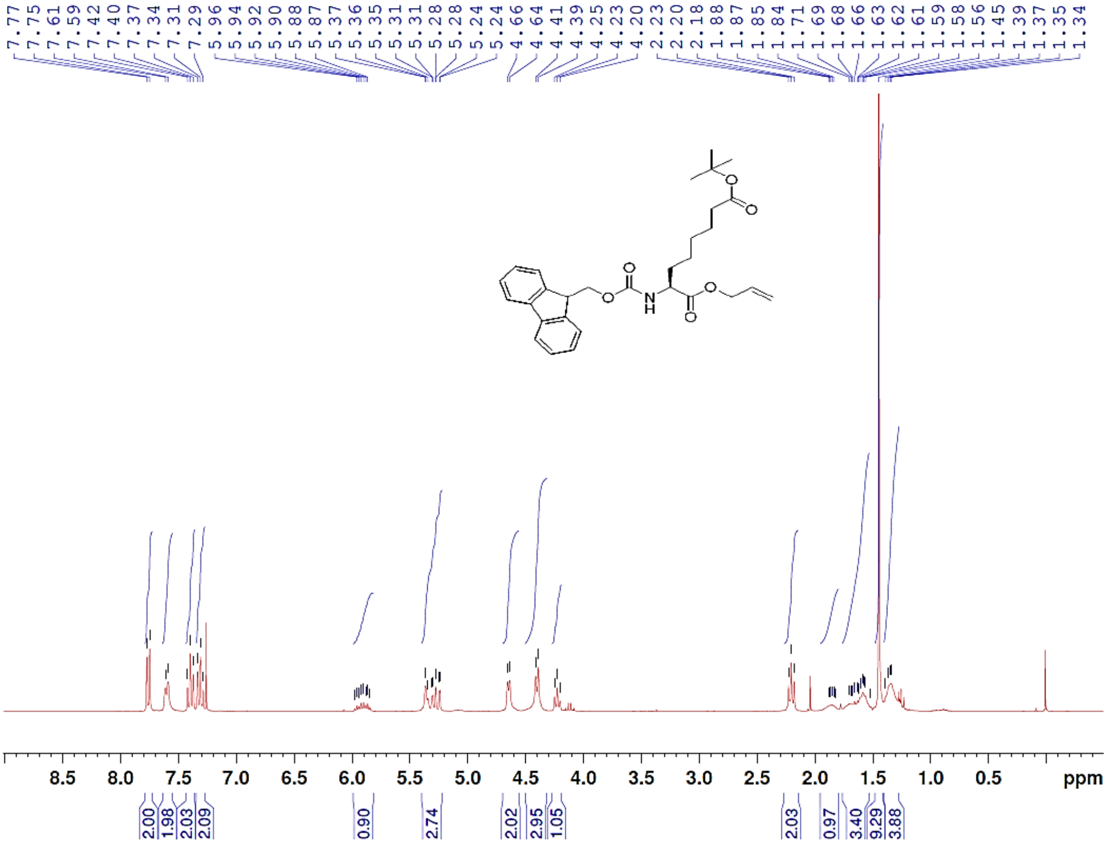

Fmoc-octanoate(*t*Bu)-OAllyl (14): ^{1}H NMR ($CDCl_3$, 300 MHz).

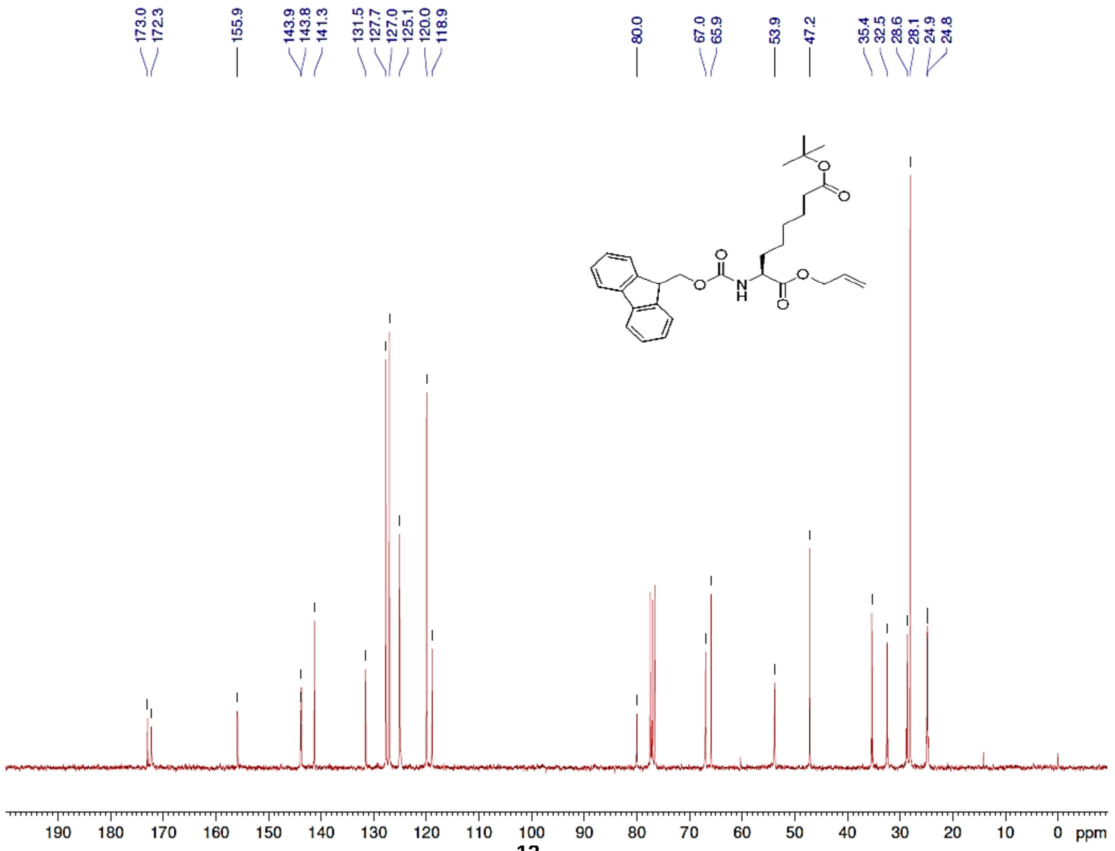

Fmoc-octanoate(*t*Bu)-OAllyl (14): ^{13}C NMR ($CDCl_3$, 75 MHz).

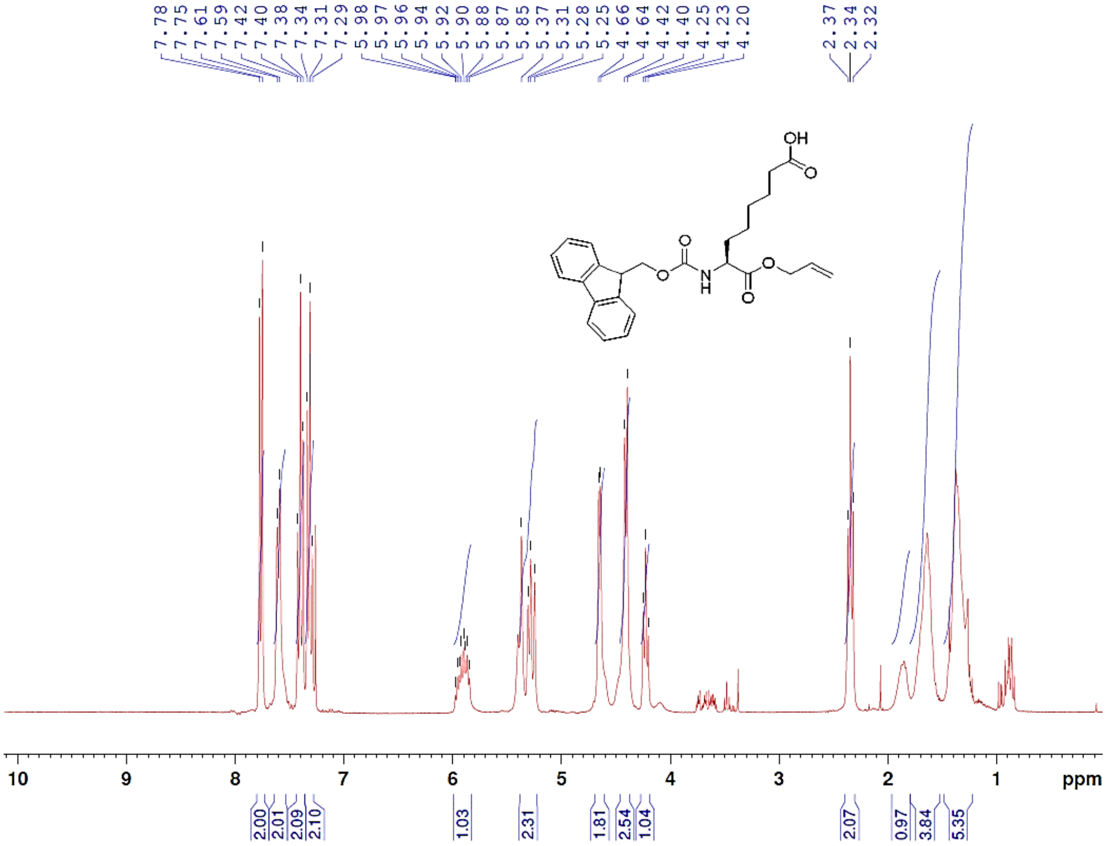

Fmoc-oxooctanoic acid-OAllyl (15): ^{1}H NMR ($CDCl_3$, 300 MHz).

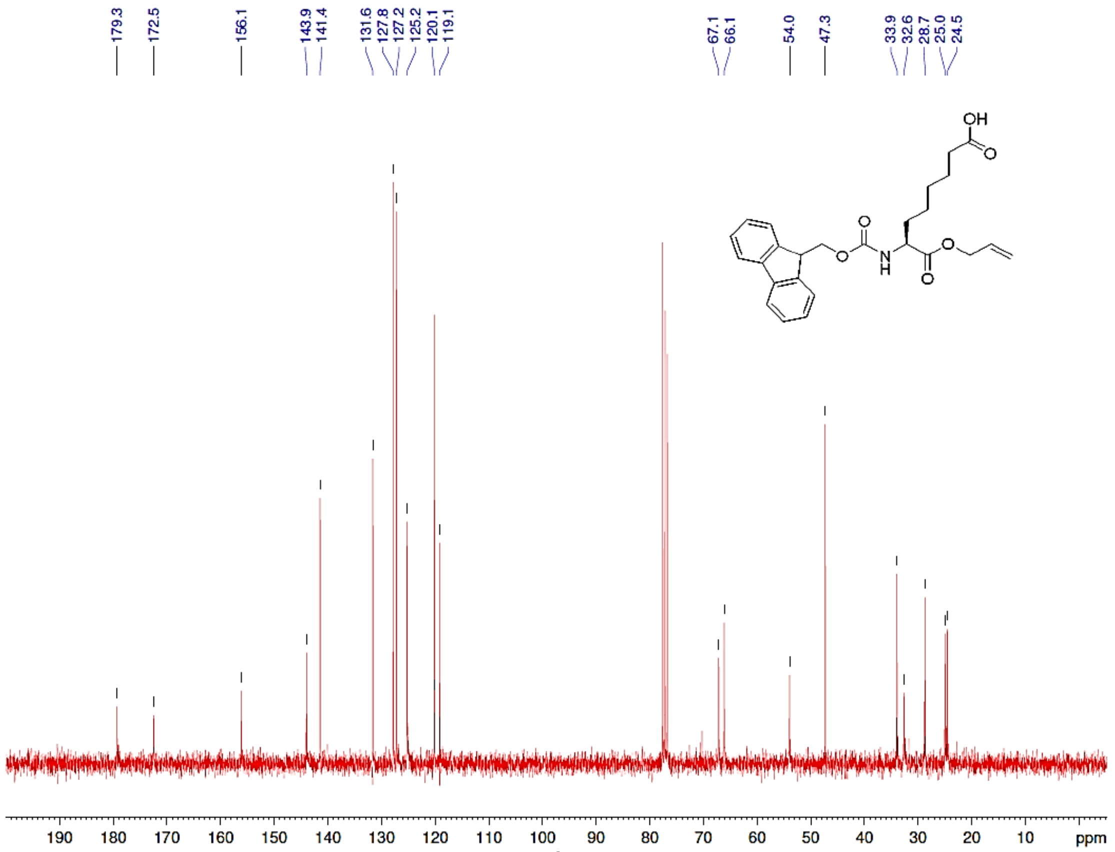

Fmoc-oxooctanoic acid-OAllyl (15): ^{1}H NMR ($CDCl_3$, 300 MHz).

HPLC SPECTRA

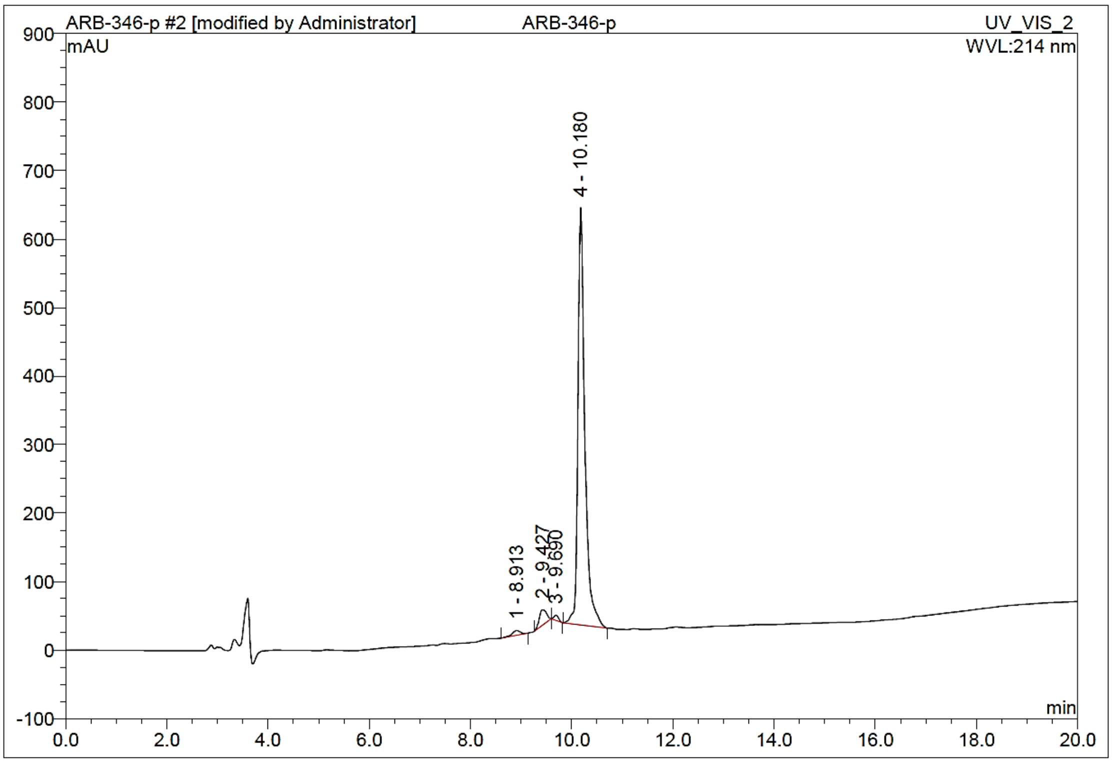

Ac-Lys-Gly-Gly-Ala-Hao-Arg-His-NH_2: analytical HPLC 0-20% acetonitrile/H_2O (0.1% trifluoroacetic acid), 15 min gradient.

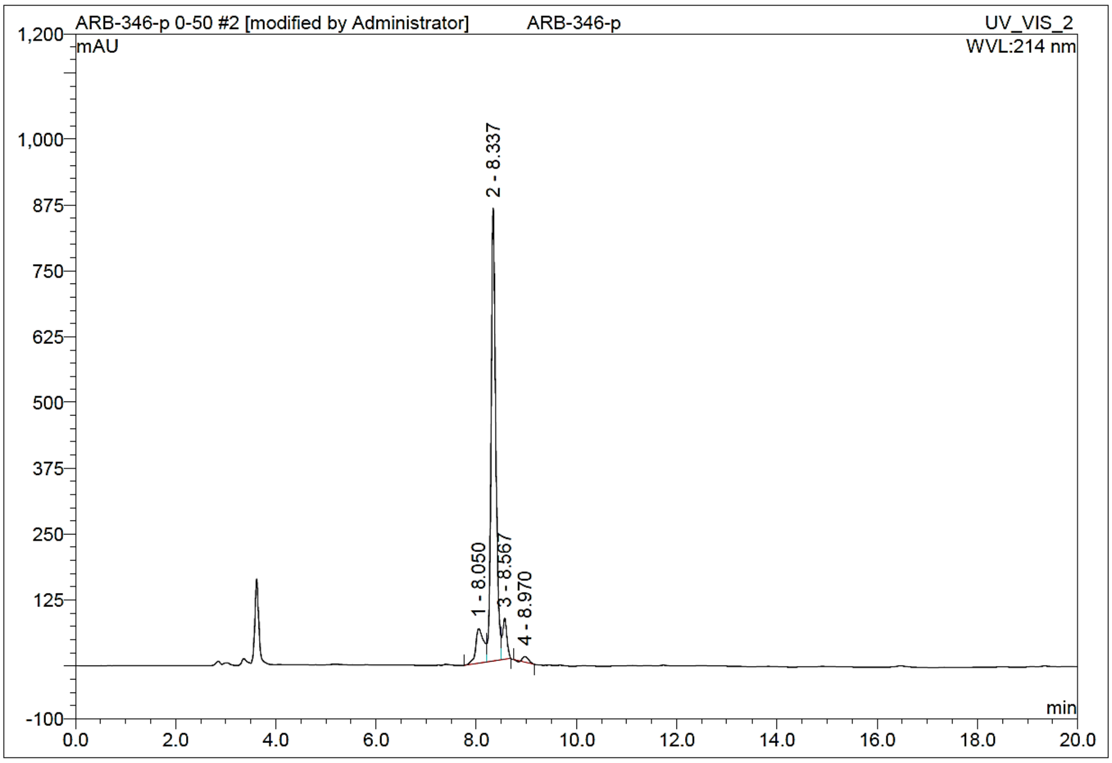

Ac-Lys-Gly-Gly-Ala-Hao-Arg-His-NH$_2$: analytical HPLC 0-50% acetonitrile/H_2O (0.1% trifluoroacetic acid), 15 min gradient.

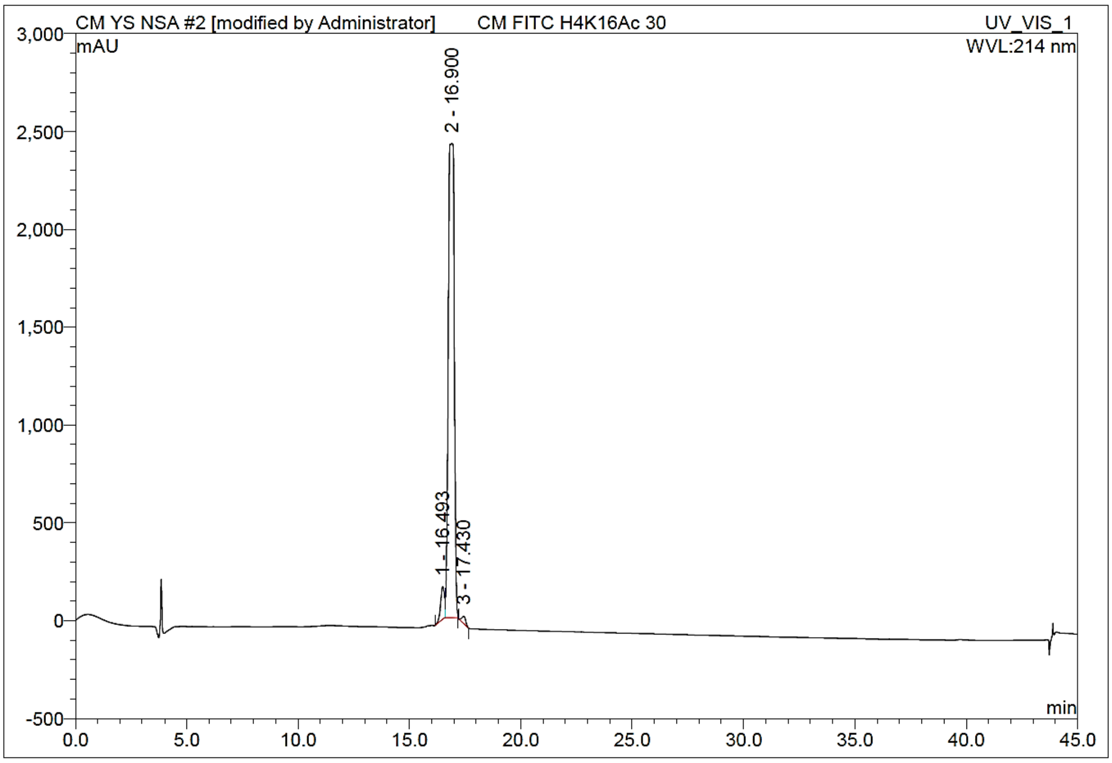

FITC-Ahx-Lys-Gly-Gly-Ala-Hao-Arg-His-NH_2: analytical HPLC 0-100% acetonitrile/H2O (0.1% trifluoroacetic acid), 30 min gradient.

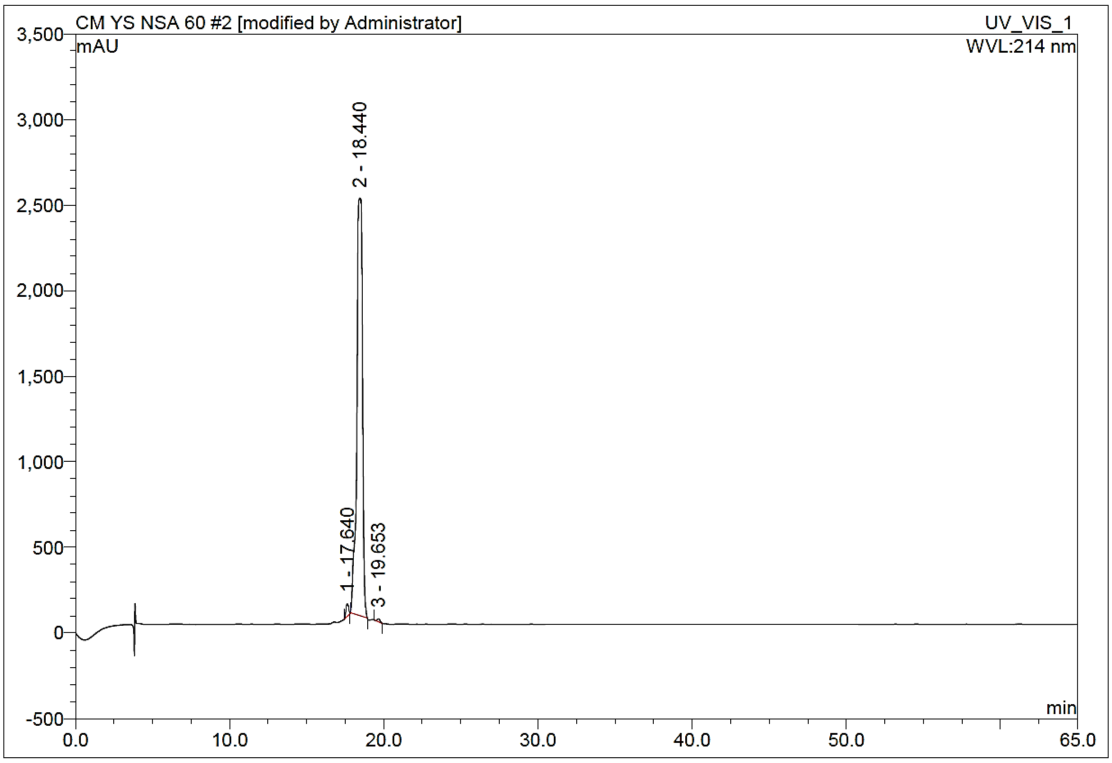

FITC-Ahx-Lys-Gly-Gly-Ala-Hao-Arg-His-NH$_2$: analytical HPLC 0-100% acetonitrile/H2O (0.1% trifluoroacetic acid), 60 min gradient.

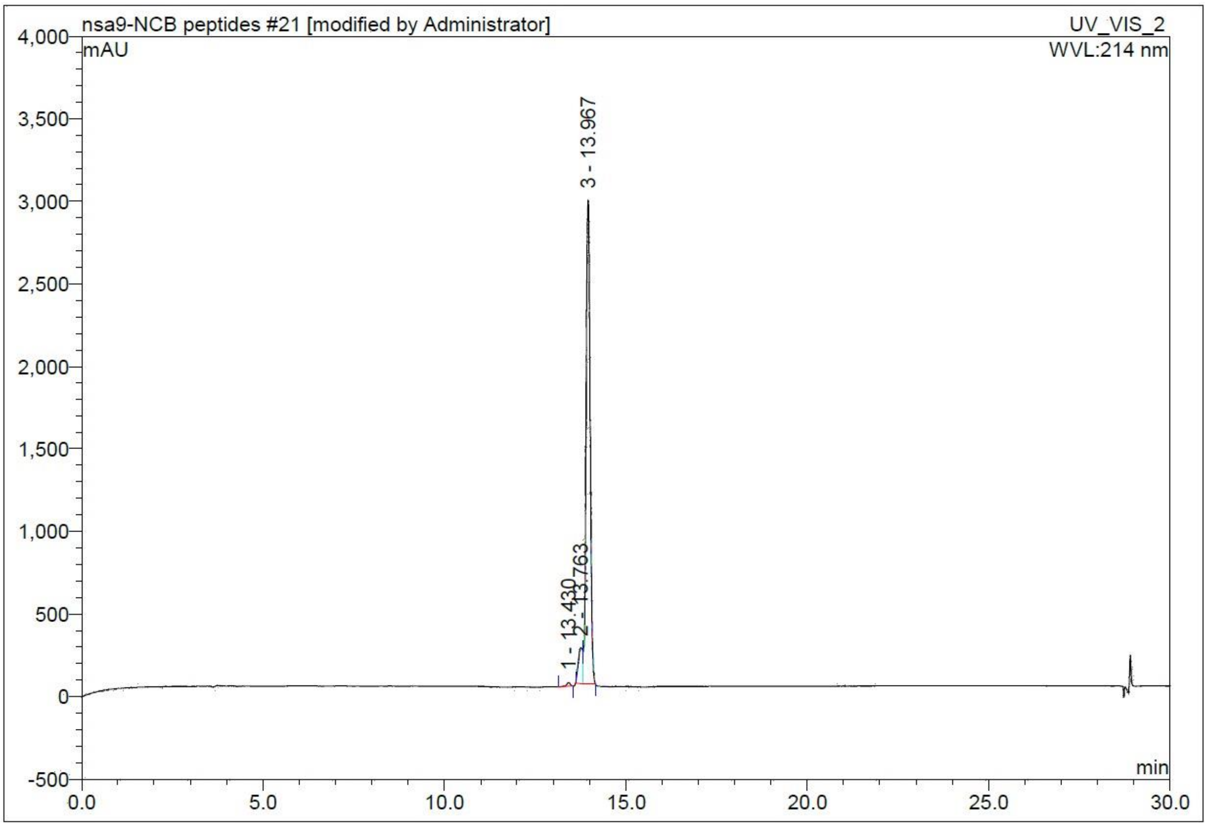

FITC-Ahx-Ser-Glu-Arg-Gly-Lys(Ac)Gly-Gly-Lys-NH$_2$ (H4(1-8)K5Ac): analytical HPLC 5-100% acetonitrile/H$_2$O (0.1% trifluoroacetic acid), 15 min gradient.

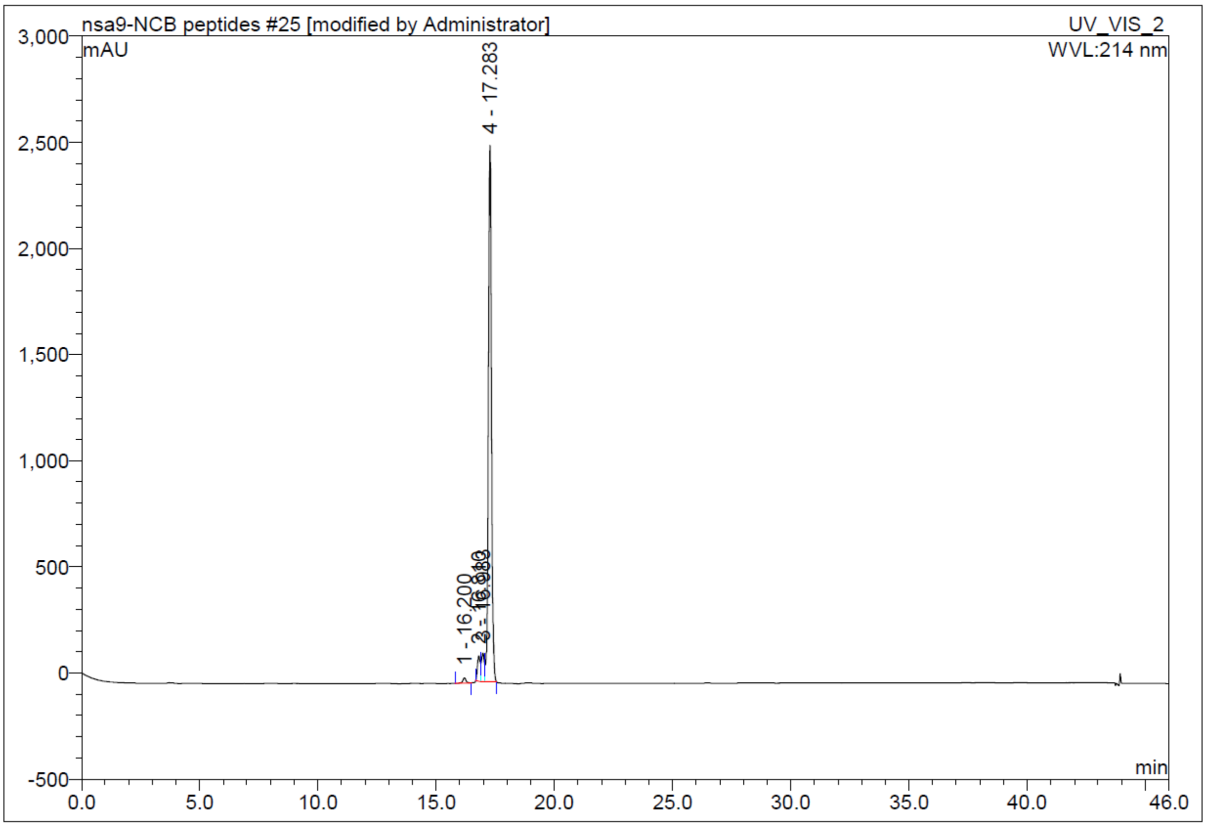

FITC-Ahx-Ser-Glu-Arg-Gly-Lys(Ac)Gly-Gly-Lys-NH$_2$ (H4(1-8)K5Ac): analytical HPLC 5-100% acetonitrile/H$_2$O (0.1% trifluoroacetic acid), 30 min gradient.

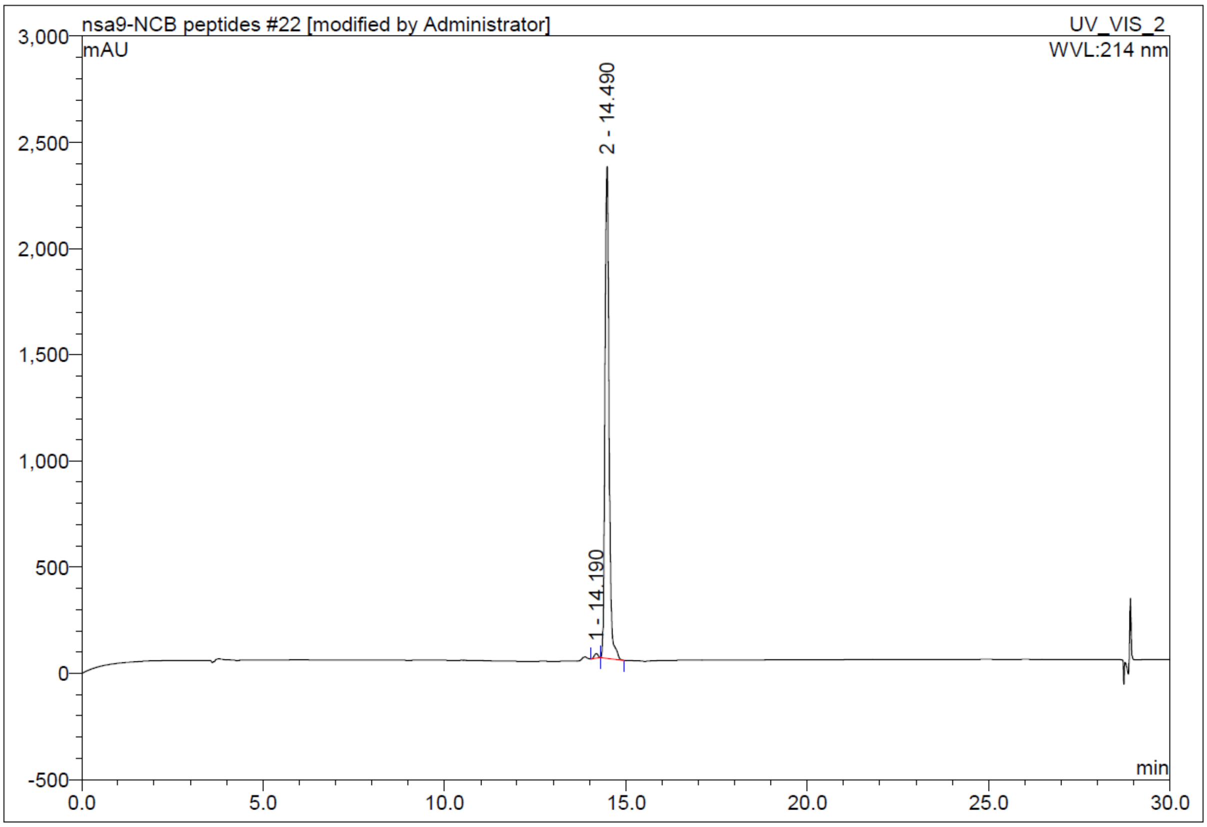

FITC-Ahx-Thr-Ala-Arg-Lys(Ac)-Ser-Thr-Gly-NH_2 (H3(6-12)K9Ac): analytical HPLC 5-100% acetonitrile/H_2O (0.1% trifluoroacetic acid), 15 min gradient.

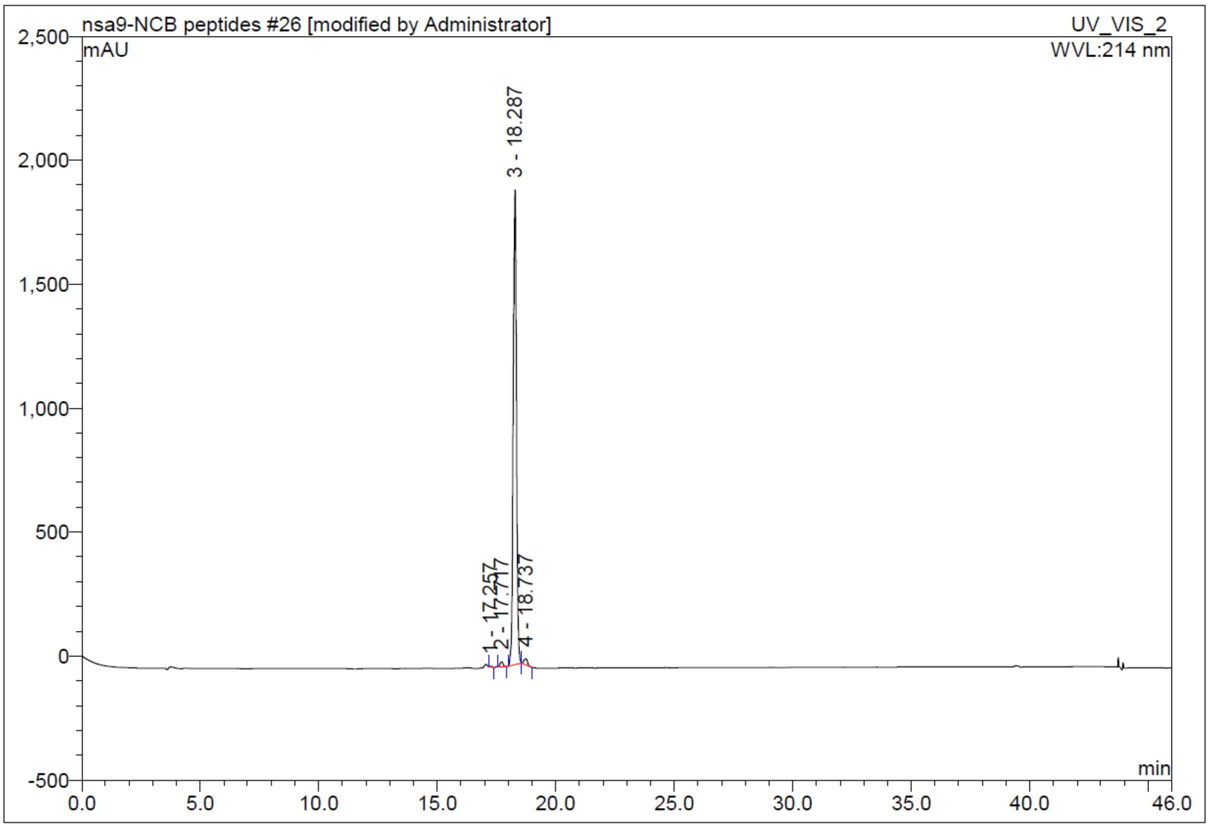

FITC-Ahx-Thr-Ala-Arg-Lys(Ac)-Ser-Thr-Gly-NH$_2$ (H3(6-12)K9Ac): analytical HPLC 5-100% acetonitrile/H$_2$O (0.1% trifluoroacetic acid), 30 min gradient.

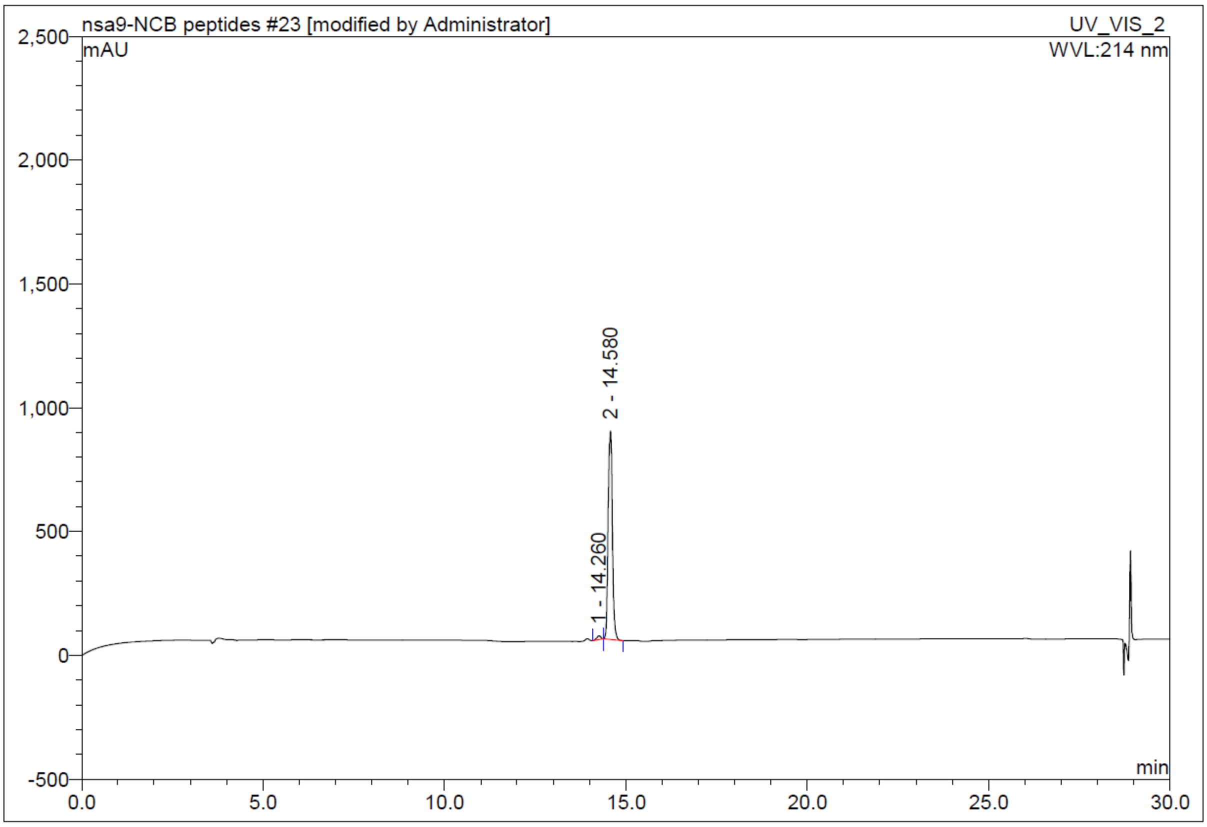

FITC-Ahx-Thr-Gly-Gly-Lys(Ac)-Ala-Pro-Arg-NH_2 (H3(11-17)K14Ac): analytical HPLC 5-100% acetonitrile/H2O (0.1% trifluoroacetic acid), 15 min gradient.

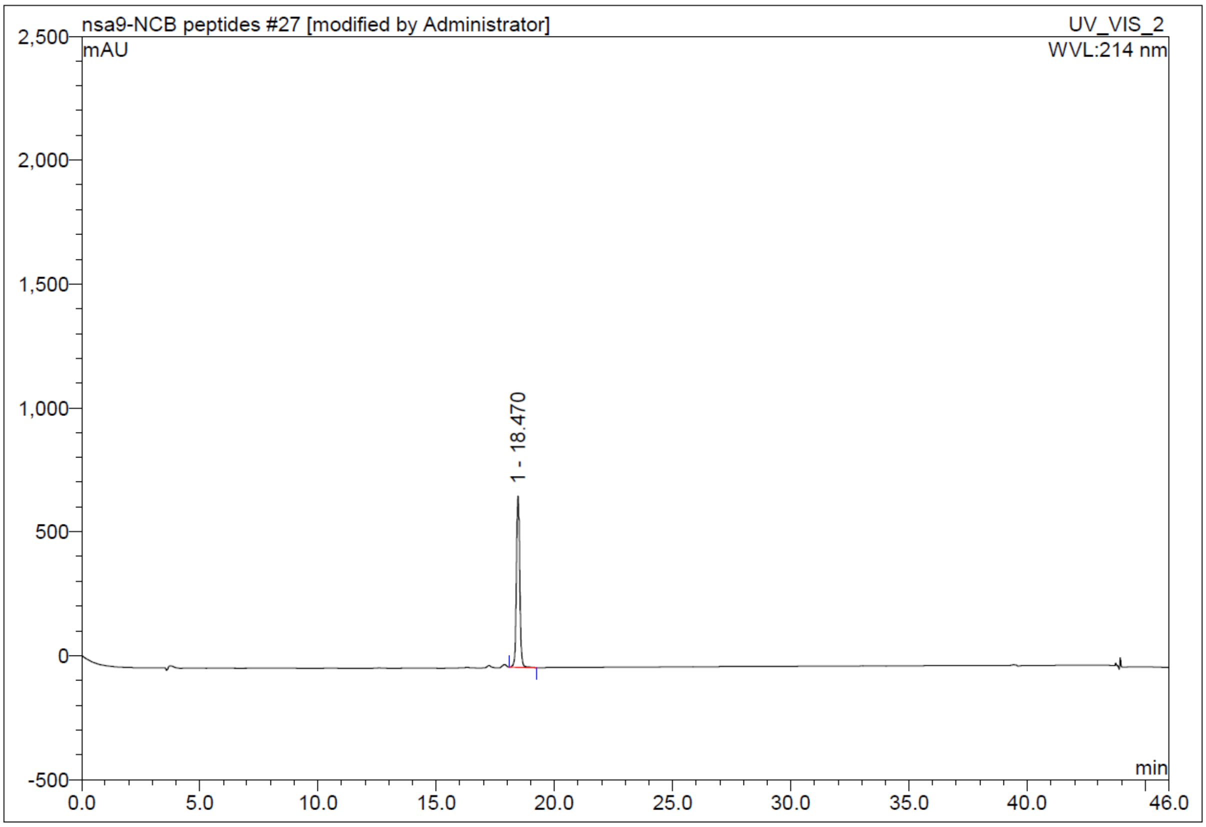

FITC-Ahx-Thr-Gly-Gly-Lys(Ac)-Ala-Pro-Arg-NH2 (H3(11-17)K14Ac): analytical HPLC 5-100% acetonitrile/H2O (0.1% trifluoroacetic acid), 30 min gradient.

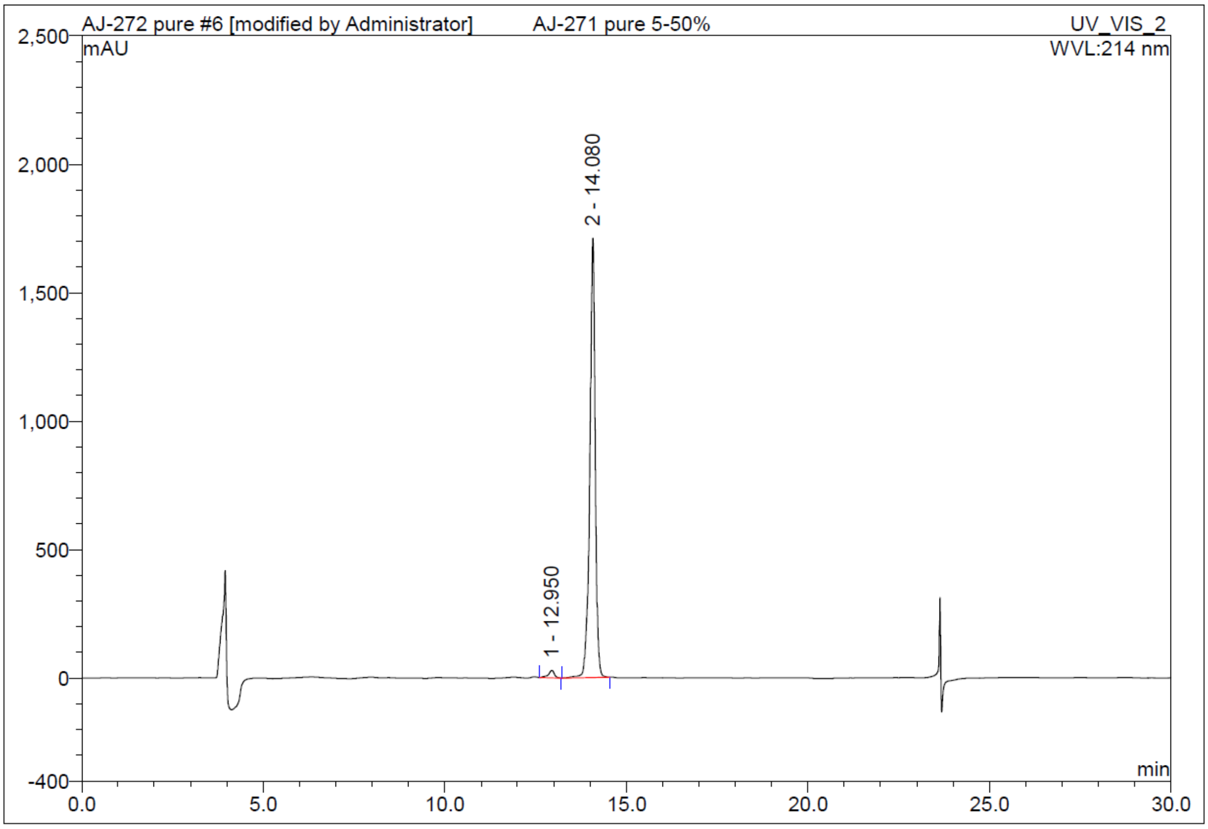

FITC-Ahx-Lys-Ala-Ala-Arg-Lys(Ac)-Ser-Ala-NH2 (H3(23-29)K27Ac): analytical HPLC 5-50% acetonitrile/H2O (0.1% trifluoroacetic acid), 30 min gradient.

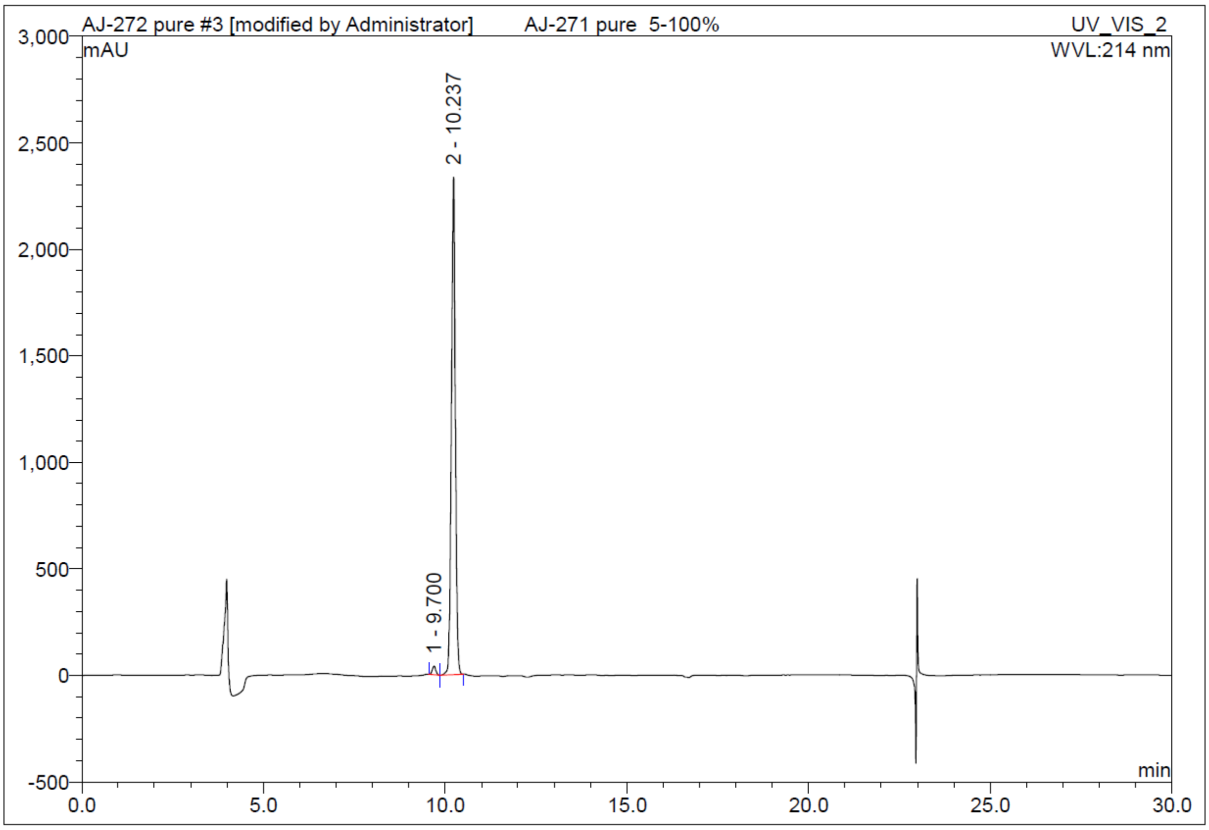

FITC-Ahx-Lys-Ala-Ala-Arg-Lys(Ac)-Ser-Ala-NH2 (H3(23-29)K27Ac): analytical HPLC 5-100% acetonitrile/H2O (0.1% trifluoroacetic acid), 30 min gradient.

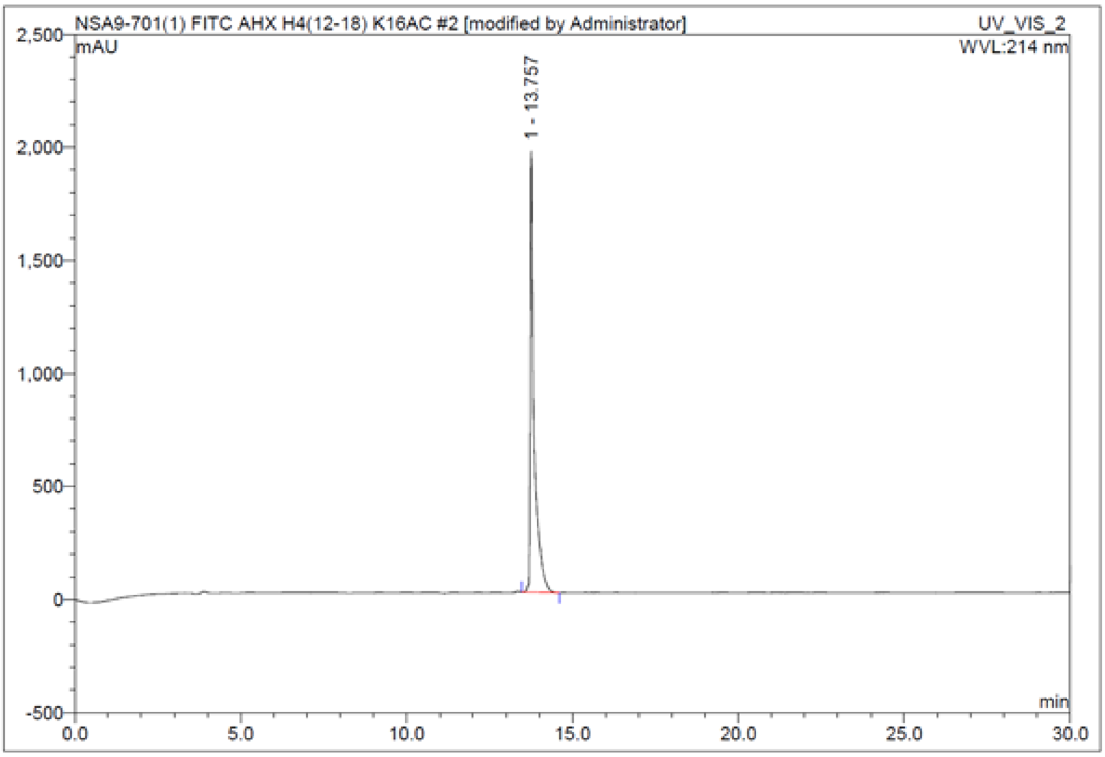

FITC-Ahx-Lys-Gly-Gly-Ala-Lys(Ac)-Arg-His-NH2 (H3(12-18)K16Ac): analytical HPLC 5-100% acetonitrile/H2O (0.1% trifluoroacetic acid), 15 min gradient.

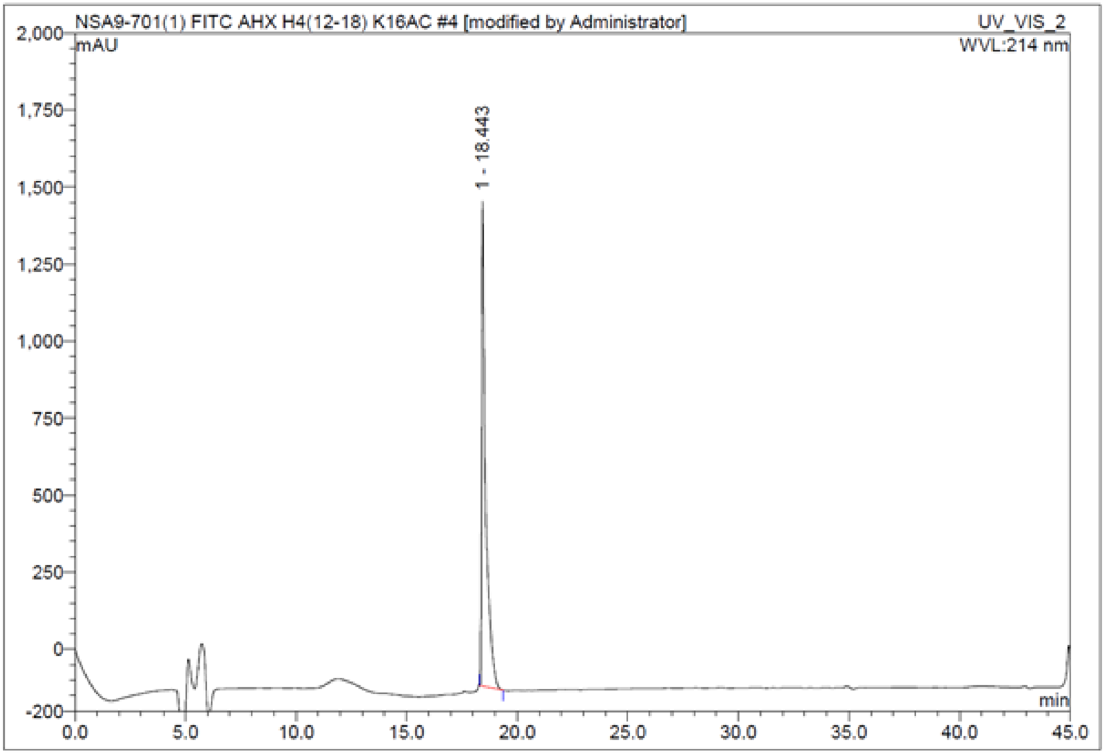

FITC-Ahx-Lys-Gly-Gly-Ala-Lys(Ac)-Arg-His-NH2 (H3(12-18)K16Ac): analytical HPLC 5-100% acetonitrile/H2O (0.1% trifluoroacetic acid), 30 min gradient.

Supplementary References

1. Wang, H. *et al.* Synthetic Inositol Phosphate Analogs Reveal that PPIP5K2 Has a Surface-Mounted Substrate Capture Site that Is a Target for Drug Discovery. *CHBIOL* **21,** 689–699 (2014).
2. Watson, P. J., Fairall, L., Santos, G. M. & Schwabe, J. W. R. Structure of HDAC3 bound to co-repressor and inositol tetraphosphate. *Nature* **481,** 335–340 (2012).
3. Schmitt, L., Spiess, B. & Schlewer, G. Synthesis and binding properties of myo-inositol 4, 5, 6-tris (phosphate) an analogue of myo-inositol 1, 4, 5-tris (phosphate). Correlation with the ionization state of the molecules. *Bioorg Med Chem Lett* (1995).
4. Chung, S. K., Chang, Y. T. & Sohn, K. H. Practical divergent synthesis of all possible regioisomers of myo-inositol trisphosphates. *Chem. Commun.* (1996). doi:10.1039/CC9960000163
5. Podeschwa, M. A. L., Plettenburg, O. & Altenbach, H. J. Flexible Stereo- and Regioselective Synthesis of myo- Inositol Phosphates(Part 2): Via Nonsymmetrical Conduritol B Derivatives. *European Journal of Organic Chemistry* **2005,** 3116–3127 (2005).
6. Gigg, J., Gigg, R., Payne, S. & Conant, R. The allyl group for protection in carbohydrate chemistry. Part 18. Allyl and benzyl ethers of myo -inositol. Intermediates for the synthesis of myo -inositol trisphosphates. *Journal of the Chemical Society, Perkin Transactions 1* **0,** 423–429 (1987).
7. Vidyasagar, A., Pathigoolla, A. & Sureshan, K. M. Chemoselective alcoholysis /acetolysis of trans -ketals over cis -ketals and its application in the total synthesis of the cellular second messenger, d - myo - inositol-1,4,5-trisphosphate. *Organic & Biomolecular Chemistry* **11,** 5443–5453 (2013).
8. Rajput, V. K., Roy, B. & Mukhopadhyay, B. Sulfuric acid immobilized on silica: an efficient reusable catalyst for selective hydrolysis of the terminal O-isopropylidene group of sugar derivatives. *Tetrahedron Letters* (2006).
9. Mills, S. J., Riley, A. M., Liu, C., Mahon, M. F. & Potter, B. V. L. A Definitive Synthesis of D- myo- Inositol 1,4,5,6- Tetrakisphosphate and Its Enantiomer D- myo- Inositol 3,4,5,6- Tetrakisphosphate from a Novel Butane- 2,3- diacetal- Protected Inositol. *Chemistry - A European Journal* **9,** 6207–6214 (2003).
10. Riley, A. M., Wang, H., Weaver, J. D., Shears, S. B. & Potter, B. V. L. First synthetic analogues of diphosphoinositol polyphosphates: interaction with PP-InsP5 kinase. *Chem. Commun.* **48,** 11292–11294 (2012).
11. Bruzik, K. S. & Tsai, M. D. Efficient and systematic syntheses of enantiomerically pure and regiospecifically protected myo-inositols. *J Am Chem Soc* (1992). doi:10.1021/ja00042a011
12. Pietrusiewicz, K. M., Salamończyk, G. M. & Bruzik, K. S. The synthesis of homochiral inositol phosphates from myo-inositol. *Tetrahedron*

(1992).
